# Supplementary material for: A new early actinopterygian from the Mid-Pennsylvanian Logan Quarry Shale member of Indiana
Source: PLoS One. 2025 May 7;20(5):e0320932. doi: 10.1371/journal.pone.0320932 (PMC12057928; doi:10.1371/journal.pone.0320932)
Supplement: S1 File — (DOCX) [file pone.0320932.s001.docx]

**Supplementary Information 1**

***A new early Actinopterygian from the Mid-Pennsylvanian Logan Quarry Shale member of Indiana***

Chenchen Shen^a,b^

^a^ Department of Ecology and Evolutionary Biology, University of Kansas, Lawrence, Kansas, 66045, USA.

^b^ Biodiversity Institute, University of Kansas, Dyche Hall, 1345 Jayhawk Blvd, Lawrence, Kansas, 66045, USA.

Email: c721s275@ku.edu

**Table of Contents**

1. **List of characters used in the phylogenetic analysis**
2. **Character states that support the nodes for the tree presented in Figure S1**
3. **List of characters used in the phylogenetic analysis**

This phylogenetic analysis used the list of characters from Giles et al. [1]. For the 265 characters, 258 characters are unordered, whereas characters 89, 91, 151, 171, 241, 246, and 251 are ordered. The terminology in the character descriptions is updated to follow the terminology in this study.

**1. Large dermal plates:**

**(0)** absent;

**(1)** present.

From Forey [2]; Gardiner [3]; Zhu & Schultze [4]; Zhu et al. [5, 7, 10, 13]; Zhu & Yu [6]; Zhu et al. [7]; Friedman [8]; Brazeau [9]; Friedman & Brazeau [11]; Davis et al. [12]; Brazeau & Friedman [14]; Giles et al. [1, 15].

**2. Sensory lines:**

**(0)** preserved as open grooves;

**(1)** pass through canals.

From Brazeau [9]; Zhu et al. [13]; and Giles et al. [1, 15].

**3. Premaxilla as distinct ossification:**

**(0)** present;

**(1)** absent.

From Hurley et al. [16]; Xu et al. [17]; and Giles et al. [1].

**4. Premaxillae, contact at midline:**

**(0)** present;

**(1)** absent.

From Cloutier & Ahlberg [18]; Taverne [19]; Schultze & Cumbaa [20]; Zhu & Schultze [4]; Zhu & Yu [6]; Cloutier & Arratia [21]; Friedman & Blom [22]; Zhu et al. [7]; Friedman [8]; Long et al. [23]; Swartz [24]; Choo [25]; and Giles et al. [1].

**5. Premaxilla fused at midline:**

**(0)** absent;

**(1)** present.

From Xu et al. [26, 27]; Xu & Zhao [28]; and Giles et al. [1].

**6. Premaxilla:**

**(0)** Reaches or extends past anterior margin of orbit;

**(1)** Confined to region anterior to orbit.

From Friedman [8]; Giles et al. [1, 15].

**7. Premaxilla contributes to orbital margin:**

**(0)** absent;

**(1)** present.

From Cloutier & Ahlberg [18]; Schultze & Cumbaa [20]; Zhu & Schultze [4]; Zhu et al. [5, 7, 10, 13]; Zhu & Yu [6]; Cloutier & Arratia [21]; Friedman [8]; Long et al. [23]; Swartz [24]; Xu & Gao [29]; Xu et al. [17]; and Giles et al. [1].

**8. Teeth on premaxillae:**

**(0)** present;

**(1)** absent.

From Cloutier & Arratia [21], Xu et al. [17]; and Giles et al. [1].

**9. Mobile premaxilla:**

**(0)** absent;

**(1)** present.

From Arratia [30]; Cavin & Suteethorn [31]; Hurley et al. [16]; and Giles et al. [1].

**10. Olfactory nerve pierces premaxilla:**

**(0)** absent;

**(1)** present.

From Grande [32]; Xu et al. [27]; Xu & Shen [33]; Xu & Zhao [28]; and Giles et al. [1].

**11. Nasal process of premaxilla:**

**(0)** absent;

**(1)** short;

**(2)** long, reaches skull roof.

From Gardiner & Schaeffer [34]; Gardiner et al. [35, 36]; Cavin & Suteethorn [31]; Hurley et al. [16]; Grande [32]; Lopez-Arbarello [37]; Xu & Wu [38]; Xu et al. [17]; Xu & Shen [33]; Xu & Zhao [28]; and Giles et al. [1].

**12. Sensory canal on premaxilla:**

**(0)** present;

**(1)** absent.

From Giles et al. [1].

**13. Median dorsal rostral:**

**(0)** present;

**(1)** absent.

From Cloutier & Ahlberg [18]; Taverne [19]; Lund [39]; Schultze & Cumbaa [20]; Zhu & Schultze [4]; Lund & Poplin [40]; Cloutier & Arratia [21]; Friedman & Blom [22]; Long et al. [23]; Swartz [24]; Choo [25]; Xu et al. [17]; and Giles et al. [1].

**14. Single median dermal bone capping snout:**

**(0)** absent;

**(1)** present.

From Gardiner & Schaeffer [34]; Taverne [19]; Friedman & Blom [22]; Long et al. [23]; Swartz [24]; Choo [25]; and Giles et al. [1].

**15. Median rostral:**

**(0)** plate-like;

**(1)** tube-like.

From Gardiner et al. [35]; Hurley et al. [16]; and Giles et al. [1].

**16. Pores for rostral organ:**

**(0)** absent;

**(1)** present.

From Friedman [1]; and Giles et al., 2017.

**17. Nasal bone as single consolidated ossification:**

**(0)** absent;

**(1)** present.

From Taverne [19]; Schultze & Cumbaa [20]; Friedman & Blom [22]; Long et al. [23]; Swartz [24]; Choo [25]; and Giles et al. [1].

**18. Contact of nasals on midline:**

**(0)** separated by dermal bones;

**(1)** contacting or separated by gap unfilled by bone.

From Giles et al. [1].

**19. Nasal contributes to orbital margin:**

**(0)** absent;

**(1)** present.

From Xu & Wu [38]; Xu et al. [17]; Xu & Zhao [28]; and Giles et al. [1].

**20. Mesial margin of (anterior) nasal:**

**(0)** not notched;

**(1)** notched.

From Lund et al. [41]; Ahlberg & Johanson [42]; Ahlberg et al. [43]; Lund [39]; Poplin & Lund [44]; Schultze & Cumbaa [20]; Lund & Poplin [40]; Cloutier & Arratia [21]; Zhu & Ahlberg [45]; Daeschler et al. [46]; Zhu et al. [7, 10]; Choo [25]; and Giles et al. [1].

**21. Posterior nostril in complete communication with orbital fenestra:**

**(0)** absent;

**(1)** present.

From Friedman & Blom [22]; Long et al. [23]; Choo [25]; and Giles et al. [1].

**22. Posterior nostril - contribution to margin by premaxillae:**

**(0)** absent;

**(1)** present.

From Friedman & Blom [22]; Long et al. [23]; Choo [25]; and Giles et al. [1].

**23. Tectals:**

**(0)** absent;

**(1)** present.

From Lund et al. [41]; Cloutier & Ahlberg [18]; Lund [39]; Schultze & Cumbaa [20]; Zhu & Schultze [4]; Zhu et al. [5, 7, 10, 13]; Lund & Poplin [40]; Zhu & Yu [6]; Cloutier & Arratia [21]; Friedman [8]; Swartz [24]; and Giles et al. [1].

**24. Dermal intracranial joint:**

**(0)** absent;

**(1)** present.

From Cloutier & Ahlberg [18]; Ahlberg & Johanson [42]; Zhu & Ahlberg [45]; Zhu & Schultze [4]; Zhu et al. [5, 7, 10, 13]; Zhu & Yu [6]; Daeschler et al. [46]; Friedman [8]; Brazeau [9]; Choo [25]; Davis et al. [12]; and Giles et al. [1].

**25. Pineal foramen:**

**(0)** present;

**(1)** absent.

From Cloutier & Ahlberg [18]; Taverne [19]; Schultze & Cumbaa [20]; Zhu & Schultze [4]; Zhu & Yu [6]; Friedman & Blom [22]; Friedman [8]; Long et al. [23]; Brazeau [9]; Swartz [24]; Davis et al. [12]; Zhu et al. [13]; Xu et al. [17]; Giles et al. [1, 15].

**26. Pineal eminence:**

**(0)** absent;

**(1)** present.

From Friedman [8]; Zhu et al. [10]; and Giles et al. [1].

**27. Shape of postparietals (= parietals):**

**(0)** rectangular, with long axis parallel to midline;

**(1)** quadrate.

From Dietze [47]; Schultze & Cumbaa [20]; Cloutier & Arratia [21]; Friedman & Blom [22]; Long et al. [23]; Swartz [24]; Choo [25]; Xu et al. [17]; and Giles et al. [1].

**28. Relative lengths of parietals (= frontals) and postparietals:**

**(0)** parietals shorter than postparietal;

**(1)** parietals approximately equal to postparietal;

**(2)** parietals longer than postparietal.

From Lund et al. [41]; Taverne [19]; Dietze [47]; Lund [39]; Poplin & Lund [44]; Schultze & Cumbaa [20]; Lund & Poplin [40]; Cloutier & Arratia [21]; Friedman & Blom [22]; Zhu et al. [7]; Long et al. [23]; Swartz [24]; Choo [25]; Lopez-Arbarello [37]; Xu et al. [17]; and Giles et al. [1].

**29. Parietals broad posteriorly and tapering anteriorly:**

**(0)** absent;

**(1)** present.

From Arratia [30]; Lopez-Arbarello [37]; and Giles et al. [1].

**30. Anterior pit line:**

**(0)** absent;

**(1)** present.

From Giles et al. [1, 15].

**31. Otic canal extends through postparietals:**

**(0)** absent;

**(1)** present.

From Giles et al. [1, 15].

**32. Junction between supraorbital and infraorbital canals:**

**(0)** absent;

**(1)** present.

From Giles et al. [1].

**33. Anterior branch of infraorbital sensory canal:**

**(0)** absent;

**(1)** present.

From Giles et al. [1].

**34. Tabular:**

**(0)** present;

**(1)** absent.

From Lund et al. [41]; Cloutier & Ahlberg [18]; Schultze & Cumbaa [20]; Zhu & Schultze [4]; Cloutier & Arratia [21]; Long et al. [23]; Swartz [24]; and Giles et al. [1].

**35. Tabular pit line:**

**(0)** absent ;

**(1)** present.

From Giles et al. [1, 15].

**36. Number of bones carrying otic portion of lateral line canal between ventral dermosphenotic (= dermosphenotic) and posterior edge of skull roof:**

**(0)** at least two;

**(1)** one.

From Gardiner & Schaeffer [34]; Cloutier & Arratia [21]; Hurley et al. [16]; Choo [25]; Xu & Zhao [28]; and Giles et al. [1].

**37. Dorsal dermosphenotic (= Intertemporal) - relative length:**

**(0)** shorter than supratemporal/supratemporotabular;

**(1)** of similar length to supratemporal/supratemporotabular;

**(2)** longer than supratemporal/supratemporotabular.

From Taverne [19]; Friedman & Blom [22]; Choo [25]; and Giles et al. [1].

**38. Dorsal dermosphenotic - contact with supratemporal/supratemporotabular anterior to that between parietal and parietal:**

**(0)** absent;

**(1)** present.

From Friedman & Blom [22]; Choo [25]; and Giles et al. [1].

**39. Dorsal dermosphenotic contacts nasal:**

**(0)** absent;

**(1)** present.

From Xu & Gao [29]; Xu et al. [17]; and Giles et al. [1].

**40. Supratemporal/supratemporotabular - narrow anterolateral flange forming ventral margin of spiracular opening:**

**(0)** absent;

**(1)** present.

From Choo [25]; and Giles et al. [1].

**41. Postparietal fused to supratemporotabular (= dermopterotic):**

**(0)** absent;

**(1)** present.

From Xu & Gao [29]; Xu et al. [17]; and Giles et al. [1].

**42. Bone carrying otic portion of lateral line canal extends past posterior margin of postparietals:**

**(0)** absent;

**(1)** present.

From Lu et al. [48]; and Giles et al. [1].

**43. Number of paired extrascapulae:**

**(0)** one pair;

**(1)** two pairs;

**(2)** three or more pairs.

From Gardiner & Schaeffer [34]; Lund et al. [41]; Cloutier & Ahlberg [18]; Coates [49]; Lund [39]; Poplin & Lund [44]; Schultze & Cumbaa [20]; Zhu & Schultze [4]; Lund & Poplin [40]; Cloutier & Arratia [21]; Friedman & Blom [22]; Long et al. [23]; Swartz [24]; Choo [25]; Lopez-Arbarello [37]; Zhu et al. [13]; Xu et al. [27]; Xu & Zhao [28]; and Giles et al. [1].

**44. Extrascapular reaches lateral edge of skull roof:**

**(0)** absent;

**(1)** present.

From Giles et al. [1, 15].

**45. Single median extrascapular:**

**(0)** present;

**(1)** absent.

From Dietze [47]; Cloutier & Arratia [21]; Long et al. [23]; Swartz [24]; Choo [25]; Xu & Gao [29]; Zhu et al. [13]; Xu et al. [17]; and Giles et al. [1].

**46. Extrascapulae contact each other at midline:**

**(0)** absent;

**(1)** present.

From Giles et al. [1, 15].

**47. Medially-directed branch of sensory canal in extrascapulae:**

**(0)** present;

**(1)** absent.

From Choo [25]; and Giles et al. [1].

**48. Extratemporal:**

**(0)** absent;

**(1)** present.

From Cloutier & Ahlberg [18]; Ahlberg & Johanson [42]; Zhu & Schultze [4]; Zhu et al. [5, 7, 10]; Zhu & Yu [6]; Zhu & Ahlberg [45]; Daeschler et al. [46]; Friedman [8]; and Giles et al. [1].

**49. Antorbital bone:**

**(0)** absent;

**(1)** present.

From Gardiner & Schaeffer [34]; Lund [39]; Cloutier & Arratia [21]; Hurley et al. [16]; Choo [25]; Xu & Gao [29]; Xu et al. [17]; and Giles et al. [1].

**50. Tube-like canal bearing anterior arm of antorbital:**

**(0)** absent;

**(1)** present.

From Grande [32]; Xu & Wu [38]; Xu et al. [17]; Xu & Shen [33]; Xu & Zhao [28]; and Giles et al. [1].

**51. Infraorbitals:**

**(0)** one;

**(1)** two;

**(2)** more than two.

From Cloutier & Arratia [21]; Gardiner et al. [36]; Choo [25]; Xu & Gao [29]; Xu et al. [17]; and Giles et al. [1].

**52. Anterior expansion of lacrimal:**

**(0)** absent;

**(1)** present.

From Taverne [19]; Friedman & Blom [22]; Long et al. [23]; Swartz [24]; Choo [25]; and Giles et al. [1].

**53. Notch in anterior margin of jugal:**

**(0)** absent;

**(1)** present.

From Cloutier & Arratia [21]; Friedman & Blom [22]; Long et al. [23]; Swartz [24]; Choo [25]; Xu et al. [17]; and Giles et al. [1].

**54. Suborbital(s):**

**(0)** absent;

**(1)** one;

**(2)** two;

**(3)** three or more.

From Gardiner & Schaeffer [34]; Taverne [19]; Schultze & Cumbaa [20]; Friedman & Blom [22]; Long et al. [23]; Choo [25]; Lopez-Arbarello [37]; Xu & Gao [29]; Xu et al. [27]; Xu & Zhao [28]; and Giles et al. [1].

**55. Multiple rami of infraorbital canal in jugal:**

**(0)** absent;

**(1)** present.

From Giles et al. [1, 15].

**56. Dermosphenotic with distinct posterior ramus:**

**(0)** absent;

**(1)** present.

From Gardiner & Schaeffer [34]; Coates [49]; Schultze & Cumbaa [20]; Cloutier & Arratia [21]; Friedman & Blom [22]; Zhu et al. [7, 10]; Long et al. [23]; Choo [25]; Xu et al. [27]; and Giles et al. [1].

**57. Ventral dermosphenotic- contact with parietals blocked by dorsal dermosphenotic or supratemporotabular:**

**(0)** absent;

**(1)** present.

From Friedman & Blom [22]; Choo [25]; and Giles et al. [1].

**58. Supraorbital:**

**(0)** absent;

**(1)** one or two;

**(2)** three or more.

From Gardiner & Schaeffer [34]; Hurley et al. [16]; Xu & Gao [29]; Xu et al. [17, 27]; Xu & Zhao [28]; and Giles et al. [1].

**59. Anterior-most infraorbital anterior to orbit (i.e. does not contribute to orbital margin):**

**(0)** absent;

**(1)** present.

From Cavin & Suteethorn [31]; Lopez-Arbarello [37]; and Giles et al. [1].

**60. Three or more lachrymals:**

**(0)** absent;

**(1)** present.

From Grande [32]; Xu & Wu [38]; Xu et al. [17, 27]; Xu & Zhao [28]; and Giles et al. [1].

**61. Circumorbital ring:**

**(0)** Supraorbitals do not contact infraorbitals at the anterior rim of the orbit;

**(1)** Supraorbitals contact infraorbitals, closing the orbit.

From Wiley [52]; Lopez-Arbarello [37]; and Giles et al. [1].

**62. Jugal canal:**

**(0)** absent;

**(1)** present.

From Patterson, [51]; Lauder & Liem [52]; Gardiner [3]; Cloutier & Arratia [21]; Brazeau [9]; Friedman & Brazeau [11]; Choo [25]; Davis et al. [12]; Zhu et al. [13]; and Giles et al. [1, 15].

**63. Dermohyal:**

**(0)** absent;

**(1)** present.

From Patterson [51]; Gardiner & Schaeffer [34]; Lund et al. [41]; Cloutier & Ahlberg [18]; Coates [49]; Dietze [47]; Lund [39]; Schultze & Cumbaa [20]; Zhu & Schultze [4]; Zhu et al. [5, 7, 10]; Lund & Poplin [40]; Zhu & Yu [6]; Cloutier & Arratia [21]; Gardiner et al. [36]; Friedman & Blom [22]; Friedman [8]; Long et al. [23]; Swartz [24]; Choo [25]; Xu & Gao [29]; Xu et al. [17, 27]; Xu & Zhao [28]; and Giles et al. [1].

**64. Head of dermohyal projects above dorsal margin of opercle:**

**(0)** absent;

**(1)** present.

From Giles et al. [1, 15].

**65. Dermohyal:**

**(0)** fused to hyomandibular;

**(1)** separate from hyomandibular.

From Gardiner et al. [36]; Coates [50]; Xu & Gao [29]; Xu et al. [17]; and Giles et al. [1].

**66. Complete enclosure of spiracle by bones bearing otic and infraorbital canals:**

**(0)** absent;

**(1)** present.

From Friedman [8]; Zhu et al. [10]; and Giles et al. [1].

**67. Maxilla:**

**(0)** absent;

**(1)** present.

From Zhu & Yu [6]; Friedman [8]; Xu et al. [17, 27]; Xu & Zhao [28]; Giles et al. [1].

**68. Expanded dorsal lamina of maxilla:**

**(0)** absent;

**(1)** present.

From Lund et al. [41]; Lund [39]; Poplin & Lund [44]; Schultze & Cumbaa [20]; Zhu & Schultze [4]; Zhu et al. [5, 7, 10, 13]; Zhu & Yu [6]; Lund & Poplin [40]; Cloutier & Arratia [21]; Friedman [8]; and Giles et al. [1, 15].

**69. Contribution by maxilla to posterior margin of cheek:**

**(0)** absent;

**(1)** present.

From Friedman [8]; Zhu et al. [10, 13]; and Giles et al. [1, 15].

**70. Sensory canal/pit line associated with maxilla:**

**(0)** absent;

**(1)** present.

From Friedman [8]; Zhu et al. [10, 13]; and Giles et al. [1].

**71. Teeth on maxilla:**

**(0)** present;

**(1)** absent.

From Cloutier & Arratia [21]; Lopez-Arbarello [37]; Xu et al. [17, 27]; Xu & Zhao [28]; and Giles et al. [1].

**72. Mobile maxilla in cheek:**

**(0)** absent;

**(1)** present.

From Gardiner & Schaeffer [34]; Gardiner et al. [35, 36]; Coates, 1999; Hurley et al. [16]; Xu & Gao [19]; Xu et al. [17, 27]; Xu & Zhao [28]; and Giles et al. [1].

**73. Peg-like anterior process of maxilla:**

**(0)** absent;

**(1)** present.

From Grande [32]; Xu & Wu [38]; Xu et al. [17]; and Giles et al. [1].

**74. Posterior maxillary notch:**

**(0)** absent;

**(1)** present.

From Grande & Bemis [53]; Xu & Wu [38]; Xu et al. [17, 27]. Arratia [54]; Xu & Zhao [28]; and Giles et al. [1].

**75. Supramaxilla:**

**(0)** absent;

**(1)** one;

**(2)** two.

From Gardiner & Schaeffer [34]; Gardiner et al. [35]; Gardiner et al. [36]; Coates [50]; Hurley et al. [16]; Xu & Gao [29]; Xu et al. [17, 27]; Xu & Shen [33]; Xu & Zhao [28]; and Giles et al. [1].

**76. Course of mandibular canal:**

**(0)** traces ventral margin of jaw along entire length;

**(1)** arches dorsally in anterior half of jaw.

From Friedman & Blom [22]; Long et al. [23]; Swartz [24]; Choo [25]; and Giles et al. [1].

**77. Mandibular canal reaches anterior margin of mandible:**

**(0)** present;

**(1)** absent.

From Giles et al. [1, 15].

**78. Mandibular canal:**

**(0)** primarily carried by infradentaries;

**(1)** primarily carried by dentary.

From Patterson [51]; Cloutier & Ahlberg [18]; Coates [49]; Schultze & Cumbaa [20]; Zhu & Schultze [4]; Zhu et al. [5, 7, 10, 13]; Zhu & Yu [6]; Cloutier & Arratia [21]; Friedman [8] ; Choo [25]; and Giles et al. [1].

**79. Relative length of dentary:**

**(0)** long (constitutes most of the length of the lower jaw);

**(1)** short (constitutes less than half of jaw length).

From Ahlberg & Johanson, 1998; Zhu et al. [5, 10]; Zhu & Yu [6]; Zhu & Ahlberg [45]; Friedman [8]; and Giles et al. [1].

**80. Teeth on dentary:**

**(0)** present;

**(1)** absent.

From Cloutier & Arratia [21], Xu et al. [17]; and Giles et al. [1].

**81. Dentary with conspicuously reflexed distal tip:**

**(0)** absent;

**(1)** present.

From Friedman & Blom [22]; Long et al. [23]; Swartz [24]; Choo [25]; and Giles et al. [1].

**82. Enlarged series of parasymphysial teeth on dentary:**

**(0)** absent;

**(1)** present.

From Friedman & Blom [22]; Long et al. [23]; Swartz [24]; Choo [25]; and Giles et al. [1].

**83. Facet for parasymphysial tooth-whorl on anterior dentary:**

**(0)** present;

**(1)** absent.

From Choo [25]; and Giles et al. [1].

**84. Teeth of outer dental arcade:**

**(0)** several rows of disorganized teeth;

**(1)** two rows, with large teeth lingually and small teeth labially;

**(2)** single row of teeth.

From Friedman [8]; and Giles et al. [1].

**85. Jaw margins overlain by lateral lamina:**

**(0)** absent;

**(1)** present.

From Giles et al. [1].

**86. Acrodin caps on teeth:**

**(0)** absent;

**(1)** present.

From Patterson [51]; Gardiner [4]; Maisey [55]; Gardiner & Schaeffer [34]; Cloutier & Ahlberg [18]; Taverne [19]; Coates [50]; Poplin & Lund [40]; Schultze & Cumbaa [20]; Zhu & Schultze [4]; Zhu et al. [5, 7, 10, 13]; Zhu & Yu [6]; Cloutier & Arratia [21]; Gardiner et al. [36]; Friedman & Blom [22]; Friedman [8]; Long et al. [23]; Friedman & Brazeau [11]; Choo [25]; Xu & Gao [29]; Xu et al. [17]; and Giles et al. [1, 15].

**87. Plicidentine:**

**(0)** absent;

**(1)** present.

From Zhu & Yu [6]; Friedman [8]; Lopez-Arbarello [37]; and Giles et al. [1].

**88. Ossification of mentomeckelian region:**

**(0)** present;

**(1)** absent.

From Friedman & Blom [22]; Long et al. [23]; Swartz [24]; Grande [32]; Choo [25]; Xu et al. [17]; and Giles et al. [1].

**89. Number of infradentaries:**

**(0)** more than two;

**(1)** two (angular and surangular);

**(2)** one (angular only).

From Friedman & Blom [22]; Friedman [8]; Long et al. [23]; Choo [25]; Xu & Gao [29]; Xu et al. [17]; and Giles et al. [1].

**90. Coronoids:**

**(0)** present;

**(1)** absent.

From Schultze and Cumbaa [20]; Zhu and Schultze [4]; Zhu et al. [5, 7, 10]; Zhu and Yu [6]; Friedman [8]; and Giles et al. [1].

**91. Number of coronoids:**

**(0)** five;

**(1)** four or more;

**(2)** three;

**(3)** two;

**(4)** one.

From Ahlberg & Clack [56]; Daeschler et al. [46]; Friedman [8]; Zhu et al. [10, 13]; and Giles et al. [1, 15].

**92. Posterior coronoid:**

**(0)** morphologically similar to anterior coronoids;

**(1)** expanded.

From Cloutier & Ahlberg [18]; Ahlberg & Johanson [42]; Zhu & Ahlberg [45]; Daeschler et al. [46]; and Giles et al. [1].

**93. Coronoid process of lower jaw:**

**(0)** absent;

**(1)** present.

From Gardiner & Schaeffer [34]; Zhu & Yu [6]; Friedman [8]; Xu & Gao [29]; Xu et al. [17, 27]; Xu & Zhao [28]; and Giles et al. [1].

**94. Coronoid process contributed to by:**

**(0)** prearticular only;

**(1)** surangular only;

**(2)** dentary plus postdentary bones;

**(3)** angular only.

From Gardiner et al. [36]; and Giles et al. [1].

**95. Leptolepid notch:**

**(0)** absent;

**(1)** present.

From Arratia [54]; and Giles et al. [1].

**96. Symplectic involvement in jaw joint:**

**(0)** absent;

**(1)** present.

From Grande & Bemis [53]; Grande [32]; Xu & Wu [38]; Xu et al. [17, 27]; Lopez-Arbarello [37]; Xu & Zhao [28]; and Giles et al. [1].

**97. Retroarticular process:**

**(0)** present;

**(1)** absent.

From Friedman [8]; and Giles et al. [1].

**98. Palatal bite:**

**(0)** absent;

**(1)** present.

From Giles et al. [1].

**99. Palatal articulation with basipterygoid process:**

**(0)** articulation not obvious;

**(1)** via basipterygoid fenestra;

**(2)** via metapterygoid process/notch.

From Friedman [8]; Brazeau [9]; Zhu et al. [10, 13]; Friedman & Brazeau [11]; Davis et al. [12]; and Giles et al. [1, 15].

**100. Palatoquadrate ossifications:**

**(0)** comineralized;

**(1)** separate ossification centers.

From Giles et al. [1].

**101. Lateral process of ectopterygoid:**

**(0)** absent;

**(1)** present.

From Giles et al. [1].

**102. Palatoquadrate symphysis:**

**(0)** absent;

**(1)** present.

From Giles et al. [1].

**103. Dorsal margin of palate:**

**(0)** high posterior extension;

**(1)** flat dorsal margin.

From Giles et al. [1].

**104. Metapterygoid posterior to quadrate:**

**(0)** absent;

**(1)** present.

From Giles et al. [1].

**105. Number of dermopalatines:**

**(0)** multiple;

**(1)** single.

From Friedman [8]; and Giles et al. [1].

**106. Prearticular:**

**(0)** present;

**(1)** absent.

From Giles et al. [1].

**107. Vomers:**

**(0)** paired;

**(1)** single.

From Lopez-Arbarello [37]; Arratia [54]; Xu & Wu [38]; Xu & Zhao [28]; and Giles et al. [1].

**108. Vomer sutured to parasphenoid:**

**(0)** absent;

**(1)** present.

From Hurley et al. [16]; and Giles et al. [1].

**109. Accessory opercle:**

**(0)** absent;

**(1)** present.

From Schultze & Cumbaa [20]; Cloutier & Arratia [21]; Friedman & Blom [22]; Long et al. [23]; Swartz [24]; and Giles et al. [1].

**110. Opercle - relative size:**

**(0)** at least twice as high as subopercle;

**(1)** subequal;

**(2)** smaller than subopercle.

From Lund et al. [41]; Lund [39]; Lund & Poplin [40]; Cloutier & Arratia [21 Long et al. [23]; Swartz [24]; Choo [25]; Xu et al. [27]; Xu & Zhao [28]; and Giles et al. [1].

**111. Subopercle:**

**(0)** present;

**(1)** absent.

From Xu et al. [17]; and Giles et al. [1].

**112. Anterodorsal process of subopercle:**

**(0)** absent;

**(1)** present.

From Long et al. [23]; Choo [25]; Lopez-Arbarello [37]; and Giles et al. [1].

**113. Anteroventral process of subopercle:**

**(0)** absent;

**(1)** present.

From Giles et al. [1].

**114. Number of cheek bones bearing pre-opercular canal posterior to jugal:**

**(0)** one;

**(1)** multiple;

**(2)** series of small ossicles.

From Friedman [8]; Zhu et al. [10, 13]; Xu & Zhao [28]; and Giles et al. [1].

**115. Preopercle orientation:**

**(0)** prounounced dorsal limb;

**(1)** vertical;

**(2)** pronounced ventral limb.

From Gardiner et al. [36]; Swartz [24]; and Giles et al. [1].

**116. Junction between preopercular and more anterior cheek bones:**

**(0)** Infraorbitals (including jugal) or suborbitals suture with or abut preopercular;

**(1)** Infraorbitals (including jugals) and suborbitals broadly overlap preopercular.

From Lopez-Arbarello [37]; and Giles et al. [1].

**117. Posterior border of preopercle notched ventrally:**

**(0)** absent;

**(1)** present.

From Lopez-Arbarello [37]; and Giles et al. [1].

**118. Interopercle:**

**(0)** absent;

**(1)** present.

From Gardiner & Schaeffer [34]; Olsen & McCune [57]; Gardiner et al. [35, 36]; Cavin & Suteethorn [31]; Hurley et al. [16]; Lopez-Arbarello [37]; Xu & Zhao [28]; and Giles et al. [1].

**119. Branchiostegal rays - dorsal-most in series:**

**(0)** of similar depth to adjacent branchiostegal ray;

**(1)** deeper than adjacent branchiostegal ray.

From Lund et al. [41]; Cloutier & Arratia [21]; Choo [25]; and Giles et al. [1].

**120. Lateral gulars:**

**(0)** present;

**(1)** absent.

From Xu et al. [17]; and Giles et al. [1].

**121. Lateral gulars:**

**(0)** extending most of the length of the lower jaw;

**(1)** restricted to the anterior third of the lower jaw (no longer than the width of three

branchiostegals).

From Gardiner & Schaeffer [34]; Cloutier & Ahlberg [18]; Taverne [19]; Lund & Poplin [58]; Coates [50]; Schultze & Cumbaa [20]; Zhu & Schultze [4]; Cloutier & Arratia [21]; Friedman & Blom [22]; Long et al. [23]; Swartz [24]; Brazeau [9]; Xu & Gao [29]; Davis et al. [12]; Zhu et al. [13]; Xu et al. [17]; and Giles et al. [1, 15].

**122. Median gular:**

**(0)** absent;

**(1)** present.

From Lund et al. [41]; Cloutier & Ahlberg [18]; Coates [50]; Lund [39]; Schultze & Cumbaa [20]; Zhu & Schultze [4]; Zhu et al. [5, 7, 10, 13]: Lund & Poplin [40]; Zhu & Yu [6]; Cloutier & Arratia [21]; Friedman [8]; Xu & Gao [29]; Xu et al. [17, 27]; Xu & Zhao [28]; and Giles et al. [1, 15].

**123. Relative length of median gular:**

**(0)** much shorter than jaw length;

**(1)** more than half of jaw length.

From Giles et al. [1, 15].

**124. Fenestra ventrolateralis:**

**(0)** absent;

**(1)** present.

From Schultze & Cumbaa [20]; Zhu & Schultze [4]; Zhu et al. [5, 7, 10, 13]; Zhu & Yu [6]; Friedman [8]; and Giles et al. [1].

**125. Palatal opening surrounded by premaxilla, maxilla, dermopalatine and vomer:**

**(0)** absent;

**(1)** present.

From Zhu & Yu [6]; Friedman [8]; and Giles et al. [1].

**126. Internasal cavity:**

**(0)** absent;

**(1)** present.

From Ahlberg & Johanson [42]; Zhu & Yu [6]; Zhu & Ahlberg [45]; Daeschler et al. [46]; Long et al. [23]; Friedman [8]; Zhu et al. [10, 13]; and Giles et al. [1, 15].

**127. Interorbital septum:**

**(0)** broad;

**(1)** narrow.

From Friedman [8]; Zhu et al. [10, 13]; Brazeau [9]; Friedman & Brazeau [11]; Davis et al. [12]; and Giles et al. [1, 15].

**128. Optic foramen:**

**(0)** dorsally positioned;

**(1)** ventrally positioned.

From Giles et al. [1].

**129. Pronounced median anterior crista on dorsal surface of braincase:**

**(0)** absent;

**(1)** present.

From Giles et al. [1, 15].

**130. Expanded anterior dorsal fontanelle:**

**(0)** absent;

**(1)** present.

From Giles et al. [1, 15].

**131. Endoskeletal intracranial joint:**

**(0)** absent;

**(1)** present.

From Cloutier & Ahlberg [18]; Ahlberg & Johanson [42]; Zhu & Ahlberg [45]; Zhu et al. [5, 10, 13]; Zhu & Yu [6]; Daeschler et al. [46]; Long et al. [23]; Friedman [8]; Brazeau [9]; Friedman & Brazeau [11]; Davis et al. [12]; and Giles et al. [1, 15].

**132. Eye stalk or unfinished area for similar structure:**

**(0)** absent;

**(1)** present.

From Zhu & Schultze [4]; Zhu et al. [5, 7, 10, 13]; Zhu & Yu [6]; Friedman [8]; and Giles et al. [1, 15].

**133. Roof of posterior myodome perforated by palatine branch of facial nerve (VII):**

**(0)** absent;

**(1)** present.

From Coates [50]; and Giles et al. [1].

**134. Foramen for abducens nerve (VI) dorsally positioned (level with optic foramen (II)):**

**(0)** absent;

**(1)** present.

From Coates [50]; and Giles et al. [1].

**135. Anterodorsal myodome:**

**(0)** paired;

**(1)** single;

**(2)** absent.

From Gardiner et al. [35]; Coates [50]; Hurley et al. [16]; Xu & Gao [29]; Xu et al. [17, 27]; Xu & Zhao [28]; and Giles et al. [1].

**136. Posterior myodome:**

**(0)** absent;

**(1)** paired;

**(2)** median.

From Wiley [52]; Gardiner [3]; Gardiner & Schaeffer [34]; Gardiner et al. [35]; Hurley et al. [16]; Lopez-Arbarello [37]; Xu & Gao [29]; Xu et al. [17, 27]; Xu & Zhao [28]; and Giles et al. [1].

**137. Basicranial fenestra:**

**(0)** absent;

**(1)** present.

From Ahlberg & Johanson [42]; Zhu et al. [5, 10, 13]; Zhu & Yu [6]; Zhu & Ahlberg [45]; Friedman [8]; and Giles et al. [1, 15].

**138. Spiracle:**

**(0)** open;

**(1)** partial closure or spiracular bar;

**(2)** complete enclosure in canal.

From Patterson [51]; Gardiner [3]; Gardiner & Schaeffer [34]; Taverne [19]; Coates [50]; Gardiner et al. [36]; Xu & Gao [29]; Xu et al. [17]; and Giles et al. [1].

**139. Basipterygoid process:**

**(0)** present;

**(1)** absent.

From Gardiner et al. [36]; Xu & Gao [29]; Xu et al. [17, 27]; Xu & Zhao [28]; and Giles et al. [1].

**140. Basipterygoid process with vertically oriented component:**

**(0)** absent;

**(1)** present.

From Ahlberg & Johanson [42]; Zhu & Schultze [4]; Zhu et al. [5, 7, 10, 13]; Zhu & Yu [6]; Zhu & Ahlberg [45]; Friedman [8]; Davis et al. [12]; and Giles et al. [1, 15].

**141. Dermal component to basipterygoid process:**

**(0)** absent;

**(1)** present.

From Gardiner [3]; Gardiner & Schaeffer [34]; Taverne [19]; Coates [50]; and Giles et al. [1].

**142. Hyoid facet:**

**(0)** directed posteroventrally;

**(1)** horizontal.

From Gardiner et al., 1996; Gardiner et al., 2005; Hurley et al., 2007; Xu & Gao, 2011; Xu et al., 2015; and Giles et al. [1].

**143. Fossa bridge:**

**(0)** absent;

**(1)** present.

From Gardiner [3]; Gardiner & Schaeffer [34]; Taverne [19]; Coates [50]; Xu & Gao [29]; Xu et al. [17]; and Giles et al. [1].

**144. Posttemporal fossae:**

**(0)** absent;

**(1)** present.

From Zhu & Yu [6]; Friedman [8]; and Giles et al. [1].

**145. Vestibular fontanelle:**

**(0)** absent;

**(1)** present.

From Friedman [8]; Brazeau [9]; Zhu et al. [10, 13]; Friedman & Brazeau [11]; Davis et al. [12]; Brazeau & Friedman [14]; and Giles et al. [1, 15].

**146. Ventral cranial fissure and vestibular fontanelle:**

**(0)** separated by bridge of bone;

**(1)** confluent.

From Coates [50]; and Giles et al. [1].

**147. Accessory fenestration in otic capsule:**

**(0)** absent;

**(1)** present.

From Friedman [8]; Zhu et al. [10]; and Giles et al. [1].

**148. Otoccipital fissure:**

**(0)** absent;

**(1)** present.

From Friedman [8]; Brazeau [9]; Davis et al. [12]; Zhu et al. [13]; and Giles et al. [1, 15].

**149. Median projection overhanging posterior part of posterior dorsal fontanelle:**

**(0)** absent;

**(1)** present.

From Giles et al. [1, 15].

**150. Median projection overhanging anterior part of posterior dorsal fontanelle:**

**(0)** absent;

**(1)** present.

From Giles et al. [1, 15].

**151. Dorsal aorta:**

**(0)** open in groove;

**(1)** canal notched posteriorly;

**(2)** completely enclosed in canal.

From Coates & Sequeira [59]; Coates & Sequeira [60]; Coates [50]; Friedman [8]; Zhu et al. [10, 13]; Friedman & Brazeau [11]; and Giles et al. [1, 15].

**152. Dorsal aorta pierced by canal/s for exit of eff.a.2:**

**(0)** absent;

**(1)** present.

From Giles et al. [1, 15].

**153. Dorsal aorta pierced by canal/s for exit of eff.a.1:**

**(0)** absent;

**(1)** present.

From Giles et al. [1, 15].

**154. Bifurcation of dorsal aorta:**

**(0)** posterior to occiput;

**(1)** anterior to occiput.

From Coates & Sequeira [59]; Coates [50]; Coates & Sequeira [60]; Friedman [8]; Zhu et al. [10, 13]; Friedman & Brazeau [11]; and Giles et al. [1, 15].

**155. Birfurcation of dorsal aorta into lateral dorsal aortae:**

**(0)** open;

**(1)** enclosed in canal.

From Coates [50]; and Giles et al. [1].

**156. Braincase ossifications differentiated:**

**(0)** absent;

**(1)** present.

From Giles et al. [1].

**157. Basisphenoid:**

**(0)** present;

**(1)** absent or very reduced.

From Wiley [52]; Lopez-Arbarello [37]; and Giles et al. [1].

**158. Opisthotic-pterotic relationship:**

**(0)** opisthotic larger than subotic;

**(1)** opisthotic and pterotic equal in size.

From Gardiner et al. [35]; Hurley et al. [16]; and Giles et al. [1].

**159. Epioccipital:**

**(0)** present;

**(1)** absent.

From Hurley et al. [16]; and Giles et al. [1].

**160. Forward extension of the exoccipital around the vagus nerve:**

**(0)** absent;

**(1)** present.

From Olsen & McCune [57]; Gardiner et al. [35]; Cavin & Suteethorn [31]; Hurley et al. [16]; Lopez-Arbarello [37]; and Giles et al. [1].

**161. Spenotic with small dermal component:**

**(0)** absent;

**(1)** present.

From Grande [32]; Lopez-Arbarello [37]; Xu & Wu [38]; Xu et al. [17, 27]; Arratia [54]; Xu & Zhao [28]; and Giles et al. [1].

**162. Pterotic:**

**(0)** present;

**(1)** absent.

From Gardiner et al. [35]; Grande & Bemis [53]; Hurley et al. [16]; Xu et al. [17]; Xu & Shen [33]; Xu & Zhao [28]; and Giles et al. [1].

**163. Opisthotic bone:**

**(0)** present;

**(1)** absent.

From Wiley [52]; Cavin & Suteethorn [31]; Hurley et al. [16]; Grande [32]; Lopez-Arbarello [37]; Xu et al. [17, 27]; Xu & Zhao [28]; and Giles et al. [1].

**164. Intercalar:**

**(0)** present;

**(1)** absent.

From Olsen [61]; Gardiner et al. [35]; Lopez-Arbarello [37]; Xu et al. [17]; Xu & Shen [33]; Xu & Zhao [28]; and Giles et al. [1].

**165. Supraoccipital bone:**

**(0)** absent;

**(1)** present.

From Grande [32]; Xu et al. [17, 27]; Xu & Zhao [28]; and Giles et al. [1].

**166. Membranous outgrowth of intercalary:**

**(0)** absent;

**(1)** present.

From Gardiner et al. [35]; Hurley et al. [16]; and Giles et al. [1].

**167. Post-temporal fossa:**

**(0)** absent;

**(1)** present.

From Gardiner [3]; Gardiner & Schaeffer [34]; Coates [50]; Hurley et al. [16]; Lopez-Arbarello [37]; Xu & Gao [29]; Xu et al. [17, 27]; Xu & Zhao [28]; and Giles et al. [1].

**168. Sub-temporal fossa:**

**(0)** absent;

**(1)** present.

From Gardiner [3]; Gardiner & Schaeffer [34]; Gardiner et al. [35]; Hurley et al. [16]; Xu & Gao [29]; Xu et al. [17, 27]; Xu & Zhao [28]; and Giles et al. [1].

**169. Dilatator fossa:**

**(0)** absent;

**(1)** present.

From Gardiner [3]; Gardiner & Schaeffer [34]; Coates [50]; Gardiner et al. [35]; Hurley et al. [16]; Xu & Gao [29]; Xu et al. [17, 27]; Xu & Zhao [28]; and Giles et al. [1].

**170. Parasphenoid:**

**(0)** absent;

**(1)** present.

From Gardiner [3]; Brazeau [9]; Davis et al. [12]; Zhu et al. [13]; and Giles et al. [1, 15].

**171. Parasphenoid:**

**(0)** terminates at/anterior to ventral otic fissure;

**(1)** extends across ventral otic fissure;

**(2)** extends to basioccipital.

From Coates [50]; Zhu & Yu [6]; Gardiner et al. [36]; Friedman [8], Xu & Gao [29]; Xu et al. [17, 27]; Xu & Zhao [28]; and Giles et al. [1].

**172. Ascending process of the parasphenoid:**

**(0)** absent;

**(1)** present.

From Patterson [51]; Coates [50]; Dietze [47]; Schultze & Cumbaa [20]; Zhu & Schultze [4]; Cloutier & Arratia [21]; Gardiner et al. [36]; Friedman & Blom [22]; Zhu et al. [7, 10, 13]; Choo [25]; Xu & Gao [29]; Xu et al. [17]; and Giles et al. [1, 15].

**173. Parasphenoid with multifid anterior margin:**

**(0)** absent;

**(1)** present.

From Friedman & Blom [22]; Friedman [8]; Zhu et al. [10, 13]; Choo [25]; and Giles et al. [1, 15].

**174. Buccohypophyseal canal pierces parasphenoid:**

**(0)** present;

**(1)** absent.

From Giles et al. [1, 15].

**175. Parasphenoid teeth:**

**(0)** small;

**(1)** large;

**(2)** absent.

From Arratia [54]; and Giles et al. [1].

**176. Parasphenoid pierced by internal carotid artery:**

**(0)** absent;

**(1)** present.

From Gardiner et al. [35]; Hurley et al. [16]; Xu & Wu [38]; Xu et al. [27]; Xu & Zhao [28]; and Giles et al. [1].

**177. Parasphenoid pierced by efferent pseudobranchial artery:**

**(0)** absent;

**(1)** present.

From Gardiner et al. [35]; Hurley et al. [16]; Xu & Wu [38]; Xu et al. [27]; Xu & Zhao [28]; and Giles et al. [1].

**178. Aortic notch in parasphenoid:**

**(0)** absent;

**(1)** present.

From Gardiner et al. [36]; and Giles et al. [1].

**179. Parabasal canal:**

**(0)** present;

**(1)** absent.

From Xu and Gao [29]; Xu et al. [17]; and Giles et al. [1].

**180. Anterolaterally divergent olfactory tracts:**

**(0)** absent;

**(1)** present.

From Coates [50]; Giles & Friedman [62]; and Giles et al. [1].

**181. Elongate olfactory tract(s):**

**(0)** absent;

**(1)** present.

From Brazeau [9]; Friedman & Brazeau [11]; Davis et al. [12]; Zhu et al. [13]; Brazeau & Friedman [14]; Giles & Friedman [62]; and Giles et al. [1, 15].

**182. Olfactory nerves carried in a single tract:**

**(0)** present;

**(1)** absent.

From Coates [50]; Giles & Friedman [62]; and Giles et al. [1].

**183. Hypophyseal chamber:**

**(0)** projects posteroventrally;

**(1)** projects ventrally or anteroventrally.

From Coates [50]; Xu & Gao [29]; Xu et al. [17]; and Giles et al. [1].

**184. Optic lobes:**

**(0)** narrower than cerebellum;

**(1)** same width or wider than cerebellum.

From Giles & Friedman [62]; and Giles et al. [1].

**185. Optic lobes:**

**(0)** smaller than telencephalon;

**(1)** larger than telencephalon.

From Coates [50]; Hurley et al. [16]; Xu et al. [17]; and Giles et al. [1].

**186. Optic tectum divided into bilateral halves:**

**(0)** absent;

**(1)** present.

From Coates [50]; and Giles et al. [1].

**187. Cerebellar corpus:**

**(0)** absent;

**(1)** present.

From Giles et al. [1, 15].

**188. Cerebellar corpus:**

**(0)** divided bilaterally;

**(1)** undivided.

From Coates [50]; Hurley et al. [16]; Xu & Gao [29]; Xu et al. [17]; and Giles et al. [1].

**189. Position of cerebellar corpus:**

**(0)** enters fourth ventricle;

**(1)** arches above fourth ventricle.

From Coates [50]; Hurley et al. [16]; Xu & Gao [29]; Xu et al. [17]; and Giles et al. [1].

**190. Cerebellar corpus with median anteriorly projecting portion:**

**(0)** absent;

**(1)** present.

From Coates [50]; Hurley et al. [16]; Xu & Gao [29]; Xu et al. [17]; and Giles et al. [1].

**191. Horizontal semicircular canal:**

**(0)** joins vestibular region dorsal to ampulla for the posterior semicircular canal;

**(1)** joins vestibular region level with ampulla for the posterior semicircular canal.

From Davis et al. [12]; Zhu et al. [13]; Giles & Friedman [62]; and Giles et al. [1, 15].

**192. Junction between ampulla of posterior semicircular canal and cranial cavity:**

**(0)** separated by short length of canal;

**(1)** confluent.

From Giles et al. [1, 15].

**193. Crus commune of anterior and posterior semicircular canal:**

**(0)** dorsal to endocranial roof;

**(1)** ventral to endocranial roof.

From Giles & Friedman [62]; and Giles et al. [1].

**194. Lateral cranial canal:**

**(0)** absent;

**(1)** present.

From Gardiner [3]; Gardiner & Schaeffer [34]; Coates [50]; Cloutier & Arratia [21]; Gardiner et al. [36]; Zhu et al. [7, 10, 13]; Giles & Friedman [62]; Xu et al. [17]; and Giles et al. [1, 15].

**195. Lateral cranial canal connects to cranial cavity anteriorly:**

**(0)** absent;

**(1)** present.

From Giles et al. [1].

**196. Enameloid on dermal bones and scales:**

**(0)** absent;

**(1)** present.

From Giles et al. [1].

**197. Extensive pore-canal network:**

**(0)** absent;

**(1)** present.

From Giles et al. [1].

**198. Enamel:**

**(0)** single-layered;

**(1)** multi-layered.

From Giles et al. [1].

**199. Enamel layers:**

**(0)** applied directly to one another;

**(1)** separated by layers of dentine.

From Giles et al. [1].

**200. Scales on body:**

**(0)** present;

**(1)** absent.

From Giles et al. [1].

**201. Scales:**

**(0)** micromeric;

**(1)** macromeric.

From Cloutier & Arratia [21]; Friedman & Blom [22]; Long et al. [23]; Swartz [24]; Zhu et al. [10]; Choo [25]; and Giles et al. [1].

**202. Scales with ‘peg-and-socket articulation':**

**(0)** absent;

**(1)** present.

From Maisey [55]; Gardiner & Schaeffer [34]; Cloutier & Ahlberg [18]; Coates [50]; Dietze [47]; Poplin & Lund [44]; Schultze & Cumbaa [20]; Cloutier & Arratia [21]; Friedman & Blom [22]; Friedman [8]; Long et al. [23]; Brazeau [9]; Swartz [24]; Zhu et al. [10, 13]; Friedman & Brazeau [11]; Lopez-Arbarello [37]; Xu & Gao [29]; Choo [25]; Davis et al. [12]; Xu et al. [17]; Xu & Zhao [28]; and Giles et al. [1, 15].

**203. Peg on rhomboid scale:**

**(0)** narrow;

**(1)** broad.

From Patterson [51]; Cloutier & Ahlberg [18]; Dietze [47]; Schultze & Cumbaa [20]; Zhu & Schultze [4]; Zhu et al. [5, 7, 10]; Zhu & Yu [6]; Cloutier & Arratia [21]; Friedman & Blom [22]; Friedman [8]; and Giles et al. [1].

**204. Anterodorsal process on scale:**

**(0)** absent;

**(1)** present.

From Patterson [51]; Gardiner [3]; Gardiner & Schaeffer [34]; Schultze & Cumbaa [20]; Zhu & Schultze [4]; Zhu et al. [5, 7, 10, 13]; Zhu & Yu [6]; Cloutier & Arratia [21]; Friedman & Blom [22]; Friedman [8]; Long et al. [23]; Swartz [24]; Choo [25]; and Giles et al. [1, 15].

**205. Scales with well-developed pores on surface:**

**(0)** absent;

**(1)** present.

From Friedman & Blom [22]; Long et al. [23]; Swartz [24]; Choo [25]; Xu et al. [17]; and Giles et al. [1].

**206. Small scales below dorsal fin:**

**(0)** absent;

**(1)** present.

From Giles et al. [1].

**207. Lepidotrichia:**

**(0)** absent;

**(1)** present.

From Friedman [8]; Brazeau [9]; Zhu et al. [10, 13]; Friedman & Brazeau [11]; Davis et al. [12]; Brazeau & Friedman [14]; and Giles et al. [1, 15].

**208. Fringing fulcra:**

**(0)** absent;

**(1)** present.

From Patterson [51]; Gardiner & Schaeffer [34]; Coates [50]; Dietze [47]; Schultze & Cumbaa [20]; Cloutier & Arratia [21]; Friedman & Blom [22]; Friedman [8]; Long et al. [23]; Swartz [24]; Zhu et al. [10, 13]; Choo [25]; Xu & Gao [29]; Xu et al. [17, 27]; Xu & Zhao [28]; and Giles et al. [1].

**209. Double headed hyomandibular:**

**(0)** absent;

**(1)** present.

From Cloutier & Ahlberg [18]; Zhu & Schultze [4]; Schultze & Cumbaa [20]; Zhu et al. [5, 7, 10, 13]; Zhu & Yu [6]; Friedman [8]; Friedman & Brazeau [11]; and Giles et al. [1, 15].

**210. Perforate hyomandibula:**

**(0)** absent;

**(1)** present.

From Friedman [8]; Zhu et al. [10, 13]; Friedman & Brazeau [11]; Xu & Gao [29]; Brazeau & Friedman [14]; Xu et al. [17, 27]; Xu & Zhao [28]; and Giles et al. [1].

**211. Opercular process:**

**(0)** absent;

**(1)** present.

From Gardiner & Schaeffer [34]; and Giles et al. [1].

**212. Ceratohyal:**

**(0)** single ossification;

**(1)** two ossifications.

From Gardiner et al. [36]; Xu & Gao [29]; Xu et al. [17]; and Giles et al. [1].

**213. Anterior ossification of ceratohyal:**

**(0)** no medial constriction;

**(1)** medial constriction (hourglass-shaped).

From Coates [50]; and Giles et al. [1].

**214. Anterior ceratohyal:**

**(0)** no groove;

**(1)** groove for afferent hyoidean artery.

From Coates [50]; and Giles et al. [1].

**215. Interhyal:**

**(0)** absent;

**(1)** present.

From Davis et al. [12]; Zhu et al. [13]; and Giles et al. [1, 15].

**216. Symplectic:**

**(0)** absent;

**(1)** present.

From Gardiner [3]; Gardiner & Schaeffer [34]; Coates [50]; Hurley et al. [16]; Xu & Zhao [28]; and Giles et al. [1].

**217. Symplectic shape:**

**(0)** tube/splint like;

**(1)** hatchet;

**(2)** L-shaped.

From Giles et al. [1].

**218. Hypohyal:**

**(0)** absent;

**(1)** present.

From Friedman & Brazeau [11]; Brazeau & Friedman [14]; and Giles et al. [1, 15].

**219. Basihyal:**

**(0)** absent;

**(1)** present.

From Davis et al. [12]; Zhu et al. [13]; and Giles et al. [1, 15].

**220. Gill arches:**

**(0)** largely restricted to area under braincase;

**(1)** extend far posterior to braincase.

From Giles et al. [1, 15].

**221. Number of ceratobranchials:**

**(0)** five;

**(1)** four.

From Giles et al. [1].

**222. Number of hypobranchials:**

**(0)** three;

**(1)** four.

From Grande [32]; Xu & Wu [38]; Xu et al. [17, 27]; Xu & Zhao [28]; and Giles et al. [1].

**223. Uncinate processes on epibranchials:**

**(0)** absent;

**(1)** present.

From Coates [50]; Xu & Gao [29]; Xu et al. [17, 27]; Xu & Zhao [28]; and Giles et al. [1].

**224. Endoskeletal urohyal:**

**(0)** absent;

**(1)** present.

From Friedman [8]; Friedman & Brazeau [11]; and Giles et al. [1, 15].

**225. Urohyal formed as a tendon bone of the sternohyoideus muscle:**

**(0)** absent;

**(1)** present.

From Arratia [54]; and Giles et al. [1].

**226. Presupracleithrum:**

**(0)** absent;

**(1)** present.

From Patterson [51]; Gardiner [3]; Gardiner & Schaeffer [34]; Taverne [19]; Lund [39]; Schultze & Cumbaa [20]; Zhu & Schultze [4]; Zhu et al. [5, 7, 10, 13]; Lund & Poplin [40]; Zhu & Yu [6]; Cloutier & Arratia [21]; Gardiner et al. [36]; Friedman & Blom [22]; Friedman [8]; Long et al. [23]; Swartz [24]; Choo [25]; Xu & Gao [29]; Xu et al. [17]; and Giles et al. [1].

**227. Presupracleithrum:**

**(0)** single;

**(1)** multiple.

From Xu et al. [17]; and Giles et al. [1].

**228. Dorsal margin of cleithrum:**

**(0)** pointed;

**(1)** broad and rounded.

From Cloutier & Ahlberg [18]; Schultze & Cumbaa [20]; Zhu & Schultze [4]; Zhu et al. [5, 7, 10]; Zhu & Yu [6]; Cloutier & Arratia [21]; Friedman [8]; and Giles et al. [1, 15].

**229. Medial wing on cleithrum:**

**(0)** absent;

**(1)** present.

From Cavin & Suteethorn [31]; and Giles et al. [1].

**230. Anocleithrum:**

**(0)** bone developed as postcleithrum;

**(1)** bone developed as anocleithrum sensu stricto;

**(2)** bone absent.

From Gardiner & Schaeffer [34]; Lund et al. [41]; Cloutier & Ahlberg [18]; Dietze [47]; Poplin & Lund [44]; Schultze & Cumbaa [20]; Zhu & Schultze [4]; Zhu et al. [5, 7, 10, 13]; Zhu & Yu [6]; Cloutier & Arratia [21]; Friedman [8]; and Giles et al. [1].

**231. Clavicle:**

**(0)** present as a broad plate;

**(1)** much reduced or absent.

From Coates, 1999; Xu & Gao, 2011; Xu et al., 2014; Xu & Zhao, 2016; and Giles et al., 2017.

**232. Serrated organ:**

**(0)** absent;

**(1)** present.

From Arratia, 2013; and Giles et al., 2017.

**233. Interclavicle:**

**(0)** present;

**(1)** absent.

From Cloutier & Arratia, 2004; Xu et al., 2014; and Giles et al., 2017.

**234. Triradiate scapulocoracoid:**

**(0)** absent;

**(1)** present.

From Zhu & Schultze, 2001; Zhu et al., 2001; Zhu & Yu, 2002; Zhu et al., 2006; Friedman, 2007; Zhu et al., 2009; Xu & Gao, 2011; Zhu et al., 2013; Xu et al., 2014; and Giles et al., 2017.

**235. Perforate propterygium:**

**(0)** absent;

**(1)** present.

From Patterson, 1982; Gardiner, 1984; Gardiner & Schaeffer, 1989; Taverne, 1997; Coates, 1999; Zhu & Schultze, 2001; Zhu et al., 2001; Zhu & Yu, 2002; Zhu et al., 2006; Brazeau, 2009; Zhu et al., 2009; Friedman & Brazeau, 2010; Xu & Gao, 2011; Davis et al., 2012; Zhu et al., 2013; Xu et al., 2014; and Giles et al. [1, 15].

**236. Anterior rays embrace propterygium:**

**(0)** absent;

**(1)** present;

**(2)** fused.

From Patterson, 1982; Gardiner, 1984; Gardiner & Schaeffer, 1989; Taverne, 1997; Coates, 1999; Schultze & Cumbaa, 2001; Zhu & Schultze, 2001; Friedman & Blom, 2006; Long et al., 2008; Swartz, 2009; Choo, 2011; Xu & Gao, 2011; and Giles et al., 2017.

**237. Propterygium fused to first ray:**

**(0)** absent;

**(1)** present.

From Giles et al., 2017.

**238. Pectoral fin endoskeleton:**

**(0)** extends far beyond body wall (fins lobate);

**(1)** barely extends beyond body wall (fins not lobate).

From Taverne, 1997; Coates, 1999; Friedman & Blom, 2006; Long et al., 2008; Swartz, 2009; Xu & Gao, 2011; Xu et al., 2014; and Giles et al., 2017.

**239. Pectoral fin radials:**

**(0)** unjointed;

**(1)** jointed.

From Zhu & Yu, 2002; Friedman, 2007; and Giles et al., 2017.

**240. Fin articulation:**

**(0)** monobasal;

**(1)** polybasal.

From Zhu & Schultze, 2001; Zhu et al., 2001; Zhu & Yu, 2002; Zhu et al., 2006; Friedman, 2007; Zhu et al., 2009; Friedman & Brazeau, 2010; Zhu et al., 2013; and Giles et al. [1, 15].

**241. Pectoral fin-ray segmentation:**

**(0)** roughly even segmentation to fin base;

**(1)** proximal segments elongate with terminal segmentation;

**(2)** no significant segmentation on pectoral fin;

**(3)** terminal segments elongate with proximal segmentation.

From Coates, 1999; Friedman & Blom, 2006; Long et al., 2008; Choo, 2011; Xu & Gao, 2011; Xu et al., 2014; and Giles et al., 2017.

**242. Pectoral fin:**

**(0)** leaf-like;

**(1)** not leaf-like.

From Giles et al., 2017.

**243. Paired fin spines:**

**(0)** absent;

**(1)** present.

From Zhu et al., 2001; Zhu & Yu, 2002; Friedman, 2007; Zhu et al., 2009; Brazeau, 2009; Davis et al., 2012; Zhu et al., 2013; and Giles et al. [1, 15].

**244. Pelvic fins:**

**(0)** absent;

**(1)** present.

From Friedman & Blom, 2006; Friedman, 2007; Brazeau, 2009; Choo, 2011; Davis et al., 2012; Zhu et al., 2013; Brazeau & Friedman, 2014; and Giles et al. [1, 15].

**245. Pelvic fin insertion:**

**(0)** shorter than fin depth (short based);

**(1)** longer than fin depth (long based).

From Gardiner & Schaeffer, 1989; Coates, 1998; Coates, 1999; Lund, 2000; Schultze & Cumbaa, 2001; Cloutier & Arratia, 2004; Friedman & Blom, 2006; Zhu et al., 2006; Long et al., 2008; Swartz, 2009; Zhu et al., 2009; Choo, 2011; Xu et al., 2014; and Giles et al., 2017.

**246. Basal fulcra on fins:**

**(0)** absent;

**(1)** present.

From Friedman, 2007; Zhu & Yu, 2002; and Giles et al., 2017.

**247. Dorsal scutes anterior to dorsal fin:**

**(0)** absent;

**(1)** few limited to region immediately anterior to fin (basal fulcra only);

**(2)** many, extending to posterior of skull roof (complete set of dorsal ridge scutes).

From Lund, 2000; Poplin & Lund, 2000; Cloutier & Arratia, 2004; Friedman & Blom, 2006; Long et al., 2008; Swartz, 2009; Choo; 2011; and Giles et al., 2017.

**248. Ventral scutes between hypochordal lobe of caudal fin and anal fin:**

**(0)** absent;

**(1)** present.

From Patterson, 1982; Taverne, 1997; Friedman & Blom, 2006; Long et al., 2008; Choo, 2011; and Giles et al., 2017.

**249. Ventral scutes anterior to anal fin:**

**(0)** absent;

**(1)** present.

From Cloutier & Arratia, 2004; Friedman & Blom, 2006; Long et al., 2008; Swartz, 2009; Choo, 2011; and Giles et al., 2017.

**250. Dorsal fin(s):**

**(0)** two;

**(1)** one.

From Gardiner & Schaeffer, 1989; Schultze & Cumbaa, 2001; Zhu & Schultze, 2001; Zhu et al., 2001; Zhu & Yu, 2002; Cloutier & Arratia, 2004; Friedman & Blom, 2006; Zhu et al., 2006; Friedman, 2007; Long et al., 2008; Brazeau, 2009; Swartz, 2009; Zhu et al., 2009; Choo, 2011; Davis et al., 2012; Zhu et al., 2013; and Giles et al. [1, 15].

**251. Relative positions of anal and (second) dorsal fin:**

**(0)** anal shifted anteriorly relative to dorsal;

**(1)** ins opposite one another;

**(2)** anal shifted posteriorly relative to dorsal.

From Poplin & Lund, 2000; Cloutier & Arratia, 2004; Friedman & Blom, 2006; Long et al., 2008; Swartz, 2009; Choo, 2011; and Giles et al., 2017.

**252. Median fins (except caudal fin):**

**(0)** rays more numerous than radials;

**(1)** rays and radials equal.

From Gardiner et al., 2005; Xu & Gao, 2011; Xu et al., 2014, 2015; Xu & Zhao, 2016; and Giles et al., 2017.

**253. Proximal and middle radials of dorsal fin:**

**(0)** proximal and middle radials of similar size;

**(1)** proximal radials substantially enlarged.

From Giles et al., 2017.

**254. Posteriormost proximal radial of dorsal fin:**

**(0)** enlarged plate;

**(1)** smaller than more anterior radials.

From Giles et al., 2017.

**255. Epichordal lobe of caudal fin:**

**(0)** present;

**(1)** absent.

From Patterson, 1982; Cloutier & Ahlberg, 1996; Coates, 1999; Schultze & Cumbaa, 2001; Zhu & Schultze, 2001; Friedman & Blom, 2006; Long et al., 2008; Swartz, 2009; Choo, 2011; and Giles et al., 2017.

**256. Fulcra along dorsal ridge of caudal fin:**

**(0)** absent;

**(1)** present.

From Patterson, 1982; Taverne, 1997; Gardiner & Schaeffer, 1989; Gardiner et al., 2005; Friedman & Blom, 2006; Long et al., 2008; Choo, 2011; and Giles et al., 2017.

**257. Caudal fin geometry:**

**(0)** long chordal lobe;

**(1)** short chordal lobe.

From Gardiner et al., 2005; and Giles et al., 2017.

**258. Posterior margin of caudal fin:**

**(0)** forked;

**(1)** unforked.

From Xu & Gao, 2011; Xu et al., 2014; Xu & Zhao, 2016; and Giles et al., 2017.

**259. Diplospondyly in mid-caudal region:**

**(0)** absent;

**(1)** present.

From Arratia, 2013; and Giles et al., 2017.

**260. Median neural spines in caudal region:**

**(0)** absent;

**(1)** present.

From Coates, 1999; Hurley et al., 2007; Xu et al., 2014; and Giles et al., 2017.

**261. Uroneural:**

**(0)** absent;

**(1)** present.

From Pinna, 1996; Hurley et al., 2007; Xu & Wu, 2012, Xu et al., 2014; Xu & Zhao, 2016; and Giles et al., 2017.

**262. Division of hypurals into dorsal and ventral groups:**

**(0)** absent;

**(1)** present.

From Pinna, 1996, Xu & Wu, 2012, Xu et al., 2014; Xu & Zhao, 2016; and Giles et al., 2017.

**263. Number of caudal lepidotrichs borne per hypural:**

**(0)** multiple;

**(1)** single.

From Giles et al., 2017.

**264. Opistocoelous vertebrae:**

**(0)** absent;

**(1)** present.

From Wiley, 1976; Lopez-Arbarello, 2011; and Giles et al., 2017.

**265. Ossified ribs:**

**(0)** present;

**(1)** absent.

From Giles et al., 2017.

1. **Character states that support the nodes for the tree presented in Figure S1.** The asteroid (*) mark in the node descriptions indicates a uniquely derived feature.

**Figure S1. Phylogenetic tree with nodes labels.** Same tree as shown in Fig. 16 of the main text. Labelled nodes support are described below.

Node 1: Ch. 1 (1*); Ch. 145 (1); Ch. 191 (1); Ch. 215 (1); and Ch. 220 (0).

Node 2: Ch. 20 (1); Ch. 28 (1); Ch. 42 (1); Ch. 44 (0); Ch. 45 (1); Ch. 56 (1); Ch. 63 (1); Ch. 66 (1); Ch. 78 (1*); Ch. 89 (1); Ch. 99 (1); Ch. 185 (1); Ch. 194 (1); Ch. 199 (0*); Ch. 208 (1); Ch. 214 (1); Ch. 219 (1); Ch. 220 (1); Ch. 250 (1); Ch. 256 (1); and Ch. 258 (0).

Node 3: Ch. 13 (1); Ch. 14 (1); Ch. 17 (1); Ch. 19 (1); Ch. 23 (0); Ch. 86 (1); Ch. 110 (1); Ch. 127 (1); Ch. 151 (1); Ch. 172 (1); Ch. 197 (0); Ch. 234 (1); Ch. 235 (1); Ch. 241 (1); and Ch. 255 (1).

Node 4: Ch. 21 (1); Ch. 28 (2); Ch. 44 (1); Ch. 46 (1); Ch. 210 (1); and Ch. 238 (1).

Node 5: Ch. 36 (1); Ch. 56 (0); Ch. 65 (1); Ch. 70 (0); Ch. 91 (3); Ch. 99 (0); Ch. 121 (1); Ch. 136 (2); Ch. 138 (2); Ch. 141 (1); Ch. 143 (1); Ch. 146 (1); Ch. 180 (0); Ch. 184 (1); Ch. 186 (1); Ch. 187 (1*); Ch. 193 (1); Ch. 211 (1); and Ch. 233 (1).

Node 6: Ch. 3 (1); Ch. 7 (0); Ch. 21 (0); Ch. 66 (0); Ch. 84 (2); Ch. 139 (1); Ch. 148 (0); Ch. 151 (2); Ch. 171 (1); Ch. 219 (0); Ch. 222 (0); Ch. 223 (1); Ch. 235 (0); Ch. 238 (0); Ch. 253 (1); and Ch. 257 (1).

Node 7: Ch. 6 (1); Ch. 69 (0); Ch. 99 (2); Ch. 115 (1); Ch. 174 (1); Ch. 175 (2); Ch. 247 (0); Ch. 248 (0); and Ch. 249 (0).

Node 8: Ch. 3 (0); Ch. 27 (1); Ch. 100 (1); Ch. 103 (1); Ch. 107 (1); Ch. 155 (1); Ch. 156 (1); Ch. 178 (1); Ch. 180 (1); Ch. 181 (0); Ch. 185 (0); Ch. 191 (0); Ch. 214 (0); Ch. 234 (0); Ch. 251 (2); and Ch. 257 (0).

Node 9: Ch. 8 (1); Ch. 12 (1); Ch. 71 (1); Ch. 80 (1); Ch. 89 (2); Ch. 94 (2); Ch. 105 (1); Ch. 139 (0); Ch. 145 (0); Ch. 152 (1); Ch. 167 (1); Ch. 188 (1); Ch. 190 (1*); Ch. 213 (1); and Ch. 249 (1).

Node 10: Ch. 42 (0); Ch. 68 (0); Ch. 120 (1); Ch. 123 (1); Ch. 142 (1); Ch. 151 (0); Ch. 153 (1); and Ch. 238 (1).

Node 11: Ch. (); Ch. 3 (1); Ch. 13 (0); Ch. 21 (1); Ch. 43 (1); Ch. 63 (0); Ch. 99 (0); Ch. 122 (0); Ch. 159 (1*); Ch. 164 (1); Ch. 171 (2); Ch. 179 (1); Ch. 184 (0); Ch. 186 (0); Ch. 223 (0); Ch. 227 (1); Ch. 234 (1); Ch. 241 (0); and Ch. 253 (0).

Node 12: Ch. 14 (0); Ch. 17 (0); Ch. 19 (0); Ch. 32 (1); Ch. 45 (0); Ch. 55 (1); Ch. 58 (1); Ch. 67 (0); Ch. 90 (1); Ch. 102 (1*); Ch. 110 (2); Ch. 114 (2); Ch. 135 (2); Ch. 136 (0); Ch. 141 (0); Ch. 142 (0); Ch. 167 (0); Ch. 173 (1); Ch. 200 (1); Ch. 208 (0); Ch. 210 (0); Ch. 211 (0); and Ch. 233 (0).

Node 13: Ch. 120 (0); Ch. 156 (0); Ch. 157 (1); Ch. 174 (0); and Ch. 238 (0).

Node 14: Ch.6 (0); Ch. 7 (1); Ch. 44 (0); Ch. 51 (2); Ch. 72 (1); Ch. 110 (0); Ch. 111 (1); Ch. 114 (1); Ch. 231 (1); and Ch. 247 (1).

Node 15: Ch. 226 (1); Ch. 230 (2); Ch. 241 (2); Ch. 247 (2); and Ch. 258 (1).

Node 16: Ch. 54 (2); Ch. 58 (2); Ch. 80 (0); Ch. 93 (1); Ch. 108 (1); Ch. 135 (1); Ch. 169 (1); Ch. 178 (0); Ch. 180 (0); Ch. 183 (1); Ch. 189 (1*); Ch. 216 (1); Ch. 230 (2); Ch. 231 (1); Ch. 252 (1); Ch. 254 (1); and Ch. 265 (0).

Node 17: Ch. 8 (0); Ch. 11 (1); Ch. 19 (0); Ch. 51 (0); Ch. 52 (1); Ch. 71 (0); and Ch. 257 (1).

Node 18: Ch. 49 (1); Ch. 54 (0); Ch. 63 (0); Ch. 64 (1); Ch. 89 (1); Ch. 212 (1); and Ch. 247 (1).

Node 19: Ch. 51 (2); Ch. 54 (3); Ch. 110 (0); Ch. 116 (1); Ch. 176 (1); and Ch. 230 (0).

Node 20: Ch. 15 (1); Ch. 20 (0); Ch. 55 (1); Ch. 72 (1); Ch. 73 (1*); Ch. 112 (1); Ch. 115 (2); and Ch. 118 (1).

Node 21: Ch. 30 (0); Ch. 33 (1); Ch. 57 (1); Ch. 75 (1); Ch. 158 (1*); Ch. 166 (1); Ch. 168 (1); and Ch. 171 (2).

Node 22: Ch. 10 (1*); Ch. 14 (0); Ch. 50 (1); Ch. 61 (1); Ch. 96 (1); Ch. 107 (0); Ch. 161 (1); Ch. 213 (0); Ch. 222 (1); Ch. 232 (1); Ch. 247 (0); and Ch. 249 (0).

Node 23: Ch. 54 (0); Ch. 57 (0); Ch. 74 (1); Ch. 119 (1); Ch. 134 (1); Ch. 175 (0); Ch. 176 (0); and Ch. 226 (1).

Node 24: Ch. 11 (2); Ch. 18 (1); Ch. 32 (1); Ch. 62 (1); Ch. 105 (0); Ch. 139 (1); Ch. 162 (1); Ch. 163 (1); Ch. 168 (0); Ch. 173 (1); and Ch. 217 (1).

Node 25: Ch. 12 (0); Ch. 30 (1); Ch. 52 (0); Ch. 88 (1); Ch. 119 (0); Ch. 157 (1); Ch. 178 (1); Ch. 226 (0); Ch. 258 (1); and Ch. 263 (1).

Node 26: Ch. 59 (1); Ch. 60 (1*); Ch. 62 (0); Ch. 74 (0); Ch. 75 (0); Ch. 96 (0); Ch. 122 (0); Ch. 134 (0); Ch. 135 (2); Ch. 139 (0); Ch. 160 (1); Ch. 162 (0); Ch. 164 (1); Ch. 213 (1); Ch. 217 (0); and Ch. 229 (1).

Node 27: Ch. 27 (0); Ch. 43 (1); Ch. 54 (3); Ch. 58 (1); Ch. 104 (1); Ch. 172 (0); Ch. 217 (2); Ch. 238 (0); Ch. 251 (1); and Ch. 264 (1*).

Node 28: Ch. 43 (2); Ch. 63 (1); Ch. 72 (0); Ch. 84 (1); Ch. 86 (0); Ch. 87 (1); Ch. 110 (1); Ch. 118 (0); and Ch. 119 (1).

Node 29: Ch. 12 (1); Ch. 30 (0); Ch. 55 (0); Ch. 88 (0); Ch. 91 (4); Ch. 103 (0); Ch. 161 (0); Ch. 168 (1); Ch. 174 (0); Ch. 219 (1); and Ch. 236 (0).

Node 30: Ch. 14 (1); Ch. 20 (1); Ch. 54 (1); and Ch. 162 (1).

Node 31: Ch. 29 (1); Ch. 46 (0); Ch. 47 (1); Ch. 59 (0); Ch. 61 (0); Ch. 69 (1); Ch. 117 (1); Ch. 169 (0); and Ch. 248 (1).

Node 32: Ch. 15 (0); Ch. 20 (1); Ch. 27 (0); Ch. 46 (0); Ch. 58 (1); Ch. 91 (4); Ch. 146 (0); Ch. 157 (1); Ch. 160 (1); Ch. 165 (1*); Ch. 178 (1); Ch. 181 (1); Ch. 185 (1); Ch. 191 (1); Ch. 195 (1); Ch. 198 (0); and Ch. 225 (1).

Node 33: Ch. 55 (0); Ch. 151 (2); Ch. 156 (0); Ch. 177 (1); Ch. 193 (0); Ch. 217 (1); Ch. 235 (1); Ch. 247 (2); and Ch. 258 (1).

Node 34: Ch. 5 (1); Ch. 18 (1); Ch. 41 (1); Ch. 43 (1); Ch. 46 (1); Ch. 62 (1); Ch. 79 (1); Ch. 100 (0); Ch. 105 (0); Ch. 110 (1); Ch. 226 (1); Ch. 241 (2); and Ch. 248 (1).

Node 35: Ch. 9 (1); Ch. 11 (0); Ch. 30 (1); Ch. 54 (1); Ch. 89 (2); Ch. 95 (1); Ch. 106 (1); Ch. 135 (0); Ch. 175 (0); Ch. 182 (0); Ch. 214 (1); Ch. 237 (1*); Ch. 261 (1); and Ch. 262 (1).

Node 36: Ch. 20 (0); Ch. 27 (1); Ch. 29 (1); Ch. 57 (0); Ch. 66 (1); Ch. 75 (2); Ch. 90 (1); Ch. 112 (0); Ch. 196 (0); Ch. 208 (0); Ch. 247 (0); and Ch. 249 (0).

Node 37: Ch. 56 (1); Ch. 110 (1); Ch. 130 (1); Ch. 157 (0); Ch. 16 (0); Ch. 177 (1); Ch. 179 (1); Ch. 224 (1); Ch. 232 (1); Ch. 238 (0); and Ch. 254 (0).

Node 38: Ch. 54 (0); Ch. 55 (0); Ch. 95 (0); Ch. 134 (1); Ch. 138 (0); Ch. 141 (0); Ch. 163 (1); Ch. 194 (0); Ch. 202 (0); and Ch. 219 (1).

Node 39: Ch. 66 (1); Ch. 85 (1*); Ch. 98 (1*); Ch. 100 (0); Ch. 110 (2); Ch. 113 (1); Ch. 174 (0); Ch. 233 (0); Ch. 247 (2); Ch. 248 (1); and Ch. 255 (0).

Node 40: Ch. 20 (0); Ch. 69 (1); Ch. 71 (0); and Ch. 115 (0).

Node 41: Ch. 44 (0); Ch. 54 (3); Ch. 58 (2); Ch. 93 (1); Ch. 115 (0); Ch. 171 (2); Ch. 210 (0); Ch. 221 (1); and Ch. 236 (0).

Node 42: Ch. 6 (0); Ch. 7 (1); Ch. 21 (1); Ch. 32 (1); Ch. 43 (1); Ch. 55 (1); Ch. 94 (0); Ch. 99 (0); Ch. 101 (1*); Ch. 121 (0); Ch. 127 (0); Ch. 128 (1*); Ch. 136 (0); Ch. 138 (1); Ch. 143 (0); Ch. 175 (0); Ch. 222 (1); Ch. 225 (1); Ch. 254 (1); Ch. 256 (0); Ch. 257 (1); Ch. 258 (1); Ch. 263 (1); and Ch. 265 (0).

Node 43: Ch. 44 (1); Ch. 89 (2); Ch. 122 (0); Ch. 206 (1); Ch. 208 (0); and Ch. 223 (0).

Node 44: Ch. 6 (1); Ch. 20 (0); Ch. 58 (0); Ch. 70 (1); Ch. 109 (0); Ch. 110 (0); Ch. 112 (1); and Ch. 206 (0).

Node 45: Ch. 27 (1); Ch. 38 (1); Ch. 43 (1); Ch. 55 (1); Ch. 129(1*); Ch. 130 (1); Ch. 134 (1); Ch. 135 (1); Ch. 146 (0); Ch. 149 (1); Ch. 152 (1); Ch. 155 (1); Ch. 182 (0); Ch. 213 (1); Ch. 239 (1); Ch. 251 (2); and Ch. 260 (0).

Node 46: Ch. 39 (1); Ch. 42 (0); Ch. 54 (1); Ch. 66 (0); Ch. 76 (1); Ch. 99 (2); Ch. 119 (1); and Ch. 174 (1).

Node 47: Ch. 7 (0); Ch. 49 (1); Ch. 51 (2); Ch. 54 (2); Ch. 135 (0); Ch. 150 (1*); Ch. 188 (1); Ch. 249 (0); and Ch. 255 (0).

Node 48: Ch. 149 (0); Ch. 151 (2); and Ch. 183 (1).

Node 49: Ch. 12 (1); Ch. 91 (2); Ch. 94 (2); Ch. 146 (1); Ch. 180 (1); Ch. 182 (1); Ch. 226 (1); Ch. 241 (0); and Ch. 249 (1).

Node 50: Ch. 76 (0); and Ch. 89 (2).

Node 51: Ch. 36 (0); Ch. 51 (1); Ch. 56 (1); Ch. 57 (1); and Ch. 141 (0).

Node 52: Ch. 4 (1); Ch. 42 (1); Ch. 54 (1); and Ch. 84 (2).

Node 53: Ch. 3 (1); Ch. 54 (2); Ch. 110 (0); and Ch. 248 (0).

Node 54: Ch. 6 (1); Ch. 28 (0); Ch. 29 (1); Ch. 55 (0); Ch. 58 (2); Ch. 110 (0); Ch. 113 (1); Ch. 211 (0); and Ch. 238 (0).

Node 55: Ch. 43 (0); and Ch. 45 (0).

Node 56: Ch. 7 (1); Ch. 42 (1); Ch. 109 (1); and Ch. 119(0).

Node 57: Ch. 5 (1); Ch. 8 (1); Ch. 27 (0); Ch. 33 (1); Ch. 45 (0); Ch. 50 (1); Ch. 62 (1); Ch. 70 (1); Ch. 210 (0); Ch. 212 (1); Ch. 230 (2); Ch. 238 (0); and Ch. 247 (0).

Node 58: Ch. 7 (1); Ch. 18 (1); Ch. 42 (1); Ch. 44 (0); Ch. 49 (0); Ch. 104 (1); Ch. 106 (1); Ch. 115 (1); Ch. 135 (1); and Ch. 153 (1).

Node 59: Ch. 25 (0); Ch. 52 (1); Ch. 112 (1); Ch. 173 (1); Ch. 192 (0); Ch. 226 (1); and Ch. 247 (2).

Node 60: Ch. 4 (1); Ch. 38 (1); Ch. 88 (1); Ch. 110 (0); Ch. 151 (0); Ch. 245 (1); Ch. 247 (1); and Ch. 255 (0).

Node 61: Ch. 33 (1); Ch. 40 (1*); Ch. 52 (0); Ch. 89 (2); Ch. 151 (2); and Ch. 172 (0).

Node 62: Ch. 13 (0); Ch. 28 (1); Ch. 233 (1); and Ch. 251 (2).

Node 63: Ch. 43 (1); Ch. 53 (1); Ch. 134 (1); Ch. 136 (1); and Ch. 205 (1).

Node 64: Ch. 44 (0); Ch. 47 (1); and Ch. 119 (1).

Node 65: Ch. 22 (1); Ch. 43 (1); Ch. 50 (1); Ch. 88 (1); Ch. 249 (0); and Ch. 265 (0).

Node 66: Ch. 53 (1); Ch. 76 (1); Ch. 109 (1); Ch. 201 (0); Ch. 245 (1); and Ch. 251 (0).

Node 67: Ch. 7 (0); Ch. 20 (0); Ch. 46 (1); Ch. 202 (0); Ch. 204 (0); Ch. 236 (0); Ch. 239 (1); Ch. 248 (0); and Ch. 258 (1).

Node 68: Ch. 7 (0); Ch. 24 (1*); Ch. 34 (0); Ch. 37 (1); Ch. 48 (1); Ch. 58 (1); Ch. 61 (1); Ch. 65 (1); Ch. 82 (1); Ch. 83 (0); Ch. 94 (3); Ch. 96 (1); Ch. 126 (1); Ch. 131 (1*); Ch. 137 (1*); Ch. 183 (1); Ch. 203 (1); Ch. 224 (1); Ch. 226 (1); Ch. 230 (1); and Ch. 233 (1).

Node 69: Ch. 16 (1*); Ch. 35 (0); Ch. 48 (0); Ch. 49 (1); Ch. 61 (0); Ch. 79 (1); Ch. 82 (0); Ch. 83 (1); Ch. 89 (1); Ch. 92 (1*); Ch. 100 (1); Ch. 110 (0); Ch. 145 (0); Ch. 148 (0); Ch. 216 (1); and Ch. 228 (0).

Node 70: Ch. 18 (1); Ch. 67 (0); Ch. 93 (1); Ch. 124 (0); Ch. 126 (0); and Ch. 197 (0).

Node 71: Ch. 5 (1); Ch. 22 (1); Ch. 26 (1); Ch. 32 (1); Ch. 37 (0); Ch. 58 (0); Ch. 122 (1); Ch. 140 (1); Ch. 147 (0); Ch. 198 (0); and Ch. 233 (0).

Node 72: Ch. 25 (0); Ch. 97 (1); Ch. 119 (1); Ch. 125 (1*); Ch. 126 (0); Ch. 179 (1); Ch. 202 (0); Ch. 212 (1); Ch. 214 (1); Ch. 246 (1); and Ch. 251 (2).

Node 73: Ch. 82 (0); Ch. 83 (1); Ch. 84 (1); Ch. 196 (0); and Ch. 197 (0).

Node 74: Ch. 31 (1); Ch. 57 (0); Ch. 87 (1); Ch. 221 (1); Ch. 234 (0); Ch. 239 (1); and Ch. 242 (0).

Node 75: Ch. 4 (1); Ch. 25 (0); Ch. 47 (1); Ch. 81 (1); Ch. 87 (1); Ch. 91 (0); Ch. 112 (1); Ch. 132 (1); and Ch. 243 (1).

Node 76: Ch. 82 (1); Ch. 139 (1); and Ch. 222 (0).

**References**

1. Giles S, Xu GH, Near TJ, Friedman M. Early members of ‘living fossil’lineage imply later origin of modern ray-finned fishes. Nature. 2017 Sep 14;549(7671):265-268.
2. Forey PL. *Latimeria*: a paradoxical fish. Proceedings of the Royal Society of London. Series B. Biological Sciences. 1980 Jul 17;208(1172):369-84.
3. Gardiner BG. The relationships of the palaeoniscid fishes, a review based on new specimens of Mimia and Moythomasia from the Upper Devonian of Western Australia. Bulletin of the British Museum (Natural History), Geology Series. 1984;37(4):173-428.
4. Zhu M, Schultze HP. Interrelationships of basal osteichthyans. Systematics Association Special Volume. 2001 Feb 15;61:289-314.
5. Zhu M, Yu X, Ahlberg PE. A primitive sarcopterygian fish with an eyestalk. Nature. 2001 Mar 1;410(6824):81-4.
6. Zhu M, Yu X. A primitive fish close to the common ancestor of tetrapods and lungfish. Nature. 2002 Aug 15;418(6899):767-70.
7. Zhu M, Yu X, Wang W, Zhao W, Jia L. A primitive fish provides key characters bearing on deep osteichthyan phylogeny. Nature. 2006 May 4;441(7089):77-80.
8. Friedman M. *Styloichthys* as the oldest coelacanth: implications for early osteichthyan interrelationships. Journal of Systematic Palaeontology. 2007 Jan 1;5(3):289-343.
9. Brazeau MD. The braincase and jaws of a Devonian ‘acanthodian’and modern gnathostome origins. Nature. 2009 Jan 15;457(7227):305-8.
10. Zhu M, Zhao W, Jia L, Lu J, Qiao T, Qu Q. The oldest articulated osteichthyan reveals mosaic gnathostome characters. Nature. 2009 Mar 26;458(7237):469-74.
11. Friedman M, Brazeau MD. A reappraisal of the origin and basal radiation of the Osteichthyes. Journal of Vertebrate Paleontology. 2010 Jan 29;30(1):36-56.
12. Davis SP, Finarelli JA, Coates MI. Acanthodes and shark-like conditions in the last common ancestor of modern gnathostomes. Nature. 2012 Jun 14;486(7402):247-50.
13. Zhu M, Yu X, Ahlberg PE, Choo B, Lu J, Qiao T, Qu Q, Zhao W, Jia L, Blom H, Zhu YA. A Silurian placoderm with osteichthyan-like marginal jaw bones. Nature. 2013 Oct 10;502(7470):188-93.
14. Brazeau MD, Friedman M. The characters of Palaeozoic jawed vertebrates. Zoological journal of the Linnean Society. 2014 Apr 1;170(4):779-821.
15. Giles S, Darras L, Clément G, Blieck A, Friedman M. An exceptionally preserved Late Devonian actinopterygian provides a new model for primitive cranial anatomy in ray-finned fishes. Proceedings of the Royal Society B: Biological Sciences. 2015 Oct 7;282(1816):20151485.
16. Hurley IA, Mueller RL, Dunn KA, Schmidt EJ, Friedman M, Ho RK, Prince VE, Yang Z, Thomas MG, Coates MI. A new time-scale for ray-finned fish evolution. Proceedings of the Royal Society B: Biological Sciences. 2007 Feb 22;274(1609):489-98.
17. Xu GH, Gao KQ, Finarelli JA. A revision of the Middle Triassic scanilepiform fish *Fukangichthys longidorsalis* from Xinjiang, China, with comments on the phylogeny of the Actinopteri. Journal of Vertebrate Paleontology. 2014 Jun 7;34(4):747-59.
18. Cloutier R, Ahlberg PE. Morphology, characters, and the interrelationships of basal sarcopterygians. Interrelationships of fishes. 1996 Jan 1:445-79.
19. Taverne L. *Osorioichthys marginis*,“Paléonisciforme” du Famennien de Belgique, et la phylogénie des Actinoptérygiens dévoniens (Pisces). Bulletin de l'institut Royal des Sciences Naturelles de Belgique. 1997;67:57-78.
20. Schultze HP, Cumbaa SL. *Dialipina* and the characters of basal osteichthyans. Major events in early vertebrate evolution. 2001:315-32.
21. Cloutier R, Arratia G. Early diversification of actinopterygians. Recent advances in the origin and early radiation of vertebrates. 2004:217-70.
22. Friedman M, Blom H. A new actinopterygian from the Famennian of East Greenland and the interrelationships of Devonian ray-finned fishes. Journal of Paleontology. 2006 Nov;80(6):1186-204.
23. Long JA, Choo B, Young GC. A new basal actinopterygian fish from the Middle Devonian Aztec Siltstone of Antarctica. Antarctic Science. 2008 Aug;20(4):393-412.
24. Swartz BA. Devonian actinopterygian phylogeny and evolution based on a redescription of *Stegotrachelus finlayi*. Zoological Journal of the Linnean Society. 2009 Aug 1;156(4):750-84.
25. Choo B. Revision of the actinopterygian genus *Mimipiscis* (= *Mimia*) from the Upper Devonian Gogo Formation of Western Australia and the interrelationships of the early Actinopterygii. Earth and Environmental Science Transactions of the Royal Society of Edinburgh. 2012 Mar;102(2):77-104.
26. Xu GH, Zhao LJ, Gao KQ, Wu FX. A new stem-neopterygian fish from the Middle Triassic of China shows the earliest over-water gliding strategy of the vertebrates. Proceedings of the Royal Society B: Biological Sciences. 2013 Jan 7;280(1750):20122261.
27. Xu GH, Gao KQ, Coates MI. Taxonomic revision of *Plesiofuro mingshuica* from the Lower Triassic of northern Gansu, China, and the relationships of early neopterygian clades. Journal of Vertebrate Paleontology. 2015 Nov 2;35(6):e1001515.
28. Xu GH, Zhao LJ. A Middle Triassic stem-neopterygian fish from China shows remarkable secondary sexual characteristics. Science Bulletin. 2016 Feb;61:338-44.
29. Xu GH, Gao KQ. A new scanilepiform from the Lower Triassic of northern Gansu Province, China, and phylogenetic relationships of non-teleostean Actinopterygii. Zoological Journal of the Linnean Society. 2011 Mar 1;161(3):595-612.
30. Arratia G. The monophyly of Teleostei and stem-group teleosts. Mesozoic fishes. 1999;2:265-334.
31. Cavin L, Suteethorn V. A new semionotiform (actinopterygii, Neopterygii) from Upper Jurassic–lower cretaceous deposits of north‐east thailand, with comments on the relationships of semionotiforms. Palaeontology. 2006 Mar;49(2):339-53.
32. Grande L. An empirical synthetic pattern study of gars (Lepisosteiformes) and closely related species, based mostly on skeletal anatomy. The resurrection of Holostei. Ichthyology & Herpetology. 2010 Oct 4;10(2A):1–871.
33. Xu GH, Shen CC. *Panxianichthys imparilis* gen. et sp. nov., a new ionoscopiform (Halecomorphi) from the Middle Triassic of Guizhou, China. Vertebrata PalAsiatica. 2015 Mar 15;53(1):1–6.
34. Gardiner BG, Schaeffer B. Interrelationships of lower actinopterygian fishes. Zoological Journal of the Linnean Society. 1989 Oct 1;97(2):135-87.
35. Gardiner BG, Maisey JG, Littlewood DT. Interrelationships of basal neopterygians. Interrelationships of Fishes. Academic Press, San Diego. 1996 Nov 8:117-46.
36. Gardiner BG, Schaeffer B, Masserie JA. A review of the lower actinopterygian phylogeny. Zoological Journal of the Linnean Society. 2005 Aug 1;144(4):511-25.
37. López-Arbarello A. Phylogenetic interrelationships of ginglymodian fishes (Actinopterygii: Neopterygii). PLoS One. 2012 Jul 11;7(7):e39370.
38. Xu G, Wu F. A deep-bodied ginglymodian fish from the Middle Triassic of eastern Yunnan Province, China, and the phylogeny of lower neopterygians. Chinese Science Bulletin. 2012 Jan;57:111-8.
39. Lund R. The new actinopterygian order Guildayichthyiformes from the Lower Carboniferous of Montana (USA). Geodiversitas. 2000;22(2): 171–206.
40. Lund R, Poplin C. Cladistic analysis of the relationships of the tarrasiids (Lower Carboniferous actinopterygians). Journal of Vertebrate Paleontology. 2002 Jan 1;22(3):480-6.
41. Lund R, Poplin C, McCarthy K. Preliminary analysis of the interrelationships of some paleozoic actinopterygii. Geobios. 1995 Jan 1;28:215-20.
42. Ahlberg PE, Johanson Z. Osteolepiforms and the ancestry of tetrapods. Nature. 1998 Oct 22;395(6704):792-4.
43. Ahlberg P, Lukševičs E, Mark‐Kurik E. A near‐tetrapod from the Baltic Middle Devonian. Palaeontology. 2000 Sep; 43(3):533-48.
44. Poplin C, Lund R. Two new deep-bodied palaeoniscoid actinopterygians from Bear Gulch (Montana, USA, Lower Carboniferous). Journal of Vertebrate Paleontology. 2000 Sep 25;20(3):428-49.
45. Zhu M, Ahlberg PE. The origin of the internal nostril of tetrapods. Nature. 2004 Nov 4;432(7013):94-7.
46. Daeschler EB, Shubin NH, Jenkins Jr FA. A Devonian tetrapod-like fish and the evolution of the tetrapod body plan. Nature. 2006 Apr 6;440(7085):757-63.
47. Dietze K. A revision of paramblypterid and amblypterid actinopterygians from Upper Carboniferous–Lower Permian lacustrine deposits of Central Europe. Palaeontology. 2000 Nov;43(5):927-66.
48. Lu J, Giles S, Friedman M, den Blaauwen JL, Zhu M. The oldest actinopterygian highlights the cryptic early history of the hyperdiverse ray-finned fishes. Current Biology. 2016 Jun 20;26(12):1602-8.
49. Coates MI. Actinopterygians from the Namurian of Bearsden, Scotland, with comments on early actinopterygian neurocrania. Zoological Journal of the Linnean Society. 1998 Jan 1;122(1-2):27-59.
50. Coates MI. Endocranial preservation of a Carboniferous actinopterygian from Lancashire, UK, and the interrelationships of primitive actinopterygians. Philosophical Transactions of the Royal Society of London. Series B: Biological Sciences. 1999 Feb 28;354(1382):435-62.
51. Patterson C. Morphology and interrelationships of primitive actinopterygian fishes. American Zoologist. 1982 May 1;22(2):241-59.
52. Wiley EO. The phylogeny and biogeography of fossil and recent gars (Actinopterygii: Lepisosteidae). City University of New York; 1976.
53. Grande L, Bemis WE. A comprehensive phylogenetic study of amiid fishes (Amiidae) based on comparative skeletal anatomy. An empirical search for interconnected patterns of natural history. Journal of Vertebrate Paleontology. 1998 Apr 10;18(sup1):1-696.
54. Arratia G. Morphology, taxonomy, and phylogeny of Triassic pholidophorid fishes (Actinopterygii, Teleostei). Journal of Vertebrate Paleontology. 2013 Nov 1;33(sup1):1-138.
55. Maisey JG. Heads and tails: a chordate phylogeny. Cladistics. 1986 Sep;2(4):201-56.
56. Ahlberg PE, Clack JA. Lower jaws, lower tetrapods–a review based on the Devonian genus Acanthostega. Earth and Environmental Science Transactions of The Royal Society of Edinburgh. 1998 Jan;89(1):11-46.
57. Olsen PE, McCune AR. Morphology of the *Semionotus elegans* species group from the Early Jurassic part of the Newark Supergroup of Eastern North America with comments on the family Semionotidae (Neopterygii). Journal of Vertebrate Paleontology. 1991 Sep 30;11(3):269-92.
58. Lund R, Poplin C. The rhadinichthyids (paleoniscoid actinopterygians) from the bear gulch limestone of Montana (USA, lower carboniferous). Journal of Vertebrate Paleontology. 1997 Sep 4;17(3):466-86.
59. Coates MI, Sequeira SE. The braincase of a primitive shark. Earth and Environmental Science Transactions of the Royal Society of Edinburgh. 1998 Jan;89(2):63-85.
60. Coates MI, Sequeira SE. A new stethacanthid chondrichthyan from the Lower Carboniferous of Bearsden, Scotland. Journal of Vertebrate Paleontology. 2001 Aug 22;21(3):438-59.
61. Olsen PE. The skull and pectoral girdle of the parasemionotid fish *Watsonulus eugnathoides* from the Early Triassic Sakamena Group of Madagascar, with comments on the relationships of the holostean fishes. Journal of Vertebrate Paleontology. 1984 Nov 1;4(3):481-99.
62. Giles S, Friedman M. Virtual reconstruction of endocast anatomy in early ray-finned fishes (Osteichthyes, Actinopterygii). Journal of Paleontology. 2014 Jul;88(4):636-51.
63. De Pinna MC. Teleostean monophyly. Interrelationships of fishes. 1996 Jan 1:147-62.
