# Supplementary figures and images for: A new early actinopterygian from the Mid-Pennsylvanian Logan Quarry Shale member of Indiana
Source: PLoS One. 2025 May 7;20(5):e0320932. doi: 10.1371/journal.pone.0320932 (PMC12057928; doi:10.1371/journal.pone.0320932)

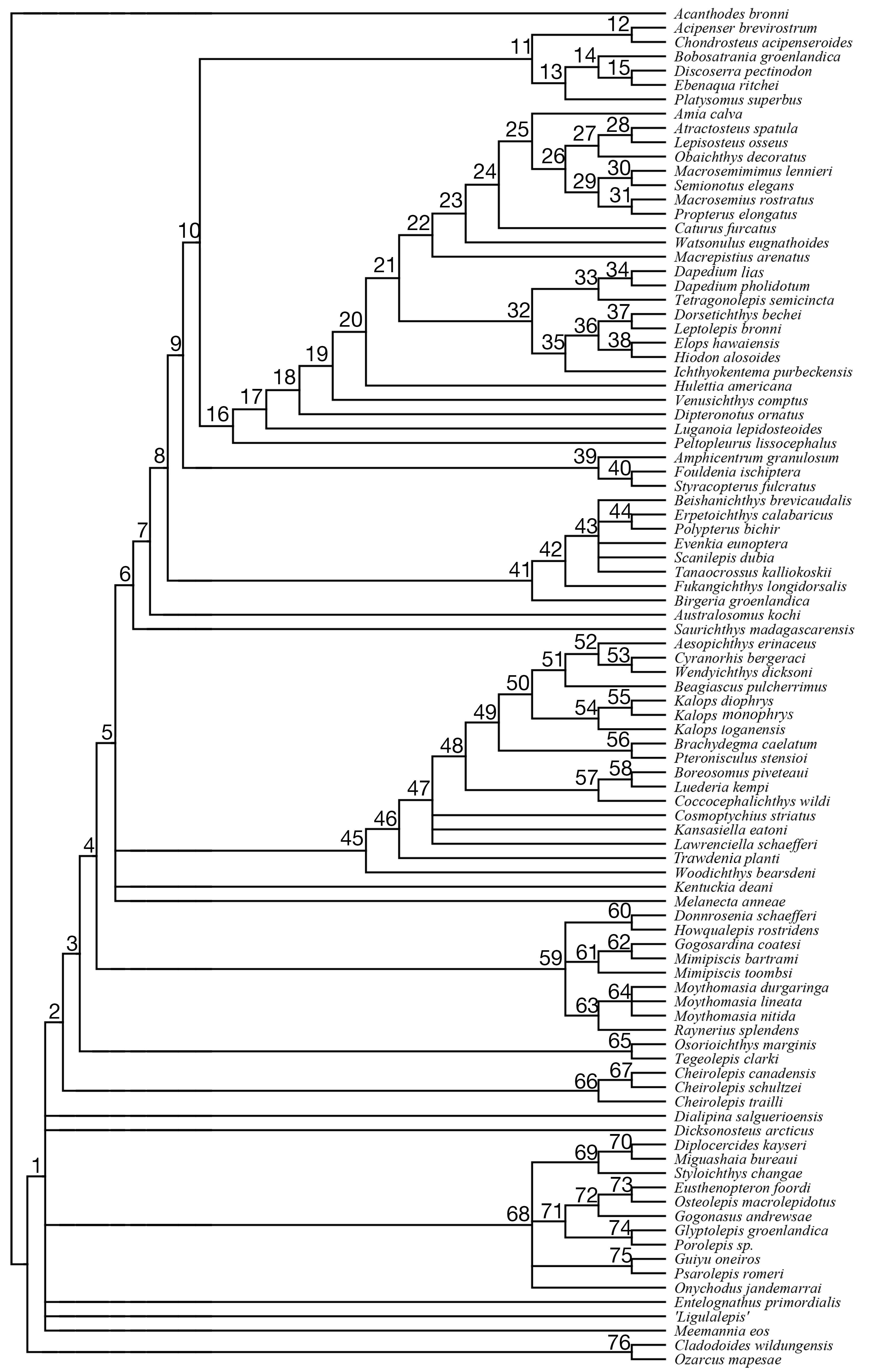

Supplement: S1 Fig — XXX. (TIF) [file pone.0320932.s003.tif]
