# Supplementary material for: A new early actinopterygian from the Mid-Pennsylvanian Logan Quarry Shale member of Indiana
Source: PLoS One. 2025 May 7;20(5):e0320932. doi: 10.1371/journal.pone.0320932 (PMC12057928; doi:10.1371/journal.pone.0320932)
Supplement: Attachment — Submitted filename: PONE-D-24-33140_reviewer.pdf [file pone.0320932.s005.pdf]

PLOS ONE

A new early Actinopterygii from the Mid-Pennsylvanian Logan Quarry Shale member of Indiana

--Manuscript Draft--

**Manuscript Number:**	PONE-D-24-33140
**Article Type:**	Research Article
**Full Title:**	A new early Actinopterygii from the Mid-Pennsylvanian Logan Quarry Shale member of Indiana
**Short Title:**	A new fossil fish from Indiana
**Corresponding Author:**	Chenchen Shen<br>University of Kansas Natural History Museum and Biodiversity Research Center: The University of Kansas Biodiversity Institute and Natural History Museum<br>Lawrence, Kansas UNITED STATES OF AMERICA
**Keywords:**	Carboniferous; Black Shales; Illinois Basin; North American Midcontinent Sea; stem-Actinopterygii
**Abstract:**	Kalops is a genus of early ray-finned fish in North America. It is characterized among early actinopterygians by the combination of the following characters: subterminal mouth, rounded oval shaped lacrimal, multiple small supraorbitals and suborbitals. So far, it has been only excavated from the Serpukhovian (Late Mississippian) Bear Gulch Limestone in Montana. Here, a new species of this genus, Kalops loganensis n. sp., is described based on two specimens from the Moscovian Logan Quarry Shales in Indiana. The phylogenetic analysis indicates that the new species is a member of Kalops and recovered as the sister of the other two species. This new species is the first record of Kalops outside of Bear Gulch. It provides evidence of survivability of this genus, and as the youngest member, proves that Kalops lived at least through the Mid-Pennsylvanian in North America.
**Order of Authors:**	Chenchen Shen
**Additional Information:**	
**Question**	**Response**
**Financial Disclosure**<br><br>Enter a financial disclosure statement that describes the sources of funding for the work included in this submission. Review the submission guidelines for detailed requirements. View published research articles from *PLOS ONE* for specific examples.<br><br>This statement is required for submission and **will appear in the published article** if the submission is accepted. Please make sure it is accurate.	Yes

Powered by Editorial Manager® and ProduXion Manager® from Aries Systems Corporation

**Funded studies**<br>Enter a statement with the following details:<br>• Initials of the authors who received each award<br>• Grant numbers awarded to each author<br>• The full name of each funder<br>• URL of each funder website<br>• Did the sponsors or funders play any role in the study design, data collection and analysis, decision to publish, or preparation of the manuscript?<br><br>Did you receive funding for this work?	
Please add funding details.<br>as follow-up to "**Financial Disclosure**<br><br>Enter a financial disclosure statement that describes the sources of funding for the work included in this submission. Review the submission guidelines for detailed requirements. View published research articles from *PLOS ONE* for specific examples.<br><br>This statement is required for submission and **will appear in the published article** if the submission is accepted. Please make sure it is accurate.<br><br>**Funded studies**<br>Enter a statement with the following details:<br>• Initials of the authors who received each award<br>• Grant numbers awarded to each author<br>• The full name of each funder<br>• URL of each funder website<br>• Did the sponsors or funders play any role in the study design, data collection and analysis, decision to publish, or preparation of the manuscript?<br><br>Did you receive funding for this work?"	2020, 2021, and 2022 Research Grants of the Association of Earth Science Clubs of Greater Kansas City;<br>2021 BI Panorama Grant of the department of Ecology & Evolutionary Biology, University of Kansas;<br>2022 and 2023 EEB Summer Funding, University of Kansas.
Please select the country of your main research funder (please select carefully as in some cases this is used in fee calculation).	UNITED STATES - US

as follow-up to "**Financial Disclosure**<br><br>Enter a financial disclosure statement that describes the sources of funding for the work included in this submission. Review the submission guidelines for detailed requirements. View published research articles from *PLOS ONE* for specific examples.<br><br>This statement is required for submission and **will appear in the published article** if the submission is accepted. Please make sure it is accurate.<br><br>**Funded studies**<br>Enter a statement with the following details:<br>• Initials of the authors who received each award<br>• Grant numbers awarded to each author<br>• The full name of each funder<br>• URL of each funder website<br>• Did the sponsors or funders play any role in the study design, data collection and analysis, decision to publish, or preparation of the manuscript?<br><br>Did you receive funding for this work?"	
**Competing Interests**<br><br>Use the instructions below to enter a competing interest statement for this submission. On behalf of all authors, disclose any competing interests that could be perceived to bias this work—acknowledging all financial support and any other relevant financial or non-financial competing interests.<br><br>This statement is required for submission and **will appear in the published article** if the submission is accepted. Please make sure it is accurate and that any funding sources listed in your Funding Information later in the submission form are also declared in your Financial Disclosure statement.	The authors have declared that no competing interests exist.

Powered by Editorial Manager® and ProduXion Manager® from Aries Systems Corporation

View published research articles from *PLOS ONE* for specific examples.<br><br>**NO authors have competing interests**<br><br>Enter: *The authors have declared that no competing interests exist.*<br><br>**Authors with competing interests**<br><br>Enter competing interest details beginning with this statement:<br><br>*I have read the journal's policy and the authors of this manuscript have the following competing interests: [insert competing interests here]*<br><br>* typeset	
**Ethics Statement**<br><br>Enter an ethics statement for this submission. This statement is required if the study involved:<br><br>• Human participants<br>• Human specimens or tissue<br>• Vertebrate animals or cephalopods<br>• Vertebrate embryos or tissues<br>• Field research<br><br>Write "N/A" if the submission does not require an ethics statement.<br><br>General guidance is provided below. Consult the submission guidelines for detailed instructions. **Make sure that all information entered here is included in the Methods section of the manuscript.**	N/A

Powered by Editorial Manager® and ProduXion Manager® from Aries Systems Corporation

**Format for specific study types**<br><br>**Human Subject Research (involving human participants and/or tissue)**<br>• Give the name of the institutional review board or ethics committee that approved the study<br>• Include the approval number and/or a statement indicating approval of this research<br>• Indicate the form of consent obtained (written/oral) or the reason that consent was not obtained (e.g. the data were analyzed anonymously)<br><br>**Animal Research (involving vertebrate animals, embryos or tissues)**<br>• Provide the name of the Institutional Animal Care and Use Committee (IACUC) or other relevant ethics board that reviewed the study protocol, and indicate whether they approved this research or granted a formal waiver of ethical approval<br>• Include an approval number if one was obtained<br>• If the study involved *non-human primates*, add *additional details* about animal welfare and steps taken to ameliorate suffering<br>• If anesthesia, euthanasia, or any kind of animal sacrifice is part of the study, include briefly which substances and/or methods were applied<br><br>**Field Research**<br><br>Include the following details if this study involves the collection of plant, animal, or other materials from a natural setting:<br>• Field permit number<br>• Name of the institution or relevant body that granted permission	
**Data Availability**<br><br>Authors are required to make all data underlying the findings described fully available, without restriction, and from the time of publication. PLOS allows rare exceptions to address legal and ethical concerns. See the PLOS Data Policy and FAQ for detailed information.	Yes - all data are fully available without restriction

A Data Availability Statement describing where the data can be found is required at submission. Your answers to this question constitute the Data Availability Statement and **will be published in the article**, if accepted.<br><br>**Important:** Stating 'data available on request from the author' is not sufficient. If your data are only available upon request, select 'No' for the first question and explain your exceptional situation in the text box.<br><br>Do the authors confirm that all data underlying the findings described in their manuscript are fully available without restriction?	
**Describe where the data may be found in full sentences. If you are copying our sample text, replace any instances of XXX with the appropriate details.**<br><br>• If the data are **held or will be held in a public repository**, include URLs, accession numbers or DOIs. If this information will only be available after acceptance, indicate this by ticking the box below. For example: *All XXX files are available from the XXX database (accession number(s) XXX, XXX.*).<br>• If the data are all contained **within the manuscript and/or Supporting Information files**, enter the following: *All relevant data are within the manuscript and its Supporting Information files.*<br>• If neither of these applies but you are able to provide **details of access elsewhere**, with or without limitations, please do so. For example:<br><br>*Data cannot be shared publicly because of [XXX]. Data are available from the XXX Institutional Data Access / Ethics Committee (contact via XXX) for researchers who meet the criteria for access to confidential data.*<br><br>*The data underlying the results presented in the study are available from (include the name of the third party*	All relevant data are within the manuscript and its Supporting Information files.

and contact information or URL).<br>• This text is appropriate if the data are owned by a third party and authors do not have permission to share the data.<br><br>* typeset	
Additional data availability information:	

Click here to access/download;Manuscript;Manuscript.docx

A new early Actinopterygii from the Mid-Pennsylvanian Logan Quarry Shale member of Indiana

Chenchen Shen[a,b]

[a] Department of Ecology and Evolutionary Biology, University of Kansas, Lawrence, Kansas, 66045, USA.

[b] Biodiversity Institute, University of Kansas, Dyche Hall, 1345 Jayhawk Blvd, Lawrence, Kansas, 66045, USA.

Email: c721s275@ku.edu

Abstract

Kalops is a genus of early ray-finned fish in North America. It is characterized among early actinopterygians by the combination of the following characters: subterminal mouth, rounded oval shaped lacrimal, multiple small supraorbitals and suborbitals. So far, it has been only excavated from the Serpukhovian (Late Mississippian) Bear Gulch Limestone in Montana. Here, a new species of this genus, *Kalops loganensis* n. sp., is described based on two specimens from the Moscovian Logan Quarry Shales in Indiana. The phylogenetic analysis indicates that the new species is a member of *Kalops* and recovered as the sister of the other two species. This new species is the first record of *Kalops* outside of Bear Gulch. It provides evidence of survivability of this genus, and as the youngest member, proves that *Kalops* lived at least through the Mid-Pennsylvanian in North America.

Key Words: Carboniferous; Black Shales; Illinois Basin; North American Midcontinent Sea; stem-Actinopterygii.

Introduction

The Carboniferous is the Late Paleozoic period when the marine ecosystem recovered from the mass extinction events at the end of the Devonian, which led to the extinction of about 90% of fish families (Friedman & Sallan, 2012). Recent studies suggest that the marine vertebrate ecosystem had fully recovered and established a high diversity of both bony and cartilaginous fishes by the end of the Mississippian (Early Carboniferous) (Sallan *et al*. 2011; Friedman & Sallan, 2012; Sallan & Friedman, 2012). Studies on

Serpukhovian fossil localities across North America and Europe suggest high richness in marine vertebrate (Wood, 1975; Coates, 1998; Lund & Grogan, 2015). Among them, one fossil Lagerstätte in North America, the Bear Gulch in Montana, has preserved an unmatchable high diversity of vertebrate fossils of the Serpukhovian (Late Mississippian). So far, there are 141 species of vertebrates, 55 Osteichthyes and 84 Chondrichthyes recovered from the Bear Gulch (Lund *et al*. 2012). The Chondrichthyes have wider geographical distribution (more than Bear Gulch) (Grogan *et al*. 2012), such as the Euro-North American *Harpagofututor volsellorhinus* (Lund, 1982), and *Thrinacoselache gracia* (Grogan & Lund, 2008). On the other hand, many actinopterygians excavated from the Bear Gulch, are restricted to this locality only, including the families Aesopichthyidae (Poplin & Lund, 2000), and Guildayichthyidae (Lund, 2000), as well as the genus *Kalops* (Poplin & Lund, 2002). So far, there are two species, *K. monophrys* and *K. diophrys* described. This genus is characterized by an elongated body form, subterminal mouth with the oral margin of the premaxilla facing posteroventrad, dorsal and ventral median rostral bones, short ventral dermosphenotic, multiple small supraorbitals, infraorbitals, and suborbitals. The limited information of *Kalops* hampers our understanding of the geographical and time distribution of this genus.

The mid-Pennsylvanian Black Shales in North America are widespread and combined with multiple regional geographical units across today's today's Oklahoma, Kansas, Missouri, Nebraska, Iowa, Illinois, and Indiana (Heckel, 1991). The 1-2-meter-thick shale is the evidence of the North American Mid-continent Sea (NAMS), which existed from Mid-Pennsylvanian to Early Permian, with a largest area nearly twice as

large as today's Hudson Bay (Algeo & Heckel, 2008). The Black Shales in the west Indiana, referred as the shallow sea of the Illinois basin (Fig. 1), are concentrated in three geological units. Two of them are from the Parke County, Indiana, the Mecca Quarry shale member of the Linton Formation overlayed on coal IIIA (Colchester Coal) and the Logan Quarry shale member of the Staunton Formation below coal III (Seelyville Coal) (Fig. 2). Both are dated as Moscovian in the Mid-Pennsylvanian (early Westphalian in the northwest European system) (Fig. 3) (Zangerl & Richardson, 1963; Zangerl & Case, 1973; Mastalerz & Harper, 1998; Mastalerz *et al.* 2018). The Logan Quarry shale member contains relatively complete and more delicately preserved actinopterygian specimens.

Figure 1. Paleogeographic reconstruction of North American Midcontinent Sea, approximately 309-310 Ma.

The black line highlighted the area of Illinois Basin. The little fish marks the Logan Quarry. The white diagonal line marks the paleoequator. Abbreviations: **IB**: Illinois Basin; **NAMS**: North American Midcontinent Sea. Figure modified with permission ©2023 Colorado Plateau Geosystems Inc. Scale bar = 500 km.

The Logan Quarry is one of the major Black Shales' localities in Parke County, Indiana. The marine vertebrate fossil-bearing section is also within the Logan Quarry shale member (Zangerl & Richardson, 1963). For the 46 to 55 cm thick Logan Quarry Shale member here, the vertebrate fossils are preserved in the 3.4 to 3.5 cm thick black sheety shale (Level J) of the Logan Quarry shale member (Zangerl & Richardson, 1963). Zangerl & Richardson (1963) proposed that the paleoenvironmental reconstruction of the Logan Quarry Shale member is a combination of near shore marine and fresh water

because the fossil content there includes terrestrial plants (especially leaves), invertebrates (annelids, mollusks and brachiopods) and vertebrates. This reconstruction was accepted by Coveney & Martin (1983) and Coveney & Glascock (1989). Zangerl & Richardson (1963) also indicated that the primary causation of the fragmented and disarticulated fossils in the Black Shales is predation. However, both hypotheses are ~~highly~~ questioned by more recent research. Studies of the fossil fauna and flora of the Upper Pennsylvanian Cohn Coal member in Illinois (Carpenter *et al*. 2011) and Joggins Formation in Nova Scotia in Canada (Falcon-Lang, 2005; Falcon-Lang *et al*. 2006), which have similar sedimentary facies with the Logan Quarry Shale member, showing that such fossil combination is an evidence of brackish water environment (Falcon-Lang, 2004). Elder & Smith (1984) were skeptical of the original hypothesis about the fossil fragmentation in the Black Shales and proposed that scavenging of the floating dead bodies is the dominant mechanism. The Logan Quarry Shale member is rich in vertebrate fossils including actinopterygians which are documented as “Palaeoniscoidea”; chondrichthyans (eg. *Petrodus*, *Listracanthus*), and pleuracanthid; acanthodians fragments; and sarcopterygians (possible rhipidistian) (Zangerl & Richardson, 1963; Zangerl & Case, 1973). Among them, cartilaginous fish fossils have already been recognized and studied such as *Deanea meccaensis* (Williams, 1985), *Rhombacanthus zangerli* (Williams, 1985), *Ornithoprion hertwigi* (Zangerl, 1966), and *Cobelodus aculeatus* (Zangerl & Case, 1976). However, there is no equivalent study of the ray-finned fish (Actinopterygii) from the Black Shales. Until now, Poplin (1978) is the only study of the actinopterygians from the Logan Quarry, which briefly described a long

rostrum specimen based on X-ray radiograph. Study the fossil actinopterygians from the Logan Quarry is critical to understanding the biodiversity of the Black Shales.

Figure 2. The Pennsylvanian (Late Carboniferous) chronostratigraphic chart, modified from Lucas *et al*. (2022).

The arrow marks the age of the Logan Quarry.

Here, I report a new species of *Kalops* based on two laterally preserved specimens excavated from the mid-Pennsylvanian (Late Carboniferous) Black Shales unit, Staunton Formation in Logan Quarry, Indiana. The new materials extend the temporal of *Kalops* to Mid-Pennsylvanian, and slightly expand their geographical distribution to the eastern boundary of the NAMS.

Figure 3. Approximated stratigraphic correlation of Desmoinesian stage among Indiana, Kansas, Illinois, and New Mexico states.

The information of regional stratigraphy units and their correlations are based on multiple previous works including Kosanke et al. (1960), Mastalerz & Harper (1998), Mastalerz et al. (2018), Schneider et al. (2020), and Lucas et al. (2021). The horizon of the Logan Quarry Shale member is highlighted in yellow. Abbreviations: **Fm**: formation; **Gr**: group; **m**: member.

Material and Method

Material. The fossil *Kalops* specimens from the Logan Quarry are housed in the Field Museum of Natural History (FMNH), Chicago, Illinois with the following catalogue numbers: FMNH PF- 2286, and FMNH PF-5919.

Methods. All specimens were already well prepared before this study started. No further preparation was conducted. The dusting of the fossils with NH_4Cl was made to facilitate the observation and photographing of specimens that are naturally black. Line drawings of each specimen were executed under a ZEISS 47 50 52 microscope with a camera lucida attachment, then final figures were prepared with Adobe Photoshop. Photographs were taken with a Sony A-73 camera under natural light or electric light sources.

Phylogenetic method. The phylogenetic analysis was conducted by adding the new species into the matrix of Giles *et al*. (2017), with all the other settings remaining the same. Giles *et al*. (2017) performed a comprehensive phylogenetic analysis of early Osteichthyes by using 265 morphological characters, and both *K. monophrys* and *K. diophrys* were included in the analysis. The parsimony analysis was conducted using PAUP (4.0a, build 169) in windows-based version. All the characters are unordered and have equal weight.

Terminology. The terminology used for skull bones based on homology follows Schultze (2008). The traditional actinopterygian skull terminology is shown in parentheses after the corrected ones, such as postparietal (= parietal). The terminology for complex bones in the snout region follows Mickle (2012, 2015), and for the dermosphenotic follows Poplin (2004). The terminology for scales follows Schultze

(1966, 1996, 2015) and the squamation regions follow Esin (1990) (Fig. 4). The terminology for fin rays, scutes and fulcra follows Arratia (2008, 2009).

Figure 4. Actinopterygian scale regions as suggested by Esin (1990). A-G are the lateral flank scales; I-IV are the ridge scutes; “i” is preanal scute.

Systematic Paleontology

OSTEICHTHYES Huxley, 1880

ACTINOPTERYGII Cope, 1887

Kalops Poplin & Lund, 2002

Type Species. *Kalops monophrys* Poplin & Lund, 2002.

New Species: *Kalops loganensis* n. sp.

Etymology. After Logan Quarry, Parke County, Indiana, USA. where the specimens were excavated.

Diagnosis (based on a unique combination of characters). A ~~typical medium sized~~ kalopid fish of about 160 mm long length; with a fusiform body and a sharp snout; the rostral region includes a dorsal median rostral, a ventral median rostral, and paired premaxillae; the parietal bone is elongated; the postparietal is shorter than the parietal; four extrascapulae, two on each side of the skull; the lacrimal is a distinct and separate bone, elongated and oval shaped; three small infraorbitals between jugal and dermosphenotic; the rectangular shaped dermosphenotic is short and deep; no dorsal

dermosphenotic (= intertemporal); five small suborbitals in a vertical row between preopercle and infraorbitals; eight small supraorbitals in a horizontal row, forming the dorsal margin of the orbit; the oral margin of the upper jaw is formed by ventral rostro-premaxilla, lacrimal and maxilla, all bearing teeth; maxilla shorter than the lower jaw; maxilla has a deep posterior plate and a deep posteroventral process; the anterior margin of the maxilla is ventral to the orbit; nine branchiostegal rays; body fully covered with rhombic scales with ganoine; the dorsal and ventral ridges of the body are covered by scutes; the posterior edge of the scales are straight; the predorsal ridge scutes have the same size as the scale rows ventral to them; all scales and scutes are heavily ornamented by continuous ridges of ganoine, the anterior ends of these ridges are finely crenulated.

Several features distinguish the new species from the Bear Gulch *Kalops,* including the absence of median extrascapular and of presupracleithrum. Additionally, all infraorbitals are of similar size.

Holotype. FMNH PF-2286, an almost complete specimen preserved as part and counterpart. The pelvic and anal fins are not preserved, and the caudal fin is only partially preserved in the hypaxial lobe.

Type locality. Mid-Pennsylvanian Staunton Formation, Logan Quarry Shale member, collected from Level J in the Logan Quarry, Parke County, Indiana.

Anatomical description

General morphology and size. This taxon is a medium-sized fish. Although both the FMNH PF-2286 and the FMNH PF-5919 specimens are incomplete, FMNH PF-2286 is

160 mm long from the snout to the posterior, before the epaxial base of caudal fin, whereas FMNH PF-5919 is 150 mm long, including the posterior half of the skull and the body trunk until the squamation hinge line. FMNH PF-2286 is longer, the specimen FMNH PF-5919 lost more sections at its anterior and posterior ends. Thus, I assume that the standard length of FMNH PF-5919 is slightly longer than FMNH PF-2286. The body shape is fusiform. In FMNH PF-2286 the head length is 45 mm, relatively short compared to the total length of this specimen. The fish has a sharp snout, short preorbital length and orbital diameter, about 11 % and 22 % to the head length. The skull dermal bones are covered with ganoine. The caudal fin is heterocercal. The body is fully covered by ganoid-type scales (Fig. 5). The scale formula is:

$$\frac{D20\ or\ 23}{P10\ A?\ C37\ or\ 42}T?.$$

Figure 5. ***Kalops loganensis*** **n. sp.**

A. Photograph of the holotype FMNH PF-2286, part; **B.** Photograph of the holotype FMNH PF-2286, counterpart; **C.** Photograph of specimen FMNH PF-5919. Scale bar = 10 mm.

Snout. The snout of *Kalops loganensis* is the same as *K. monophrys* and *K. diophrys*. The snout is sharp, and it is formed by a dorsal median rostral, a ventral median rostral, paired premaxillae, and nasal bones (Fig. 6).

The dorsal median rostral is incomplete and only preserves its ventral part. This bone sutures with the nasal bones laterally and separates the nasal bones from contacting each other, and the ventral median rostral ventrally. The notch for the anterior nostril is on the lateroventral border of the dorsal median rostral. The ventral median rostral is

shallow and wide, about twice the width of the dorsal median rostral. It is almost rectangular shaped. Its dorsal margin contacts both the dorsal rostral and the nasal bone, the posterior margin contacts the lacrimal (= infraorbital one), and the ventral margin contacts the premaxillae. The ventral margin is slightly convex. The ethmoid commissure is placed horizontally in the ventral rostral and across the midline of this bone. The nasal bone is deep, narrow and crescent shaped. It is deeper than the dorsal rostral. It contacts the parietal anterodorsally, and the first supraorbital posteriorly. The nasal bone does not connect with the dermosphenotic. The posterior nostril is located at the posteroventral border of the nasal bone. ~~It is slightly more dorsal than the anterior nostril.~~ All these bones are heavily ornamented by continuous and connected ridges of ganoine. In the nasal bone the supraorbital sensory canal is placed almost in a straight line.

The premaxilla is small, shallow and elongated that contacts the lacrimal posteriorly. There are no teeth preserved on the ventral margin of the premaxilla.

Figure 6. Head of *Kalops loganensis* n. sp.

A. Photograph of specimen FMNH PF-2286, part; **B.** Line drawing based on specimen FMNH PF-2286, part. Abbreviations: **ang**: angular; **brs**: branchiostegal rays; **d.ro**: dorsal median rostral; **dhy**: dermohyal; **dsp**: dermosphenotic; **dspl**: dentalosplenial; **esc**: extrascapulae; **io**: infraorbitals between jugal and dermosphenotic; **io2**: infraorbital 2; **ju**: jugal; **la**: lacrimal; **mx**: maxilla; **na**: nasal; **op**: opercle; **pa**: parietal; **par**: parasphenoid; **pec.f**: pectoral fin; **pmx**: premaxilla; **pop**: preopercle; **ppa**: postparietal; **pt**: posttemporal; **sc**: scales; **scl**: supracleithrum; **sob**: suborbital; **sop**: subopercle; **stt**: supratemporotabular; **sub**: supraorbital; **v.ro**: ventral median rostral. Scale bar = 5 mm.

Skull roof. The skull roof is elongated and drastically expands laterad at the position of the supratemporotabulars (= dermopterotics). The region includes the parietal (= frontal), the postparietal (= parietal), the supratemporotabular, and the extrascapular bones (Fig. 6).

The parietal is lost in FMNH PF-5919 and is only partially preserved in FMNH PF-2286. It is an elongated bone, about 34 % of the skull length and its own length/width ratio is 32 %. This bone is sutured with the dorsal median rostral anteriorly, the nasal, the dermosphenotic, the supratemporotabular, and potentially the supraorbitals one to five laterally, and the postparietal posteriorly. Due to the preservation, all the sutures, as well as the position of the pineal opening are unclear. The postparietal is a short rectangular bone. It is about 28 % as long as the parietal but slightly wider than it. The postparietal sutures with the supratemporotabular laterally, and the extrascapulae posteriorly throughout dentated and/or serrated margins (*sutura dentata* type). The dorsal dermosphenotic (= intertemporal) is absent. The supratemporotabular has an irregular shape, with its anterior portion wider than the posterior part. It has a very short suture with the lateral extrascapular posteriorly. The ventral edge of the supratemporotabular has a shallow and blunt process facing laterad but has no contact with the dorsal edge of the dermohyal. There are two pairs of extrascapulae on each side of the skull. They form one vertical row. All extrascapulae are square shaped, while the lateral ones are slightly larger. Both articulate with the posttemporal posteriorly and form a zigzagged margin. The medial extrascapulae also suture to each other medially throughout a *sutura dentata*. All the skull roof bones are heavily ornamented with short ridges of ganoine except the postparietal, whose ornamentation is not preserved.

The trajectory of the supraorbital canal in the parietal is bent in the orbital region. In the postorbital region, this canal is horizontal and straight in both parietal and postparietal. The supraorbital canal ends in the postparietal around its mid region. Posterior to the supraorbital canal, there is a very short anterior pit line, followed by a longer median pit line, which is still restricted to the postparietal. The posterior pit line cannot be seen in any of the specimens.

The otic canal is placed almost horizontally in the supratemporotabular, near its medial border. The posterior part of the otic canal is positioned in the lateral extrascapular. This section is short and straight, about half the length of the lateral extrascapular. The posterior section of the cephalic sensory system is tripartite with the lateral line canal and the supratemporal commissure, which is vertically straight and crosses both the lateral and medial extrascapulae.

Neurocranium. Unfortunately, there is no observable neurocranium preserved for this taxon. An elongated and narrow element horizontally crosses the orbit in FMNH PF-2286 and is identified as the parasphenoid (Fig. 6).

Figure 7. *Kalops loganensis* n. sp.

Skull photograph of specimen FMNH PF-2286, counterpart. Scale bar = 5 mm.

Circumorbital bones. The circumorbital ring is formed by the following bones: the nasal anteriorly, the lacrimal, the infraorbital two, and the jugal (= infraorbital three) ventrally, the three small infraorbitals and the dermosphenotic posteriorly, and the supraorbitals posterodorsally and dorsally. The sclerotic bones are not preserved in any of the specimens (Fig. 6).

The lacrimal is an oval shaped bone and it is elongated on the antero-posterior axis. The posterior margin of this bone is in contact with both the infraorbital two and the maxilla. The ventral margin of the lacrimal is involved in the oral rim of the upper jaw. Two or three small conical teeth with acrodin cap are placed on the anterior portion of the ventral margin of the lacrimal. All teeth face ventrad. The infraorbital two is narrow and bar shaped. It is horizontally placed between the lacrimal and the jugal, and dorsal to the suborbital arm of the maxilla. The jugal has a nearly trapezoid shape, with its dorsal part shorter than the ventral part. There are three small irregular shaped infraorbitals placed vertically between the dorsal margin of the jugal and the ventral margin of the ventral dermosphenotic. Their size is gradually decreasing from the ventral one to the dorsal one. The dermosphenotic is short and deep, slightly obliquely placed at the posterodorsal corner of the orbit. It has a convex posterior border articulating with the supratemporotabular and a concave anterior border. The dorsal margin of the orbit is formed by eight small rounded supraorbitals, which are arranged into one horizontal row. The first five supraorbitals are placed lateroventrally to the parietal, whereas the last three are connected to the anteroventral margin of the dermosphenotic. Posterior to the jugal and the three small infraorbitals, there is one vertical row with five small suborbitals. The dorsal most suborbital one is the smallest. It is not in contact with the dermosphenotic on its dorsal edge. The ventral most suborbital five is the biggest among them.

The infraorbital canal can be clearly seen on FMNH PF-2286. In general, this canal is narrow and simple, without ramifications. The canal meets with the vertical supraorbital canal, and the posterior extension of the ethmoid commissure, to form the

tripartite canals in the anterior part of the lacrimal. At the midventral orbital region, the infraorbital canal is placed horizontally through the lacrimal and the infraorbital two, as well as the anterior most part of the jugal. In the jugal the infraorbital canal bends vertically. The infraorbital canal is still vertical across the three infraorbitals between jugal and dermosphenotic. In the ventral dermosphenotic, the infraorbital canal connects with the otic canal near the posterodorsal margin of this bone.

Figure 8. *Kalops loganensis* n. sp. Skull bones, line drawing based on specimen FMNH PF-2286, counterpart.

This drawing only includes the large identifiable elements. Note the strong ornamentation on these bones. Abbreviations: **dspl**: dentalosplenial; **mx**: maxilla; **op**: opercle; **sop**: subopercle. Scale bar = 5 mm.

Upper jaw. The upper jaw is formed by the premaxilla, the lacrimal, and the maxilla. In general, the oral rim of the upper jaw is longer than the lower jaw, bearing an anteroventral notch at the lateral profile of the skull (Fig. 6).

The maxilla is the largest bone in the upper jaw. It has two distinct sections: the suborbital arm and the posterior plate. The maximum length/depth ratio of this bone is almost 2.3. The narrow, long and straight suborbital arm contributes to the anterior half of the maxilla, its depth is gradually increasing from anterior to posterior. The anterior tip of the suborbital arm is ventral to the orbital ring. Its depth gradually increases from anterior to posterior, and the highest point of its dorsal margin is lower than the midline of the orbital ring. The posteroventral process on the posterior plate is short and deep, about half as depth as the posterior plate. On the oral margin of the maxilla, from its anterior most

part until the beginning of the posteroventral process, there is one row of sharp, straight, and narrow conical teeth with acrodin cap. The posterior plate of the maxilla is strongly ornamented by continuous short ridges of ganoine. However, the ornamentation of the suborbital arm is unclear (Figs. 6, 7, 8, 9, 10, 11).

Figure 9. *Kalops loganensis* n. sp. Skull photograph of specimen FMNH PF-5919. Scale bar = 5 mm.

Lower jaw and suspensorium. The lower jaw consists of a dentalosplenial (= dentary) and the angular. The lateral preservation of the specimens studied prevents any further observation of the elements in medial side (Figs. 6, 9, 10).

The dentalosplenial is elongated, over half of the skull length. The depth of the dentalosplenial gradually increases from anterior to posterior. The oral margin and the teeth condition are unknow because these parts are covered by the upper jaw in all specimens. Sutured posteriorly to the dentalosplenial, there is the angular, which is about ¼ as long as the dentalosplenial. Their suture is wavy and “s” shaped. These two bones are strongly ornamented with obliquely oriented long ridges of ganoine. In all specimens the oral margin of the lower jaw is covered by the upper jaw, preventing any observation of the dentition.

The mandibular canal can be only seen in its posterior part, restricted to the angular. This shortly exposed section is straight on its anterior part and anteroventro-posterodorsally oblique at its posterior part.

Figure 10. ***Kalops loganensis*** **n. sp. Skull line drawing based on specimen FMNH PF-5919.**

Abbreviations: **1st br**: first branchiostegal ray; **2nd br**: second branchiostegal ray; **ang**: angular; **brs**: branchiostegal rays; **cl**: cleithrum; **clv**: clavicle; **dhy**: dermohyal; **dspl**: dentalosplenial; **mx**: maxilla; **op**: opercle; **pop**: preopercle; **pt**: posttemporal; **qj**: quadratojugal; **scl**: supracleithrum; **sop**: subopercle. Scale bar = 5 mm.

Opercular bones, branchiostegal rays, and gular plates. The bones described in this section include the preopercle, the opercle, the subopercle, the dermohyal, and the branchiostegal rays. The gular plates are not preserved (Figs. 6, 7, 9, 10).

The preopercle has a long and broad anterodorsal branch and a narrow and short ventral branch. The angle between these two branches is 90 degrees. The preopercle does not contact neither the dermosphenotic, nor any of the skull roof bones. The anterodorsal branch is large, over half as deep as the orbital depth and as long as the length of the posterior plate of the maxilla. The anterior margin of the anterodorsal branch is vertical and straight. The ventral branch is about half as deep as the posterior plate of the maxilla. Its ventral margin is nearly at the same horizon of the oral rim of the upper jaw. A relatively large and splint like dermohyal is located between the posterodorsal margin of the preopercle and the anterior margin of the opercle. The opercle is trapezoid and deep, having a depth/length ratio of 1.5. The ventral margin is slightly longer than the dorsal margin. This bone articulates with the posttemporal posterodorsally, the supracleithrum posteriorly, and the subopercle ventrally. The dorsal margin of the opercle is higher than the dorsal margin of the dermohyal. The opercle has concave anterior and ventral margins, and a slightly convex posterior margin. The

subopercle is wider and shallower than the opercle. In general, the size of the subopercle is over half than that of the opercle. The posterior margin of the subopercle is longer than the anterior margin, being its dorsal margin obliquely concave. The subopercle in the holotype has an anteroventral process but lacks an anterodorsal process.

The branchiostegal rays are preserved better in FMNH PF-5919 with nine branchiostegal rays extending from the ventral part of the subopercle to the posteroventral part of the lower jaw. The first branchiostegal ray is the largest ray among the nine, and it is oval shaped, with the anterior part larger than the posterior part. Other branchiostegal rays are elongated and rectangular shaped. The branchiostegal rays are ornamented with dense short ridges of ganoine, as well as the opercle and the subopercle. The preopercle only has ornamentation on the anterodorsal branch.

The preopercular canal is only preserved as a short section in the ventral branch of the preopercle in FMNH PF-5919.

Figure 11. *Kalops loganensis* n. sp. A separate maxilla on specimen FMNH PF-2286, in medial view.

A. Photograph, PF-2286 part; **B.** Line drawing based on specimen FMNH PF-2286, counterpart. Abbreviations: **hll**: horizontal longitudinal lamina; **po.pl**: posterior plate; **pv.pr**: posteroventral process; **sob.ar**: suborbital arm. Scale bar = 5 mm.

Pectoral girdle and fin. The following bones form the pectoral girdle, posttemporal, supracleithrum, cleithrum, and clavicle.

The posttemporal is a nearly triangular bone with an antero-posterior elongation. This bone articulates with the lateral and medial extrascapulae anteriorly, and the supracleithrum posteroventrally (Figs. 6, 9, 10). *K. loganensis* n. sp. does not have presupracleithrum.

The supracleithrum is rectangular shaped, narrow, deep, and obliquely placed. It is shallower than the opercle and articulates with the opercle anteriorly. Ventrally the supracleithrum covers the dorsal most part of the cleithrum (Figs. 6, 9, 10). The cleithrum is incomplete in FMNH PF-5919, but there is a disarticulated cleithrum in FMNH PF-2286, which is completely preserved (Fig. 12). This bone is deep and narrow, with a dorsal and a ventral section. The dorsal section occupies over 2/3 of the depth of the bone. It is almost triangular shaped, having a pointed dorsal tip, which is also bending anteriad. The ventral portion of the dorsal section bends anteriad as well and connects to the ventral section of this bone. The ventral section of the cleithrum is rectangular, about the same length as the dorsal section. The cleithrum has a convex anteroventral margin and a 90-degree wide posteroventral notch, where the pectoral fin is attached here (Fig. 12). The clavicle has only its posterior most part preserved in FMNH PF-5919. That part is articulated with the cleithrum anteroventrally. Among the dermal bones of the girdle, only the supracleithrum and the cleithrum are heavily ornamented with short and long ridges of ganoine.

The pectoral fin is preserved *in situ* in FMNH PF-5919, but the condition is too poor to get any details of its structure. Fortunately, in the FMNH PF-2286 there is a relatively more complete external mold of the pectoral fin. This fin is placed near the ventral margin of the fish. There is no lobe structure attached to its base. In general, the

fin is short, only 12 % of the specimen's length, which means the value of the pectoral fin length/standard length ratio is even lower. It has 13 or 14 fin rays. All are segmented from proximal to distal. The marginal rays are longer than the last few rays, giving a triangular lateral profile to the pectoral fin. The condition of the basal fulcra and fringing fulcra is unknown.

Figure 12. *Kalops loganensis* n. sp. An isolated cleithrum on specimen FMNH PF-2286.

A. Photograph, PF-2286, part; **B.** Line drawing based on PF-2286, part. Scale bar = 5 mm.

Pelvic girdle and fin. There is no pelvic girdle available in the available specimens because that area is fully covered by the scales. The pelvic fin is poorly preserved in FMNH PF-5919. It originates at the 10th vertical scale row. Only three fin rays are preserved, which are segmented proximo-distally.

Median fins. The anal fin is lost on all specimens. The dorsal fin in FMNH PF-5919 has a relatively better preservation, although it is still incomplete. The dorsal fin originates at the 20th vertical scale row in the FMNH PF-2286 and at the 23rd vertical scale row in FMNH PF-5919. Its lateral profile is probably triangular shaped. The fin has one small basal fulcrum and followed by 13 or 14 rays segmented from proximal to distal. Fringing fulcra are unknown on the dorsal fin.

The caudal fin is lost on FMNH PF-5919, but the hypaxial lobe is preserved partly on FMNH PF-2286. The hypaxial lobe has two basal fulcra, and 29 rays. Among them, seven rays are segmented but not branched thus they are confirmed as procurrent

rays. The most distal lepidotrichial segments are spine-like and longer than the proximal segments. Small fringing fulcra appear on the ventral margin of the marginal ray segments of the procurrent rays, between the most distal segments. The fringing fulcra are rhomboid shaped and swollen distally. Each marginal ray segment aligns with one fringing fulcrum. The epaxial lobe is only preserved as five long basal fulcra.

Squamation. The body is fully covered with rhomboid scales with ganoine (Figs. 13 and 14). There are approximately 40 vertical scale rows between the skull and the hinge line. The mid-flank scales of the first seven vertical rows close to the skull are trapezoid and deepened (Figs. 13A and 14A). Their ventral margin is longer than the dorsal margin. These scales also have a maximum depth/length ratio of 1.5. Starting from the first vertical row, this ratio of the deepened scales decreases, until the eighth row, where the ratio reaches 0.7. The deepened scales also have the peg-and-socked articulation and the anterodorsal process. The ornamentation consists of long and short ridges of ganoine. Most of them are anterodorso-posteroventrally oriented. The mid-flank scales from the eighth vertical row to the 14th row are nearly rectangular shaped but have their anterior part bends dorsad (Figs. 13B and 14B). Their posterior margin is deeper than the anterior margin. These scales also have the peg-and-socket articulation. The ornamentation is formed by long ridges (over half of the scale's length) of ganoine. These ridges are tightly arranged with an anterodorso-posterovental orientation. Starting with the 15th vertical row, the mid-flank scales are elongated rhomboid shaped and without peg-and-socket articulation (Figs. 13C and 14C). They have the same ornamentation pattern as the scales from the eighth to the 14th row. But there are small brushes like crenulations at the anterior end of the ridges. The scales near the dorsal margin of the body and anterior to

the dorsal fin are triangular shaped without peg-and-socket articulation (Figs. 13E and 14E). Their ornamentation consists of long ridges tightly arranged and oriented anterodorso-posteroventrally. The four horizontal rows of the scales near the dorsal margin of the body trunk, posterior to the origin of the dorsal fin (Figs. 13G and 14G), and the five horizontal rows of the scales near the ventral margin of the body (Fig. 13F), are highly elongated, with a depth/length ratio as low as 0.17. They do not have the peg-and-socket articulation but have an anterodorsal process. The ornamentation of these scales is the same as the scales on the same vertical row.

The dorsal and ventral scutes have various morphologies. The dorsal scutes anterior and posterior to the dorsal fin are rhomboid and have an antero-posterior elongation (Figs. 13 I, II and 14 I, II). Their size is nearly the same as the scales ventral to them. The ornamentation consists of long ridges of ganoine tightly arranged, and oriented antero-posteriorly. The ventral scutes at the anterior part of the body, probably anterior to the position of the anal fin, are gigantic, over five times as large as the dorsal scutes. The only complete one shows an oval shape with the antero-posterior elongation, having its anterior end broader than the posterior one (Figs. 13 III and 15A). The ornamentation consists of highly dense long ridges of ganoine tightly arranged. There is one mid ridge from the posterior end of the scute until the mid-region. Almost all the other ridges are the branches of the mid ridge and parallel to the rounded lateral margins of the scutes. On the anterior tips of these ridges there are several short parallel grooves, giving fine crenulations on the anterior end of these ridges. The ventral scutes between the anal fin and the caudal fin are slightly smaller than the ventral scutes described above. Their shape is like an elongated water drop, with a broader anterior part and narrower posterior

end (Figs. 13 IV and 15B). The posterior tip is bifurcated. The ornamentation is a combination of short grooves on the posterior half, and the dense long ridges of ganoine on the anterior half. The anterior tips of these ridges are also finely crenulated.

The trajectory of the lateral line on the body trunk is unclear. There is no scale showing lateral line openings or pit organ pores.

Figure 13. *Kalops loganensis* n. sp. Line drawings of the scales from specimen FMNH PF-5919.

The letters are marking the regions in Figure 5. Scale bar = 1 mm.

Figure 14. *Kalops loganensis* n. sp. Photographs of the scales in specimen FMNH PF-5919.

The letters are marking the regions in Figure 5. Note the scales sizes are not in scale.

Figure 15. *Kalops loganensis* n. sp. Photographs of the scutes on specimen FMNH PF-5919.

A. Same scute in Figure 13, III; **B.** Same scute in Figure 13, IV. Scale bar = 1 mm.

Results of phylogenetic analysis

The parsimony phylogenetic analysis is recovered by 1351 most parsimonious trees. The topology of the strict consensus tree is shown in Figure 16. The tree length is 1351, the consistency index (CI) is 0.2184, and the retention index (RI) is 0.6450. The result of my phylogenetic analysis indicates that *Kalop loganensis* n. sp. is a member of *Kalops* (Fig. 16, node B), and stands as the sister taxon of the two Bear Gulch *Kalops* (Fig. 16, node C). The *Kalops* node is supported by nine synapomorphies, which all are homoplastic:

premaxilla is confined to the region anterior to the orbit (Ch. 6 [1]); parietal (= frontal) is shorter than postparietal (parietal) (Ch. 28 [0]); parietals (= frontals) broad posteriorly and tapering anteriorly (Ch. 29 [0]); the infraorbital canal in jugal does not have ramification (Ch. 55 [0]); three or more supraorbitals (Ch. 58 [2]); the opercle is at least twice as high as the subopercle (Ch. 110 [0]); subopercle has anteroventral process (Ch. 113 [1]); opercular process is absent (Ch. 211 [0]); pectoral fin endoskeleton extends far beyond body wall (fins lobate) (Ch. 238 [0]). The clade of *K. monophrys* and *K. diophrys* is supported by two homoplastic features: one pair of extrascapulae (Ch. 43 [0]); a single median extrascapular is present (Ch. 45 [0]). The only autapomorphy of *K. loganensis* n. sp. is the absence of presupracleithrum (Ch. 226 [0]). The *Kalops* clade is nested within the monophyletic group that most of the members, except *K. loganensis* n. sp., are from the Serpukhovian Bear Gulch Limestone (Fig 16. node A). This node is supported by two homoplastic characters: the mandibular canal traces the ventral margin of the lower jaw along the entire length (Ch. 76 [0]); one infradentary (angular only) (Ch. 89 [2]). For the synapomorphies of other nodes, see Giles *et al*. (2017).

Figure 16. Phylogenetic relationships of *Kalops loganensis* n. sp. and its relatives. The data matrix is based on 95 taxa and 265 characters, and *Acanthodes bronni* is selected as the outgroup. Strict consensus tree of 1351 most parsimonious trees. Tree length is 1351, consistency index (CI) = 0.2184, retention index (RI) = 0.6450. Numbers under the nodes are the bootstrap values higher than 50%. The *Kalops* clade is highlighted in green, and its relatives are highlighted in yellow.

Discussion

Comparison with the other two *Kalops* species. *Kalops loganensis* n. sp. appears to be the largest kalopid fish, longer than the 116mm *K. monophrys* and 96mm *K. diophrys*. Poplin & Lund (2002) listed several features that distinguish *Kalops* from other early actinopterygians, including short dermosphenotic, nasal does not contact to dermosphenotic, five to six infraorbitals, subterminal mouth, and lobed pectoral fin. Poplin & Lund (2002) also emphasizing three features that are the most obvious ones, including small numerous supraorbitals, small numerous suborbitals, and accessory row of extrascapulae.

K. loganensis n. sp. shares almost all features with other *Kalops* except the pectoral fin lobe, which remains unknown due to the poor preservation, and the accessory extrascapulae row, which is only present in *K. diophrys* and absent in both *K. monophrys* and *K. loganensis* n. sp.

The dermosphenotic in *K. loganensis* n. sp. is short compared to the surrounding skull roof bones, more similar to *K. monophrys*, while *K. diophrys* has a longer dermosphenotic.

All the three *Kalops* have lacrimal, infraorbital 2, and jugal. But the conditions between jugal and dermosphenotic are different. *K. monophrys* and *K. loganensis* n. sp. have three infraorbitals in this region, while *K. diophrys* has four. *K. loganensis* n. sp. has the fewest number of supraorbitals, eight forming one row, *K. monophrys* has 13 supraorbitals in one row, and *K. diophrys* has 22 in two rows, 12 in the upper row, and ten in the lower row.

The suborbitals are different in all species. *K. loganensis* n. sp. has five suborbitals, placed in a vertical row, with reduced size from ventral to dorsal. The ventral

most suborbital is two times larger than the dorsal most one. *K. monophrys* has five suborbitals but they are oriented in an obliquely vertical row. The ventral most suborbital is more than ten times larger than the dorsal most suborbital. *K. diophrys* has the most numerous suborbitals, 12 of them organized in three vertical rows. The largest suborbital is still the most ventral one, nearly ten times larger than the smallest suborbital bone.

Another feature that is different across all species is the extrascapulae. A median extrascapular appears in both Bear Gulch *Kalops* but lacks in *K. loganensis* n. sp. *K. loganensis* n. sp. has two pairs of lateral extrascapulae and no accessory extrascapulae. *K. monophrys* and *K. diophrys* have one pair of lateral extrascapulae. In addition, *K. diophrys* has one vertical row of five small accessory extrascapulae, anterior to the lateral extrascapular. One specimen of *K. monophrys*, Carnegie Museum CM35395, preserved one small, rounded bone surrounded by lateral extrascapular, postparietal, and supratemporotabular, and it was identified by Poplin & Lund (2002) as an accessory extrascapular. However, this feature was only present on one specimen (Poplin & Lund, 2002, fig.2-2) without any description. Thus, this specimen needs further examination.

Phylogenetic assessment. Across different earlier analyses, the phylogenetic position of *Kalops* drifts within the stem Actinopterygii group. It was recovered as the sister group of other Bear Gulch taxa such as *Aesopichthys* in Latimer & Giles (2018), Giles *et al*. (2021), and Ren & Xu (2021), or as the sister group of a larger Bear Gulch clade including *Aesopichthys*, *Proceramala*, *Wendyichthys*, and *Cyranorhis* by Cloutier & Arratia (2004). Argyriou *et al*. (2018) recovered a clade including *Kalops*, *Beagiascus*, *Coccocephalus*, *Boreosomus*, and *Luderia*, and this clade is sister to the *Pteronisculus* + *Aesopichthys* + *Cyranorhis* + *Wendyichthys* clade. Some other studies suggested that the

Bear Gulch fishes are not closely related to each other. *Kalops* clade is the sister group of *Elonichthys* + *Palaeoniscum* clade in Mickle *et al*. (2009). However, Mickle (2012) hypothesized that *Kalops* is closely related to *Gonatodus*. Both are nested in a clade without any other members from Bear Gulch (see Mickle, 2012, fig. 9, node 46). Xu *et al*. (2014a) proposed that *Kalops* is in a pectinated paraphyletic position, between *Boreosomus* and *Palaeoniscum*. In the phylogenetic result from Stack & Gottfried (2022), *Kalops* is in a polyphyletic position, along with some other early actinopterygians. In summary, although using different sets of characters, my result is more in agreement with those of Cloutier & Arratia (2004) than other studies.

Survivability. The new discovery of *Kalops loganensis* n. sp. extends the youngest age of this genus to nearly 13 million years from Surpukhovian to Moscovian (Heckel, 2008, 2013, 2022; Lund *et al*. 2015). Geographically, The Bear Gulch Limestone is part of the Williston Basin, and the Logan Quarry Shale is on the east side of the Illinois Basin (Grogan & Lund, 2002; Mastalerz *et al*. 2018). During the Mississippian these two basins were separated shallow marine systems, where the Illinois Basin is more on the east (see Over, 2021, fig. 1). The two shallow seas were connected as the subunits of the North American Midcontinent Sea (NAMS) during the Mid-Pennsylvanian stage. The Williston Basin formed the northeastern boundary of the NAMS, and the Illinois Basin, which is more than 500 km south of the Williston Basin, formed part of the southeastern boundary (Algeo & Heckel, 2008; Algeo *et al*. 2008).

In the NAMS, the repeated stratigraphy (transgression limestone, gray/black shales, regression limestone) indicates the cycles of eustatic fluctuations across the three North American Pennsylvanian regional stages, from Desmoinesian, Missurian to

Virgilian. These fluctuations were driven by the global ice age intervals during the Late Paleozoic and were defined as "cyclothems" (Heckel, 2008, 2013, 2023; Felding, 2021). So far, all the cyclothems are studied and named based on local geological units. The Logan Quarry Shale member is in the Upper Tiawah cyclothem, which is the 22nd cyclothem (Heckel, 2013; Rosscoe & Barrick, 2021).

Potential habitat. *Kalops* has a fusiform body shape, together with the location of the fins, indicating that this genus is a fast swimmer around the middle and top of the tropical shallow marine (Webb, 1984). Similar live styles can be found in the Triassic *Pteronisculus* (Xu *et al*. 2014b). This ecological niche of adapting to the top of the water might explain how this fish can survive through the cyclothems. They were able to follow the raise and retreat of the sea level, distributed from Williston Basin to Illinois Basin, and tolerate the cyclothems in the NAMS.

Conclusions

Studies of two specimens from the Logan Quarry reveal a new species of *Kalops*, *K. loganensis* n. sp. as the youngest representative of its genus in the Moscovian of North America. The morphological study and phylogenetic analysis confirm that the new species is the sister of the other two known *Kalops* species. The genus is interpreted as a stem Actinopterygii, closely related to other Bear Gulch fishes with fusiform body, such as *Beagiascus*, *Cyranorhis*, and *Wendyichthys*. Based on geological evidence, *Kalops* is the only actinopterygian genus occurring in both the Bear Gulch Limestone and a younger unit, Logan Quarry. Further studies can focus on the functional morphomatrix analysis to quantify the size and shape of *K. loganensis* n. sp. in regard to survivorship.

Another direction is the faunal dynamics analysis of how the cyclothems affect the marine vertebrate faunas in the NAMS.

Acknowledgements

Greatly thanks to H.-P. Schultze ang G. Arratia for the comments and suggestions on this manuscript, and J. Chorn for the suggestions on photographing. I also appreciate A. Stroup and W. Simpson for accessibility to the Field Museum specimens. This work is supported by 2020, 2021, and 2022 Research Grants of the Association of Earth Science Clubs of Greater Kansas City; 2021 BI Panorama Grant of the department of Ecology & Evolutionary Biology, University of Kansas; 2022 and 2023 EEB Summer Funding, University of Kansas. Their support is greatly appreciated.

References

Algeo TJ, Heckel PH. The Late Pennsylvanian midcontinent sea of North America: a review. Palaeogeography, Palaeoclimatology, Palaeoecology. 2008 Oct 24;268(3-4):205-21.

Algeo T, Rowe H, Hower JC, Schwark L, Herrmann A, Heckel P. Changes in ocean denitrification during Late Carboniferous glacial–interglacial cycles. Nature Geoscience. 2008 Oct;1(10):709-14.

Argyriou T, Giles S, Friedman M, Romano C, Kogan I, Sánchez-Villagra MR. Internal cranial anatomy of Early Triassic species of† Saurichthys (Actinopterygii:† Saurichthyiformes): implications for the phylogenetic placement of† saurichthyiforms. BMC Evolutionary Biology. 2018 Dec;18:1-41.

Arratia G. Actinopterygian postcranial skeleton with special reference to the diversity of fin ray elements, and the problem of identifying homologies. In: G. Arratia, H.-P. Schultze & M. V. H. Wilson (eds) Mesozoic Fishes 4 – Homology and Phylogeny. 2008;4:49-101. Verlag Dr. F. Pfeil, München.

Arratia G. Identifying patterns of diversity of the actinopterygian fulcra. Acta Zoologica. 2009 May;90:220-35.

Carpenter D, Falcon-Lang HJ, Benton MJ, Nelson WJ. Fishes and tetrapods in the Upper Pennsylvanian (Kasimovian) Cohn coal member of the Mattoon formation of Illinois, United States: systematics, paleoecology, and paleoenvironments. Palaios. 2011 Oct 1;26(10):639-57.

Cloutier R, Arratia G. Early diversification of actinopterygians. In: G. Arratia, M. V. H. Wilson, & R. Cloutier, Recent advances in the origin and early radiation of vertebrates. 2004:217-70. Verlag Dr. F. Pfeil, München.

Coates MI. Actinopterygians from the Namurian of Bearsden, Scotland, with comments on early actinopterygian neurocrania. Zoological Journal of the Linnean Society. 1998 Jan 1;122(1-2):27-59.

Cope, E. D. Zittel's manual of palaeontology. American Naturalist. 1887;21(11):1014-1019.

Coveney RM, Martin SP. Molybdenum and other heavy metals of the Mecca Quarry and Logan Quarry shales. Economic Geology. 1983 Feb 1;78(1):132-49.

Coveney Jr RM, Glascock MD. A review of the origins of metal-rich Pennsylvanian black shales, central USA, with an inferred role for basinal brines. Applied Geochemistry. 1989 Jul 1;4(4):347-67.

Elder RL, Smith GR. Fish taphonomy and paleoecology. Geobios. 1984 Jan 1;17:287-91.

Esin DN. The scale cover of *Amblypterina costata* (Eichwald) and the palaeoniscid taxonomy based on isolated scales. Paleontological Journal. 1990;2(9):90-98.

Falcon-Lang HJ. Pennsylvanian tropical rain forests responded to glacial-interglacial rhythms. Geology. 2004 Aug 1;32(8):689-692.

Falcon-Lang HJ. Small cordaitalean trees in a marine-influenced coastal habitat in the Pennsylvanian Joggins Formation, Nova Scotia. Journal of the Geological Society. 2005 May;162(3):485-500.

Falcon-Lang HJ, Benton MJ, Braddy SJ, Davies SJ. The Pennsylvanian tropical biome reconstructed from the joggins formation of Nova Scotia, Canada. Journal of the Geological Society. 2006 May;163(3):561-576.

Fielding CR. Late Palaeozoic cyclothems–A review of their stratigraphy and sedimentology. Earth-Science Reviews. 2021 Jun 1;217:103612.

Friedman M, Sallan LC. Five hundred million years of extinction and recovery: a Phanerozoic survey of large-scale diversity patterns in fishes. Palaeontology. 2012 Jul;55(4):707-742.

Giles S, Xu GH, Near TJ, Friedman M. Early members of ‘living fossil’lineage imply later origin of modern ray-finned fishes. Nature. 2017 Sep 14;549(7671):265-268.

Giles S, Feilich K, Warnock R, Pierce SE, Friedman M. High lineage survivorship across the end-Devonian Mass Extinction suggested by a remarkable new Late Devonian actinopterygian. bioRxiv. 2021 Sep 3:2021-09.

Grogan ED, Lund R. The geological and biological environment of the Bear Gulch Limestone (Mississippian of Montana, USA) and a model for its deposition. Geodiversitas. 2002;24(2):295-315.

Grogan ED, Lund R. A basal elasmobranch, *Thrinacoselache gracia* n. gen and sp.,(Thrinacodontidae, new family) from the Bear Gulch Limestone, Serpukhovian of Montana, USA. Journal of Vertebrate Paleontology. 2008 Dec 12;28(4):970-88.

Grogan ED, Lund R, Greenfest-Allen E. The origin and relationships of early chondrichthyans. Biology of sharks and their relatives. 2012 Apr 9;2:3-29.

Heckel PH. Thin widespread Pennsylvanian black shales of Midcontinent North America: a record of a cyclic succession of widespread pycnoclines in a fluctuating epeiric sea. Geological Society, London, Special Publications. 1991;58(1):259-273.

Heckel PH, Fielding CR, Frank TD, Isbell JL. Pennsylvanian cyclothems in Midcontinent North America as far-field effects of waxing and waning of Gondwana ice sheets. Resolving the late Paleozoic ice age in time and space: Geological Society of America Special Paper. 2008 Jan 1;441:275-289.

Heckel PH. Pennsylvanian stratigraphy of Northern Midcontinent Shelf and biostratigraphic correlation of cyclothems. Stratigraphy. 2013 Jan 1;10(1–2):3-9.

Heckel PH. North American Midcontinent Pennsylvanian cyclothems and their implications. Geological Society, London, Special Publications, 2022;535, https:// doi.org/10.1144/SP535-2022-182.

Huxley TH. On the application of the laws of evolution to the arrangement of the vertebrate and more particularly of the Mammalia. InProc Zool Soc London 1880 (Vol. 1880, pp. 649-662).

Kosanke RM, Simon JA, Wanless HR, Willman HB. Classification of the Pennsylvanian strata of Illinois. Report of investigations no. 214. 1960.

Latimer AE, Giles S. A giant dapediid from the Late Triassic of Switzerland and insights into neopterygian phylogeny. Royal Society Open Science. 2018 Aug 15;5(8):180497.

Lucas SG, Krainer KA, Allen BD, Barrick JE, Vachard D. The Carboniferous System in the Manzano Mountains, New Mexico. New Mexico Museum of Natural History and Science Bulletin. 2021;84:17-88.

Lucas SG, Schneider JW, Nikolaeva S, Wang X. The Carboniferous chronostratigraphic scale: history, status and prospectus. Geological Society, London, Special Publications. 2022;512(1):19-48.

Lund R. *Harpagofututor volsellorhinus* new genus and species (Chondrichthyes, Chondrenchelyiformes) from the Namurian Bear Gulch Limestone, Chondrenchelys

problematica Traquair (Visean), and their sexual dimorphism. Journal of Paleontology. 1982 Jul 1:938-58.

Lund R, Greenfest-Allen E, Grogan ED. Habitat and diversity of the Bear Gulch fish: life in a 318 million year old marine Mississippian bay. Palaeogeography, Palaeoclimatology, Palaeoecology. 2012 Jul 15;342:1-6.

Lund R, Greenfest-Allen E, Grogan ED. Ecomorphology of the Mississippian fishes of the Bear Gulch Limestone (Heath formation, Montana, USA). Environmental biology of fishes. 2015 Feb;98:739-54.

Lund R. The new actinopterygian order Guildayichthyiformes from the Lower Carboniferous of Montana (USA). Geodiversitas. 2000;22(2);171–206.

Harper D, Mastalerz M. Coal in Indiana: a geologic overview. Indiana Geological & Water Survey; 1998.

Mastalerz M, Eble C, Ames P, Drobniak A. Application of palynology and petrography in the correlation of the Pennsylvanian Brazil and Staunton Formation coals in the eastern part of the Illinois Basin. International Journal of Coal Geology. 2018 Apr 1;190:191-204.

Mickle KE. Unraveling the Systematics of Palaeoniscoid Fishes--Lower Actinopterygians in Need of a Complete Phylogenetic Revision (Doctoral dissertation, University of Kansas).

Mickle KE. Identification of the bones of the snout in fossil lower actinopterygians—a new nomenclature scheme based on characters. Copeia. 2015 Nov 1;103(4):838-857.

Mickle KE, Lund R, Grogan ED. Three new palaeoniscoid fishes from the Bear Gulch Limestone (Serpukhovian, Mississippian) of Montana (USA) and the relationships of lower actinopterygians. Geodiversitas. 2009 Sep;31(3):623-668.

Over DJ. The Devonian-Carboniferous boundary in the United States. Palaeobiodiversity and Palaeoenvironments. 2021 Jun;101(2):529-540.

Poplin CM. An actinopterygian with a long rostrum from the Pennsylvanian of Logan Quarry, Indiana. Journal of Paleontology. 1978 May 1:524-531.

Poplin CM. The dermosphenotic in early actinopterygians, a nomenclatural problem. In: G. Arratia & A. Tintori (eds). Mesozoic Fishes 3 Systematics, Paleoenvironments and Biodiversity. 2004;3:165-178. Verlag Dr. F. Pfeil, München.

Poplin CM, Lund R. Two new deep-bodied palaeoniscoid actinopterygians from Bear Gulch (Montana, USA, Lower Carboniferous). Journal of Vertebrate Paleontology. 2000 Sep 25;20(3):428-449.

Poplin CM, Lund R. Two Carboniferous fine-eyed palaeoniscoids (Pisces, Actinopterygii) from Bear Gulch (USA). Journal of Paleontology. 2002 Nov;76(6):1014-1028.

Ren Y, Xu GH. A new species of *Pteronisculus* from the Middle Triassic (Anisian) of Luoping, Yunnan, China, and phylogenetic relationships of early actinopterygian fishes. Vertebrata Palasiatica. 2021;59:169-199.

Rosscoe SJ, Barrick JE. Conodont faunas from the lower part of the Tinajas Member of the Atrasado Formation (Upper Pennsylvanian/Missourian), Manzanita Mountains, New Mexico. New Mexico Museum of Natural History and Science Bulletin. 2021;84:241-253.

Sallan LC, Kammer TW, Ausich WI, Cook LA. Persistent predator–prey dynamics revealed by mass extinction. Proceedings of the National Academy of Sciences. 2011 May 17;108(20):8335-8338.

Sallan LC, Friedman M. Heads or tails: staged diversification in vertebrate evolutionary radiations. Proceedings of the Royal Society B: Biological Sciences. 2012 May 22;279(1735):2025-2032.

Schneider JW, Lucas SG, Scholze F, Voigt S, Marchetti L, Klein H, Opluštil S, Werneburg R, Golubev VK, Barrick JE, Nemyrovska T. Late Paleozoic–early Mesozoic continental biostratigraphy—links to the standard global chronostratigraphic scale. Palaeoworld. 2020 Jun 1;29(2):186-238.

Schultze HP. Morphologische und histologische Untersuchungen an Schuppen mesozoischer Actinopterygier: Übergang v. Ganoid-zu Rundschuppen.[Mit] 61 Textabb. u. 5 Taf. 1966.126:232–312.

Schultze HP. The scales of Mesozoic actinopterygians. in G. Arratia & G. Viohl (eds) Mesozoic fishes – systematics and paleoecology. 1996;1:83-94. Verlag Dr. F. Pfeil, München.

Schultze HP, Arratia G, Wilson MV. Nomenclature and homologization of cranial bones in actinopterygians. In: G. Arratia, H.-P. Schultze, & M. V. H. Wilson (eds). Mesozoic fishes 4 –homology and phylogeny. 2008;4:23-48. Verlag Dr. F. Pfeil, München.

Schultze HP. Scales, enamel, cosmine, ganoine, and early osteichthyans. Comptes Rendus Palevol. 2016 Jan 1;15(1-2):83-102.

Stack J, Gottfried MD. A new, exceptionally well-preserved Permian actinopterygian fish from the Minnekahta Limestone of South Dakota, USA. Journal of Systematic Palaeontology. 2021 Sep 17;19(18):1271-1302.

Webb PW. Body form, locomotion and foraging in aquatic vertebrates. American zoologist. 1984 Feb 1;24(1):107-120.

Williams ME. The Cladodont level sharks of the Pennsylvanian black shales of central North America. Palaeontographica. Abteilung a, Paläozoologie, Stratigraphie. 1985;190(3-6):83-158.

Wood SP. Recent discoveries of Carboniferous fishes in Edinburgh. Scottish Journal of Geology. 1975 Nov;11(3):251-8.

Xu GH, Gao KQ, Finarelli JA. A revision of the Middle Triassic scanilepiform fish *Fukangichthys longidorsalis* from Xinjiang, China, with comments on the phylogeny of the Actinopteri. Journal of Vertebrate Paleontology. 2014a Jun 7;34(4):747-59.

Xu GH, Shen CC, Zhao LJ. *Pteronisculus nielseni* sp. nov., a new stem-actinopteran fish from the Middle Triassic of Luoping, Yunnan Province, China. Vertebrata PalAsiatica. 2014b Dec 15;52(4):364.

Zangerl R. A new shark of the family Edestidae, *Ornithoprion hertwigi*, from the Pennsylvanian Mecca and Logan quarry shales of Indiana. Publication (Field Museum of Natural History), 1004. 1966:1-43.

Zangerl, R., Richardson, ES. The paleoecological history of two Pennsylvanian black shales. Fieldiana: Geology Memoirs, 1963;4: 1–352.

Zangerl R, Case GR. Iniopterygia: a new order of Chondrichthyan fishes from the Pennsylvanian of North America. Fieldiana: Geology Memoirs, 1973;6: 1–67.

Zangerl R., Case GR. *Cobelodus aculeatus* (Cope) an anacanthous shark from Pennsylvanian black shales of North America. Palaeontographica Abteilung A. 1976;154:107–157.

Click here to access/download;Figure;Figure 1.tif

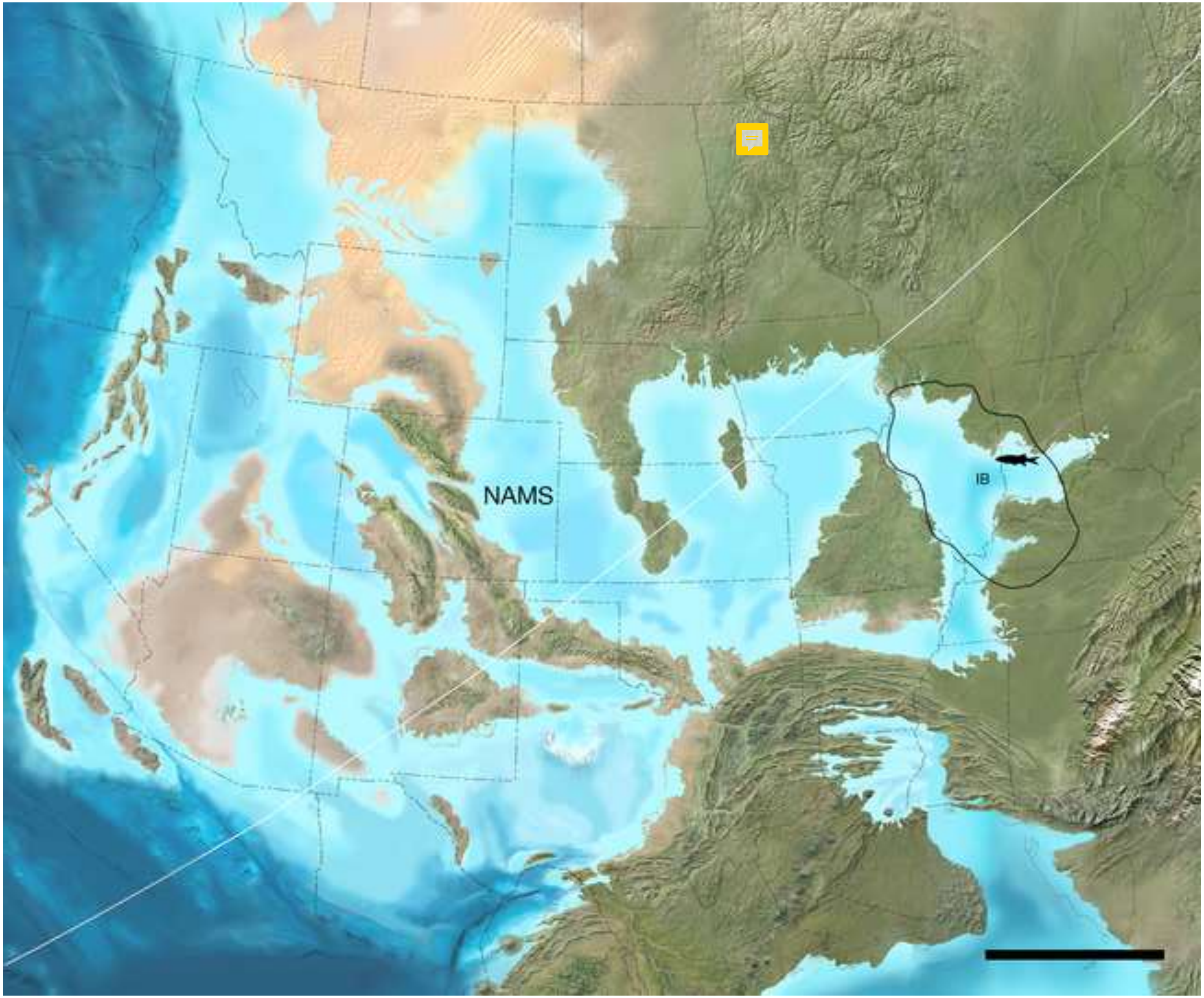

Click here to access/download;Figure;Figure 2.tif

<table>
<tr><th colspan="3">SGCS</th><th>Western Europe</th><th>USA</th></tr>
<tr><td>sub system</td><td>series</td><td>stage</td><td rowspan="2">stage</td><td>stage</td></tr>
<tr><td rowspan="11">Pennsylvanian</td><td rowspan="5">Upper</td><td rowspan="3">Gzhelian</td><td rowspan="2">Virgilian</td></tr>
<tr><td rowspan="5">Stephanian</td></tr>
<tr><td rowspan="2">Missourian</td></tr>
<tr><td rowspan="2">Kasimovian</td></tr>
<tr><td rowspan="3">Desmoinesian</td></tr>
<tr><td rowspan="3">Middle</td><td rowspan="3">Moscovian</td></tr>
<tr><td rowspan="3">Westphalian</td></tr>
<tr><td rowspan="3">Atokan</td></tr>
<tr><td rowspan="3">Lower</td><td rowspan="3">Bashkirian</td></tr>
<tr><td rowspan="2">Namurian</td></tr>
<tr><td>Morrowan</td></tr>
</table>

stage \ state	IN	KS	KS	IL	NM
Desmoinesian		Marmaton Gr.		St. David m.	Gray Mesa Fm.
	Houchin Creek m.			Hanover Limestone m.	
	Excello Shale m.				
		Cherokee Gr.			
	Coal IV (Survant)		Bevier Fm.	Purington Shale m.	
	Mecca Quarry Shale m.		Verdigris Fm.		
				Francis Creek Shale m.	
	Coal IIIa (Colchester)			Colchester m.	
	Coal III (Seeleville)		Robinson Branch Fm.		
	Logan Quarry Shale m.		Tebo Fm.	Vergennes Shale m.	

Click here to access/download;Figure;Figure 4.tif

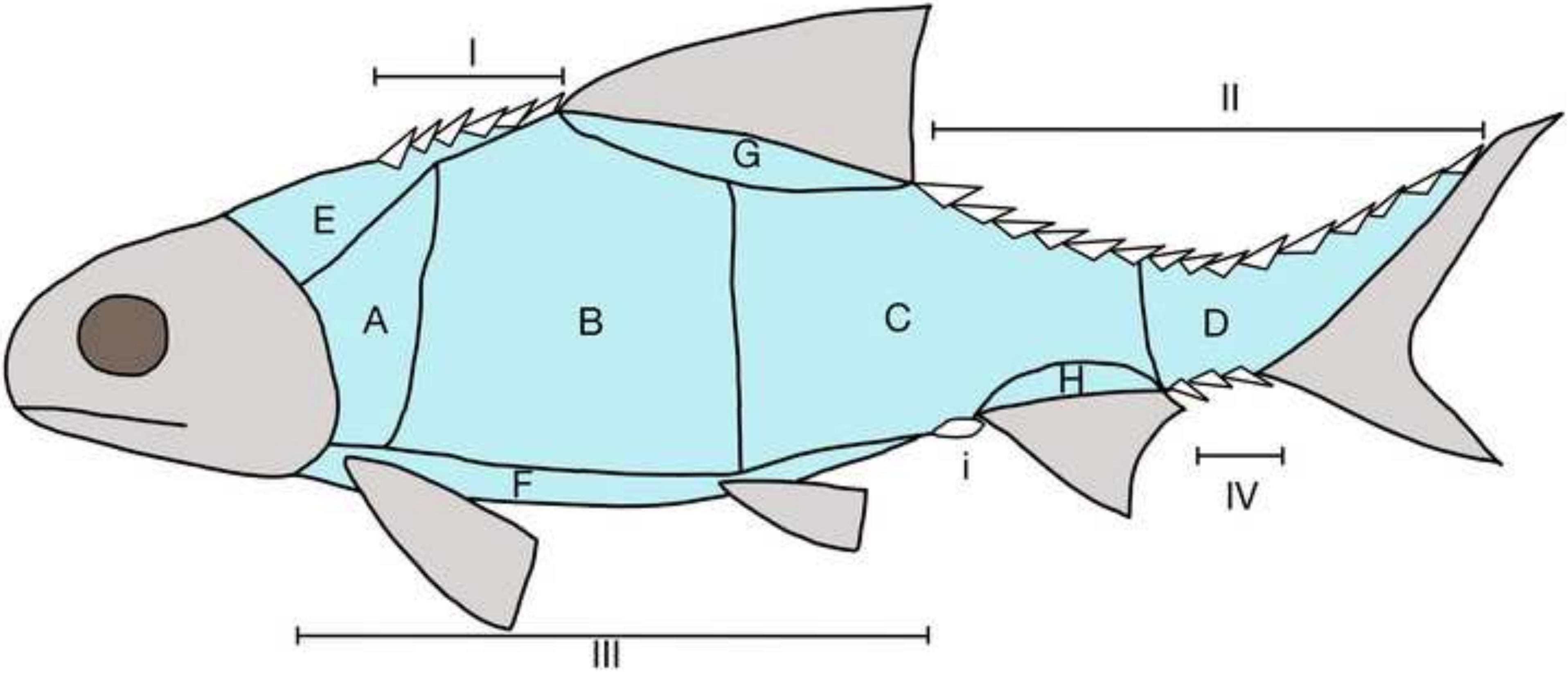

Click here to access/download;Figure;Figure 5.tif

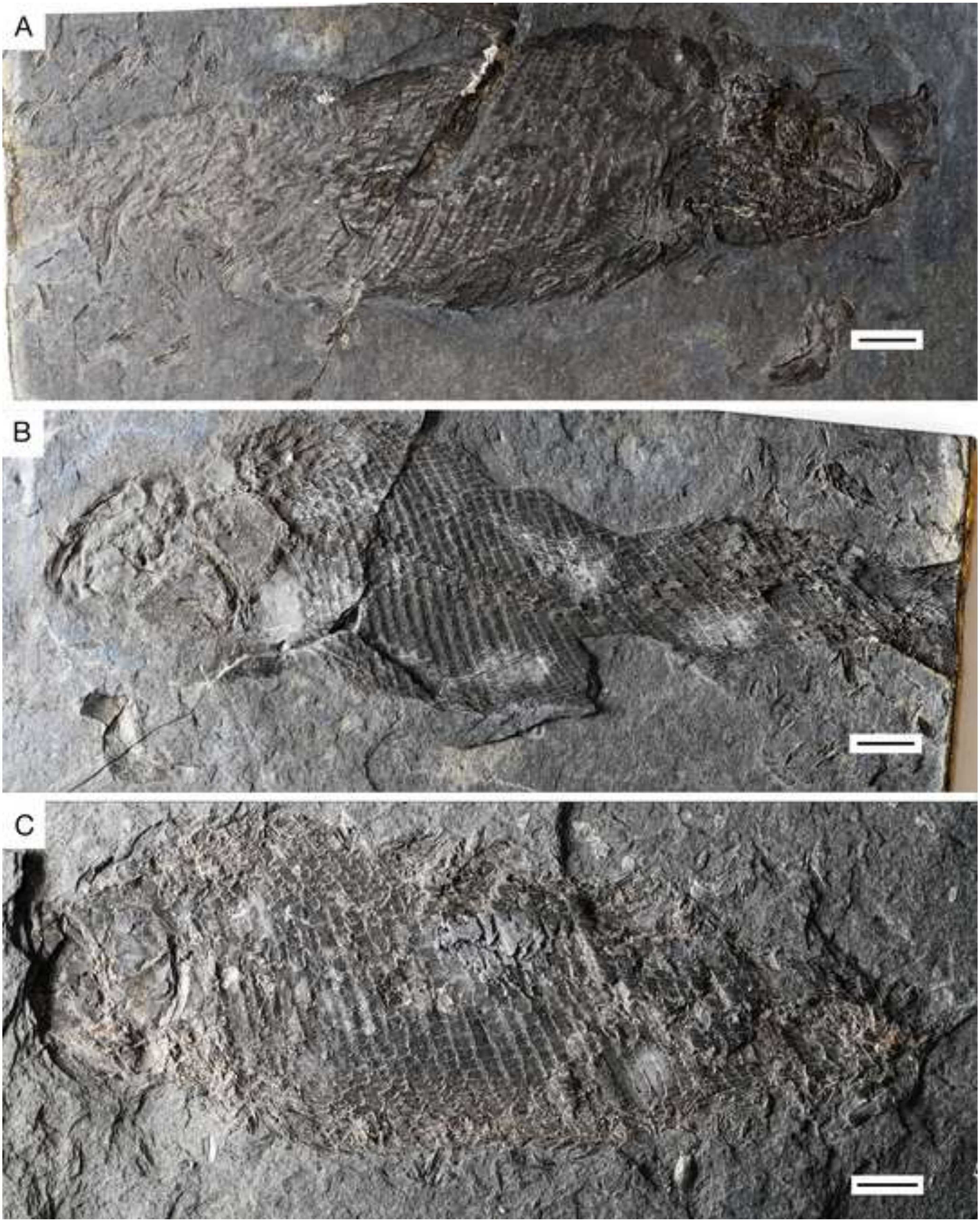

Click here to access/download;Figure;Figure 6.tif

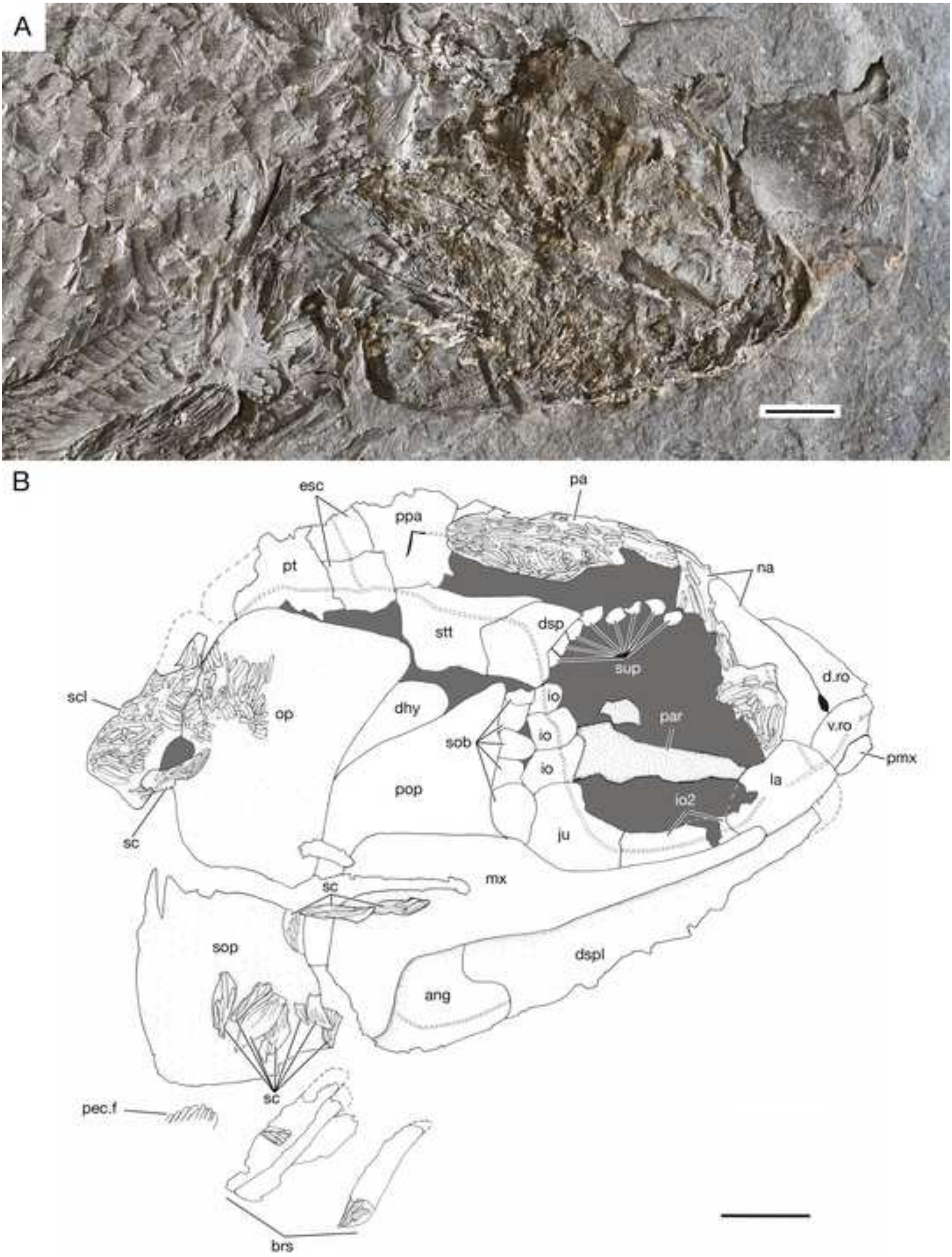

Click here to access/download;Figure;Figure 7.tif

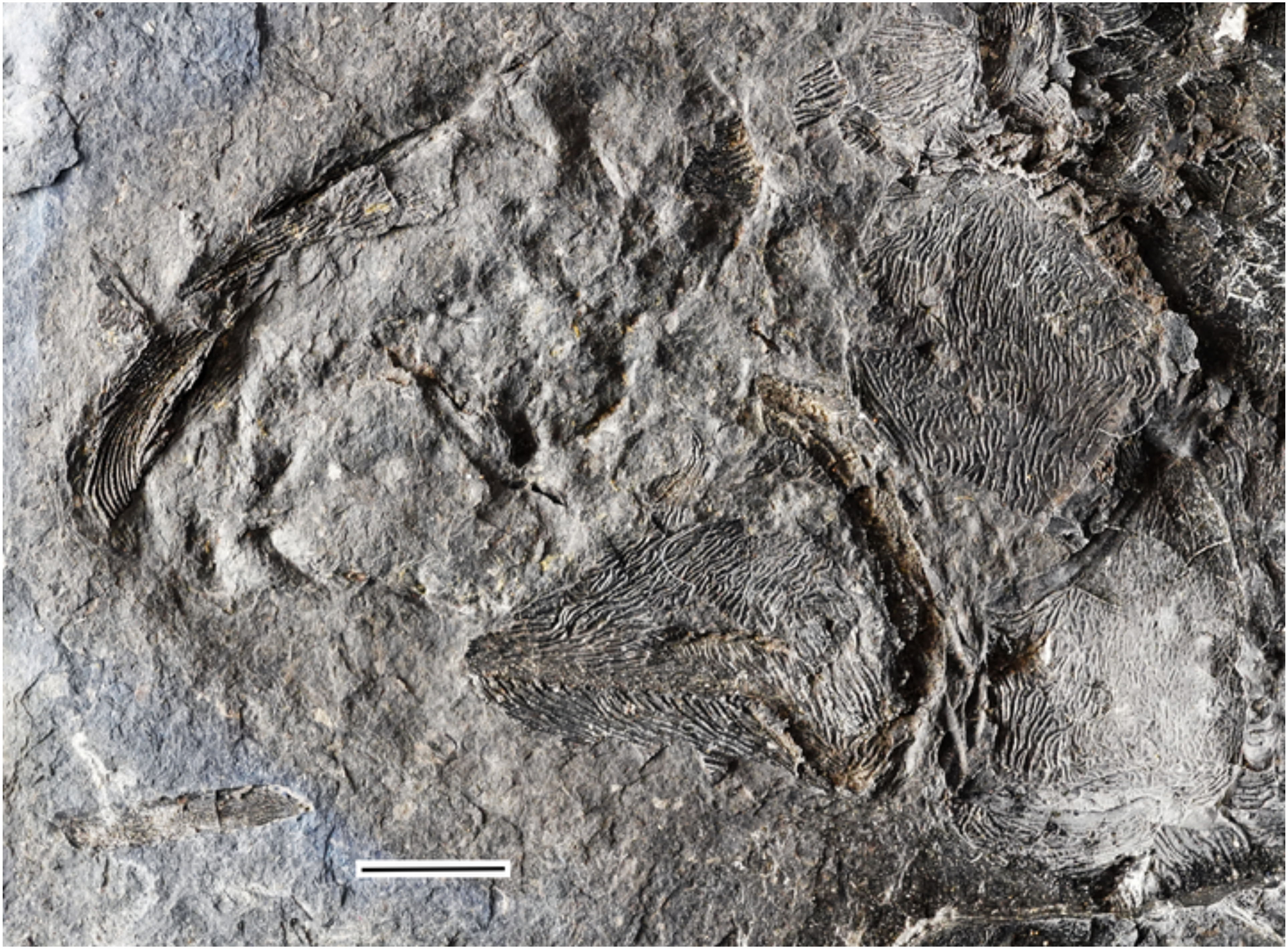

Click here to access/download;Figure;Figure 8.tif

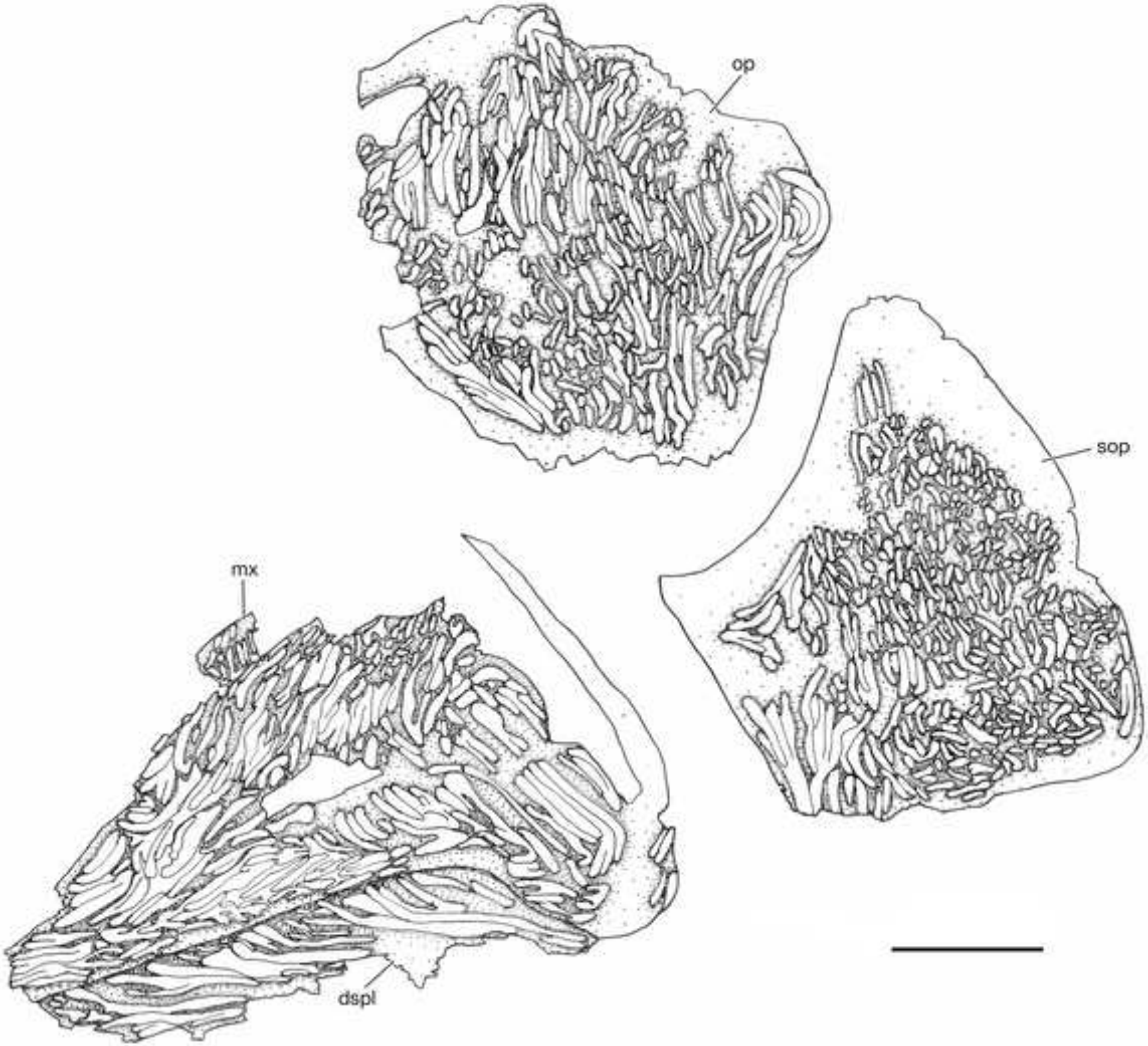

Click here to access/download;Figure;Figure 9.tif

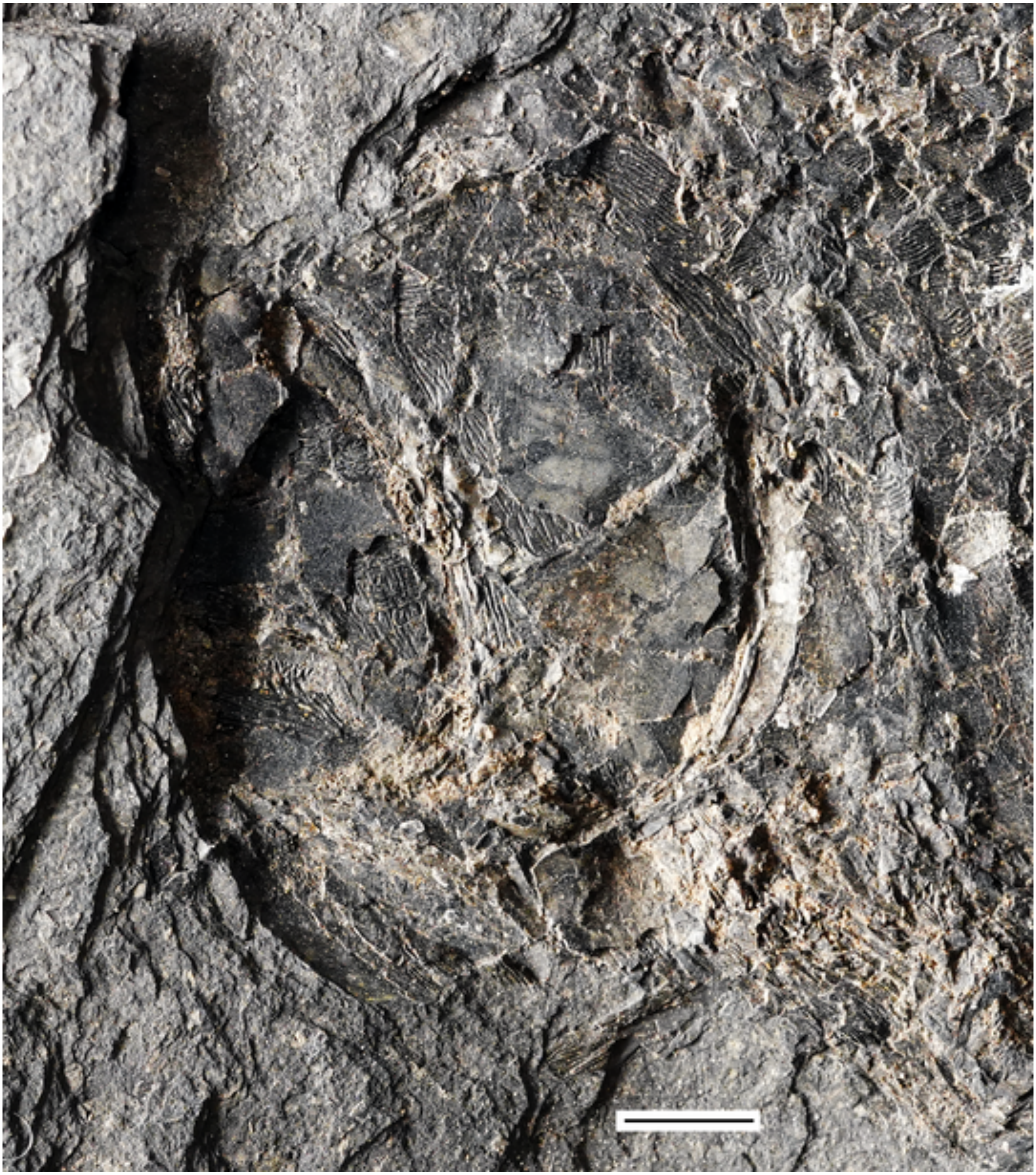

Click here to access/download;Figure;Figure 10.tif

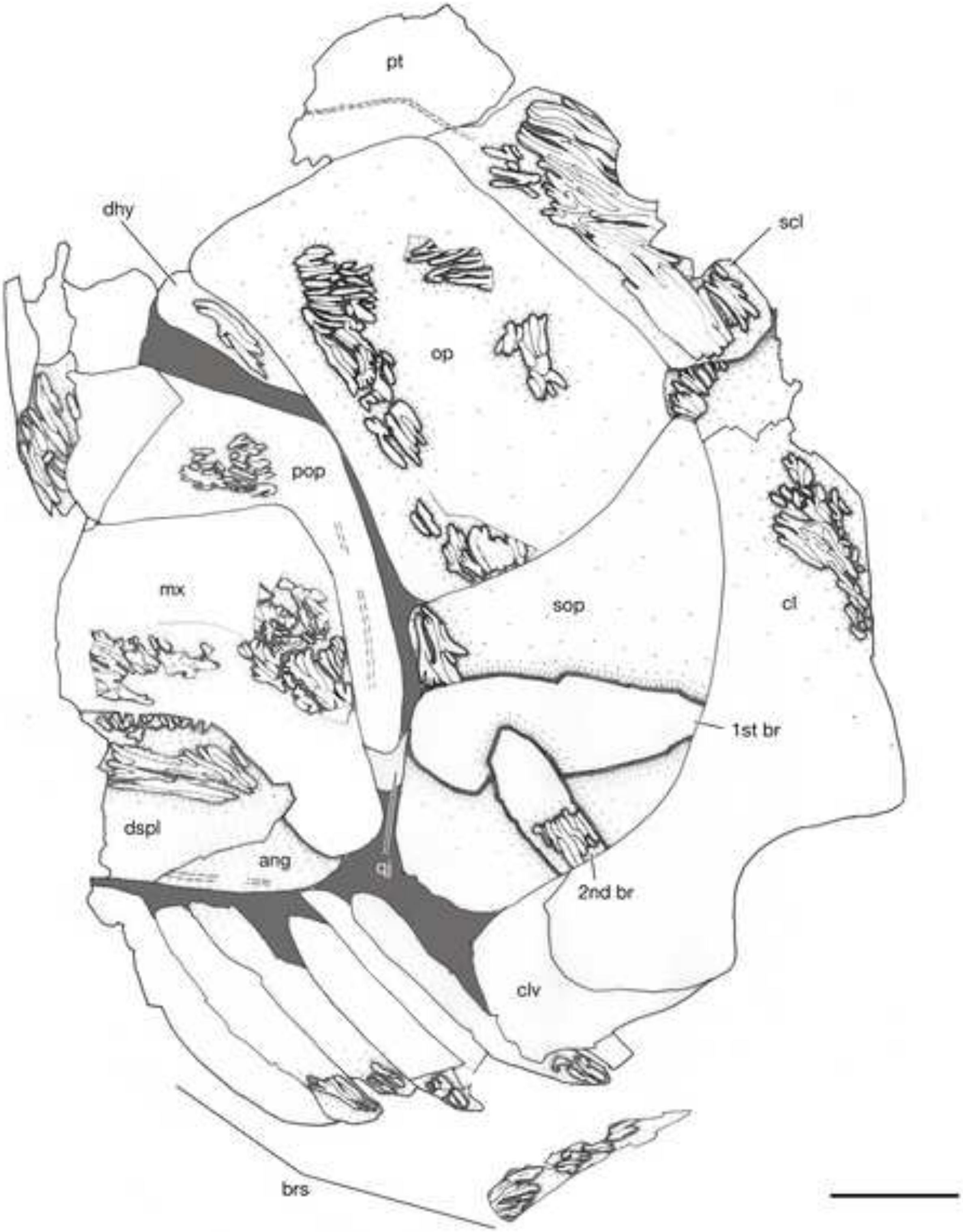

Click here to access/download;Figure;Figure 11.tif

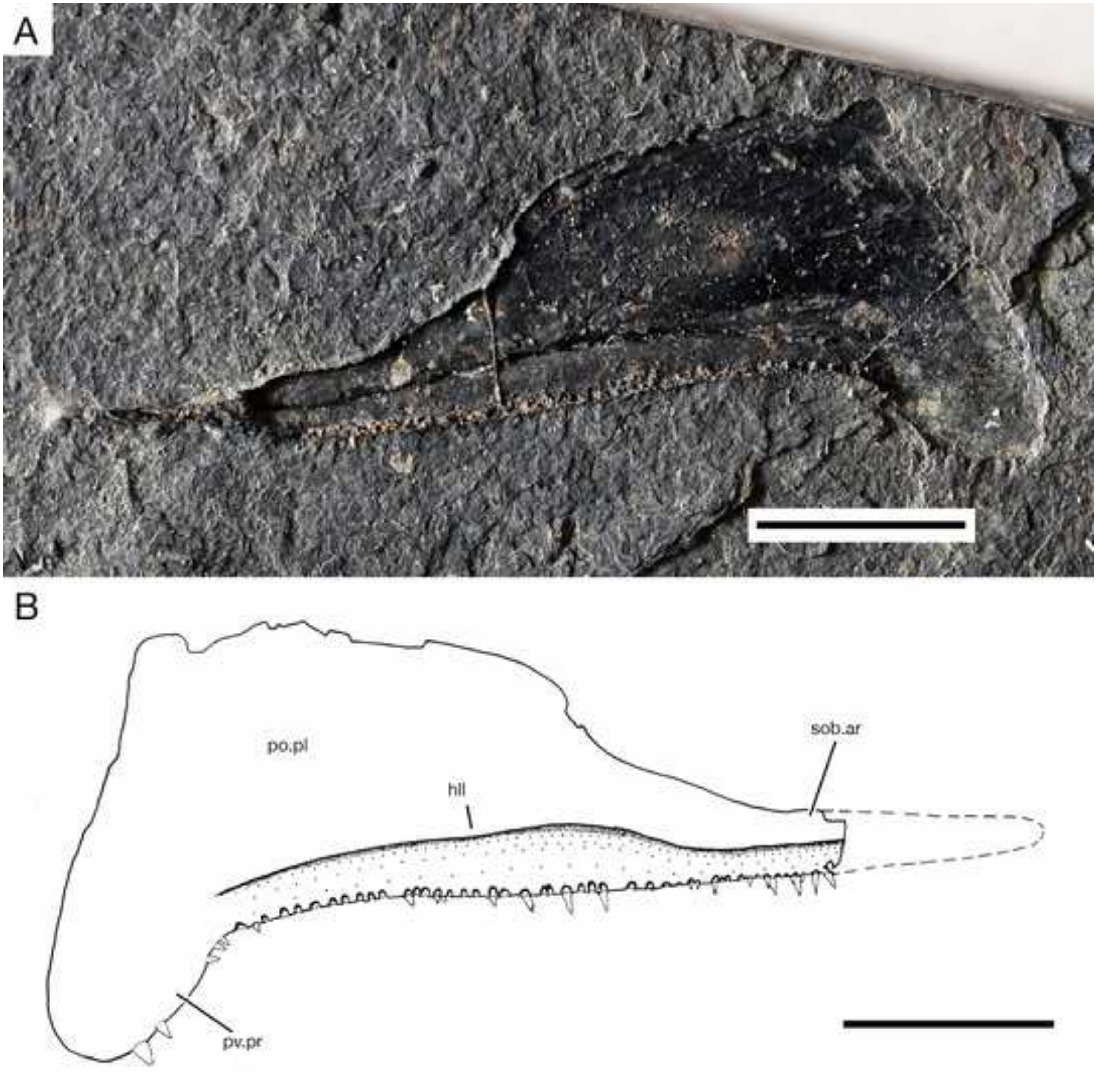

Click here to access/download;Figure;Figure 12.tif

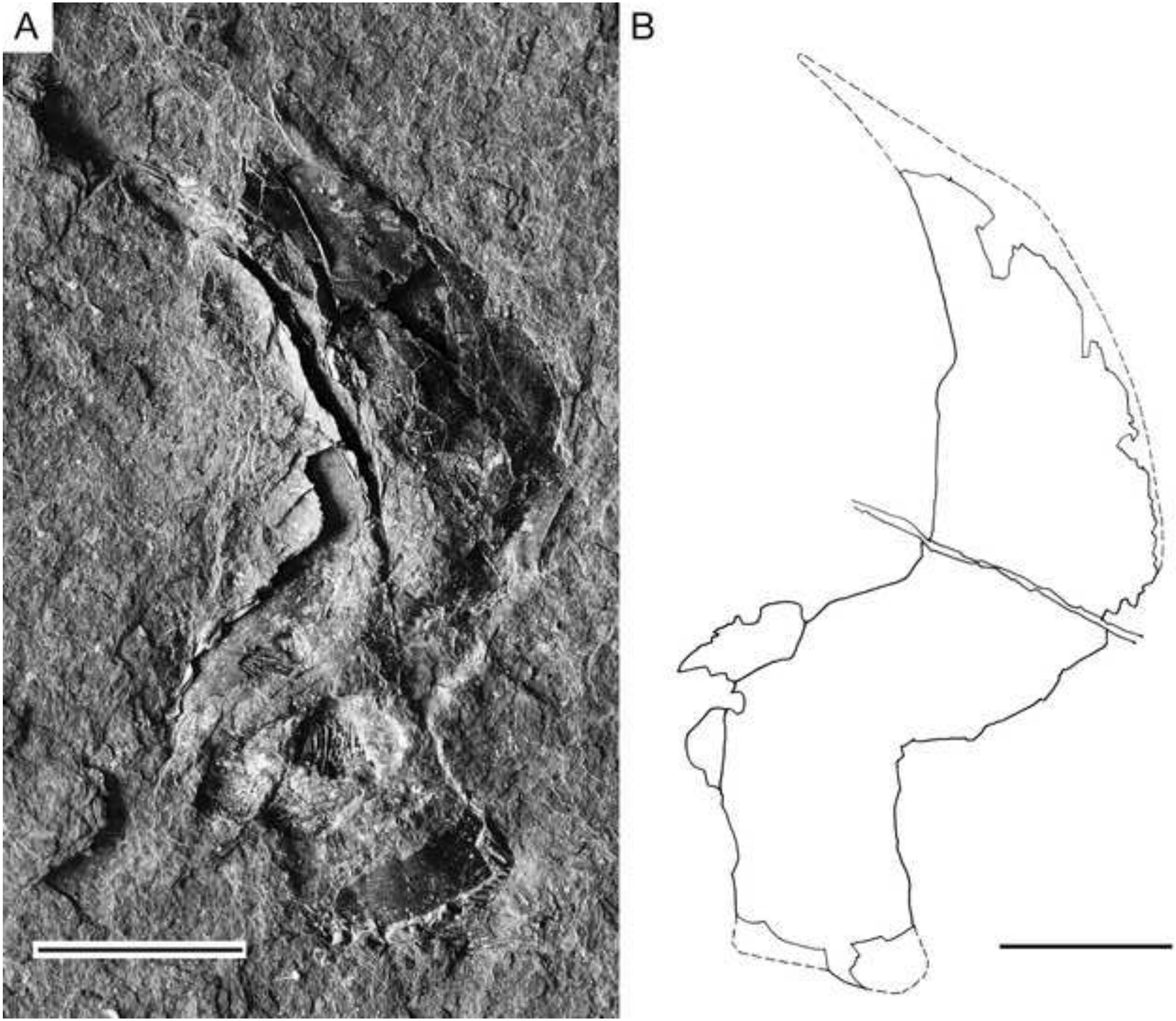

Click here to access/download;Figure;Figure 13.tif

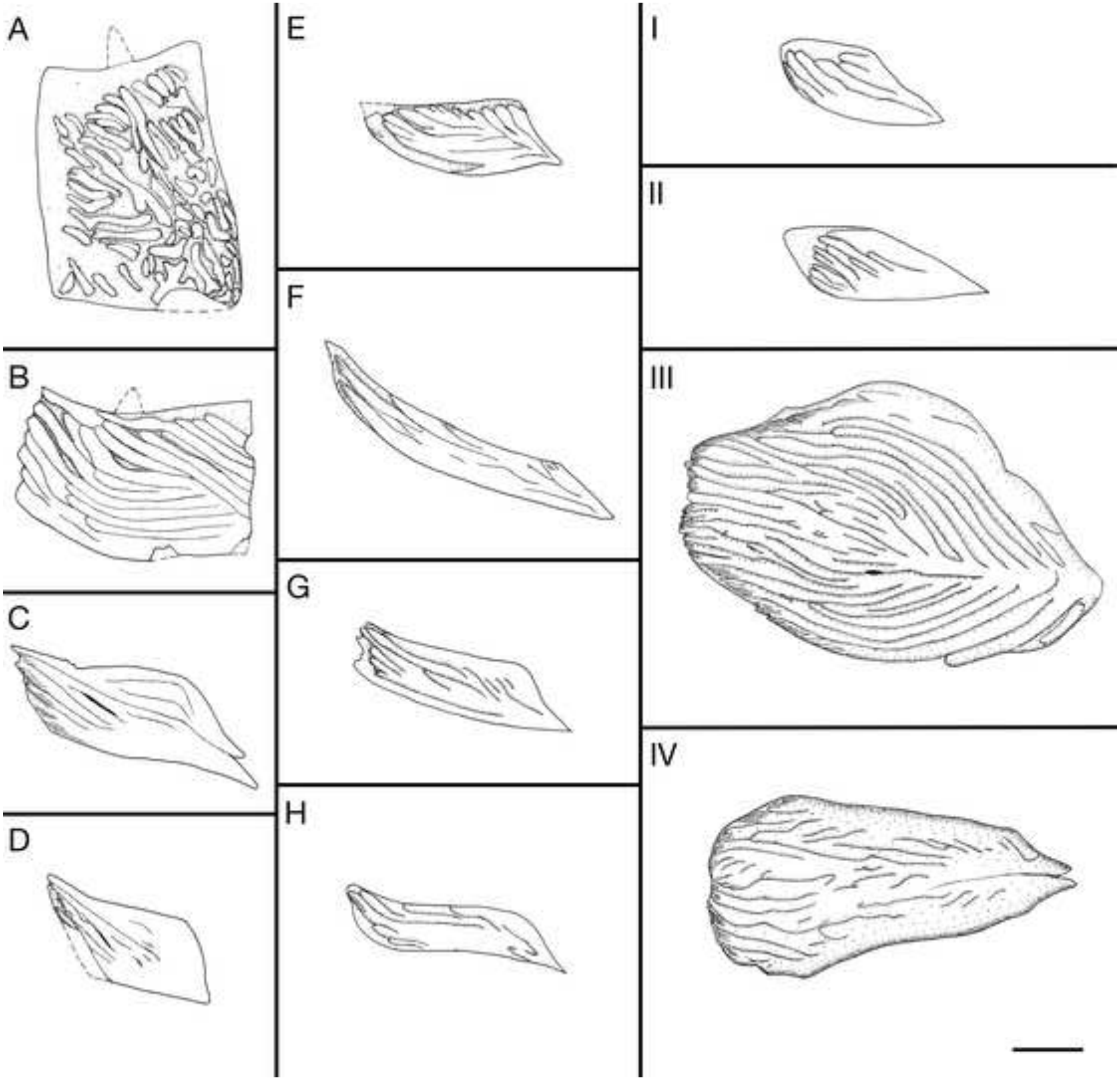

Click here to access/download;Figure;Figure 14.tif

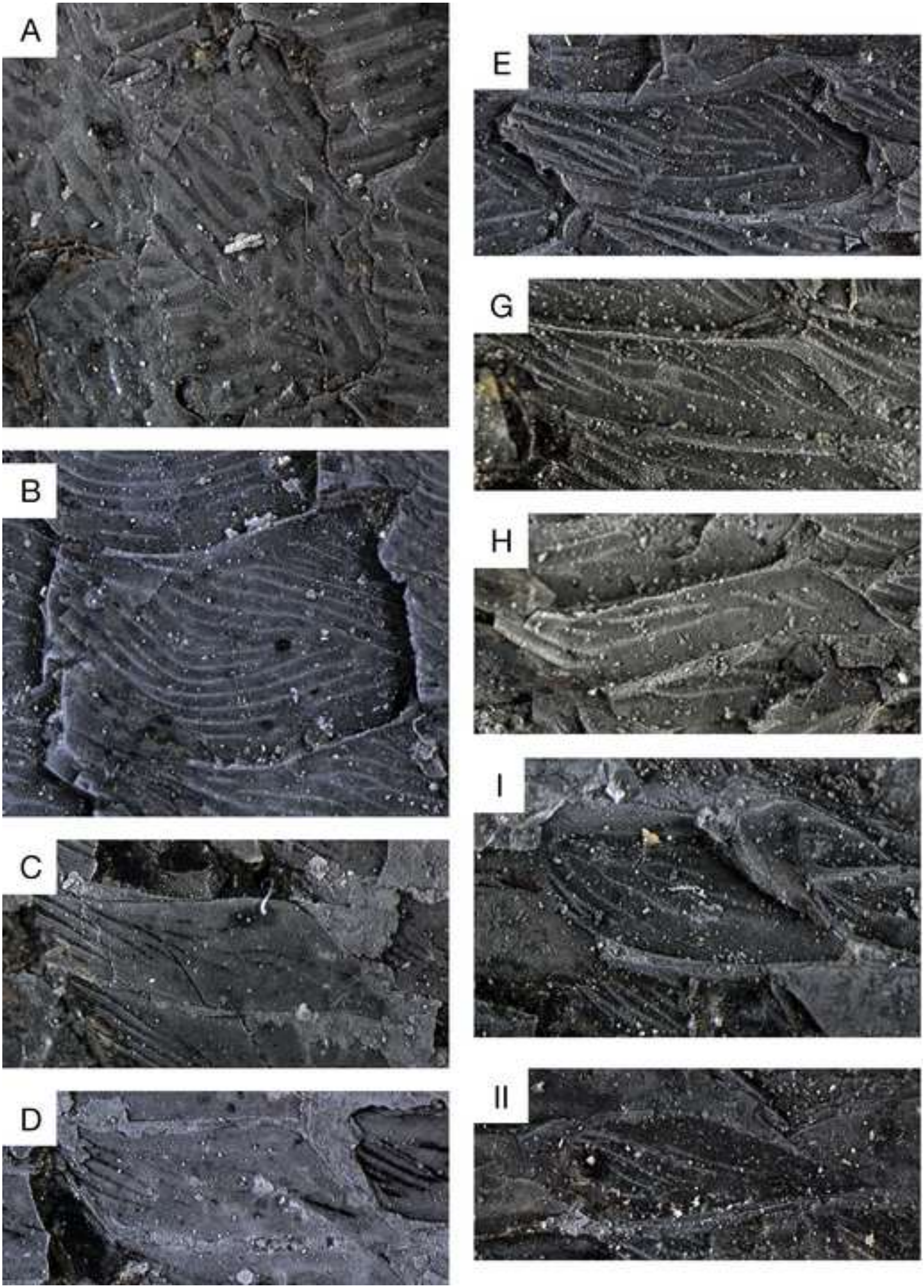

Click here to access/download;Figure;Figure 15.tif

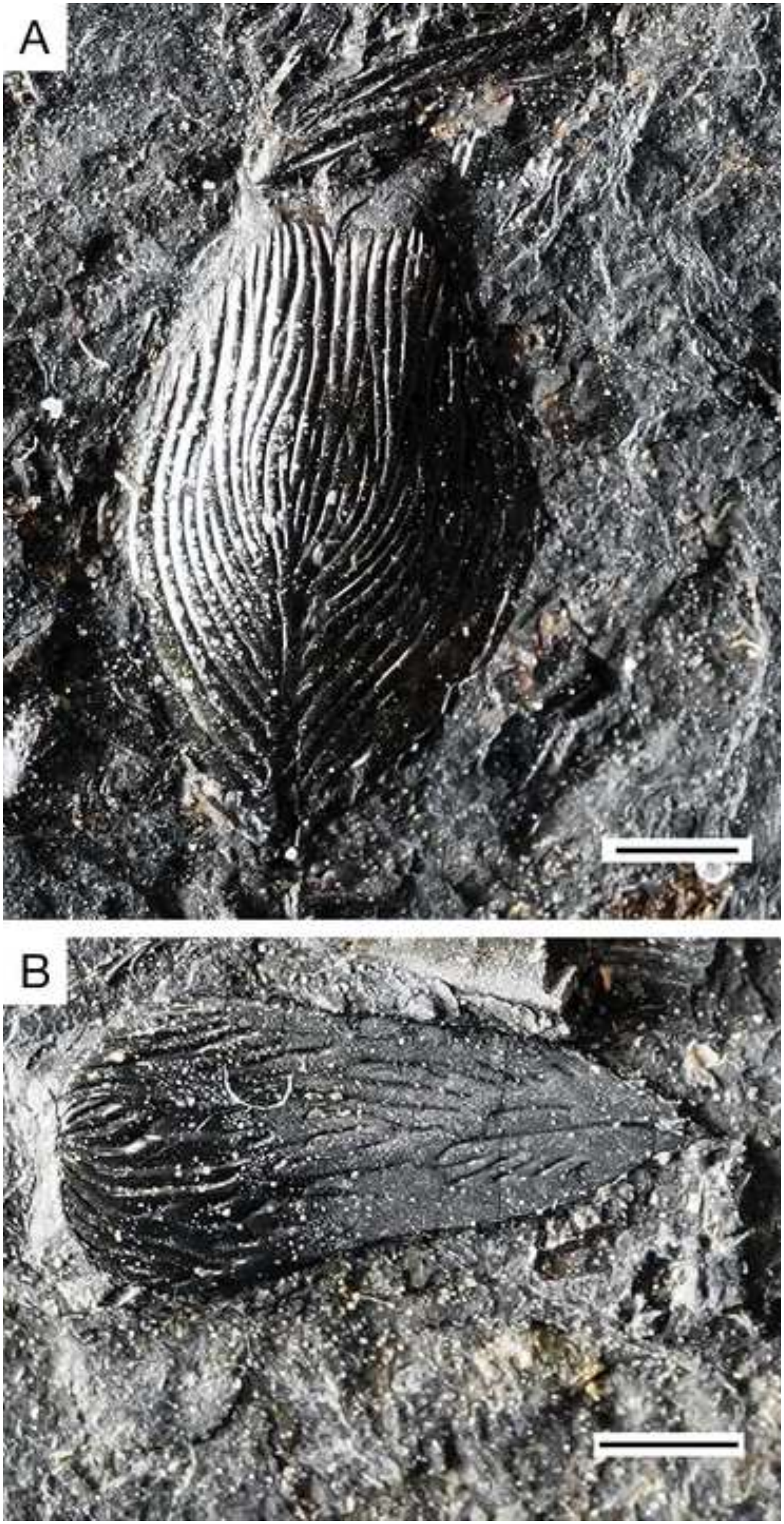

Click here to access/download;Figure;Figure 16.tif

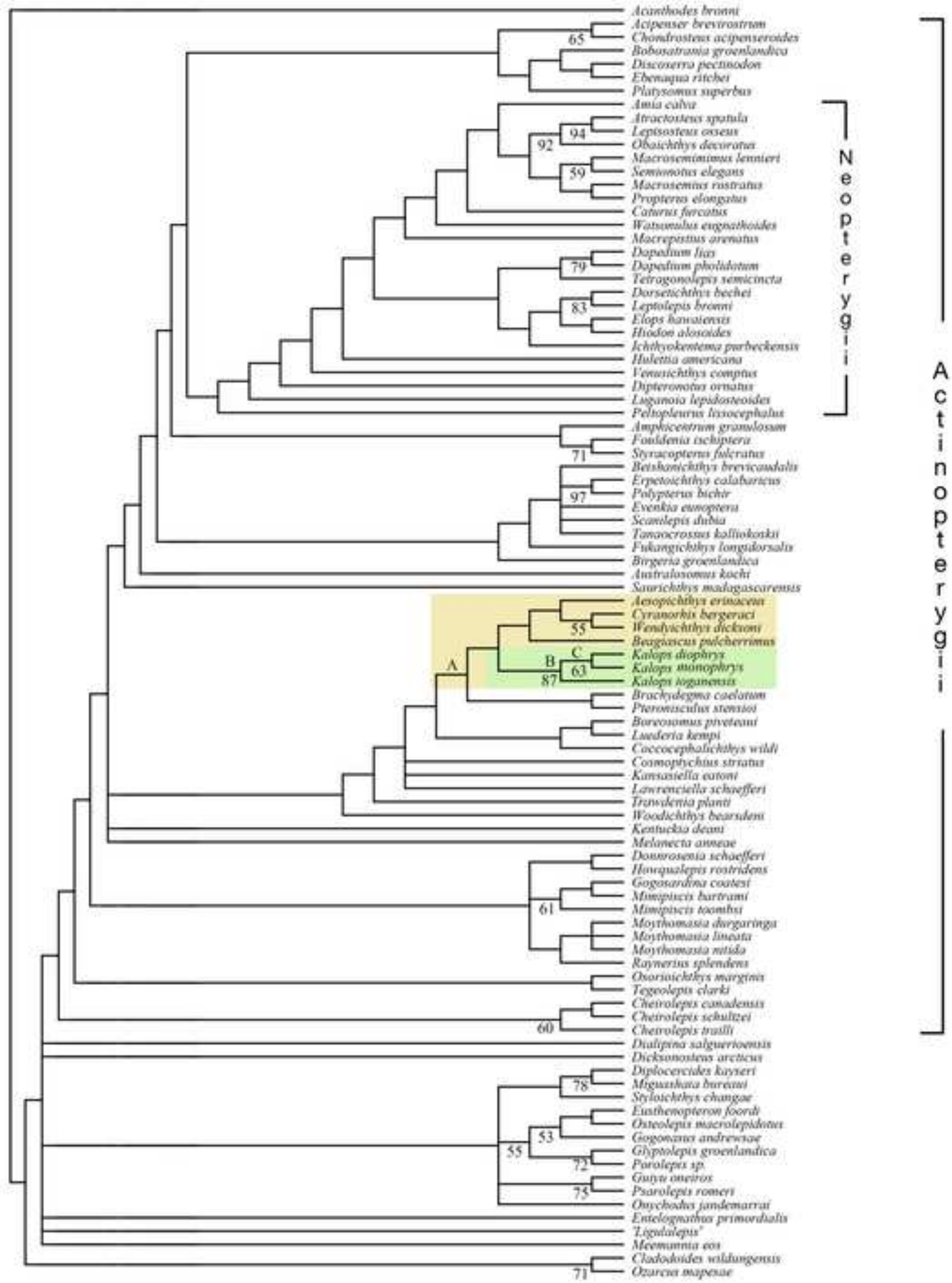

Click here to access/download
Supporting Information
Suppl Data.nex
