# Supplementary material for: A new early actinopterygian from the Mid-Pennsylvanian Logan Quarry Shale member of Indiana
Source: PLoS One. 2025 May 7;20(5):e0320932. doi: 10.1371/journal.pone.0320932 (PMC12057928; doi:10.1371/journal.pone.0320932)
Supplement: Attachment — Submitted filename: BronsonComments_PLOS_Kalops.docx [file pone.0320932.s006.docx]

Comments to the author

This is an interesting description of a new species of *Kalops*, with lovely camera lucida illustrations. However, despite the detailed descriptive section, the paper needs to be heavily edited before it can be a useful contribution. As currently written, the text is difficult to understand, and there are numerous typos that further obscure clarity and make me a little concerned about the time and attention to detail invested in the manuscript.

The section reporting results of the phylogenetic analysis also contains concerning statements regarding homoplasy. The author does not seem to be clear on the meaning of a synapomorphy vs. a homoplastic trait.

The section on potential habitat and on stratigraphy is underdeveloped. Can the author discuss perhaps the speciation of *Kalops* given that we now have this species in addition to the Bear Gulch material, in context of the depositional environment and the ages of both deposits? I think the author means to say it’s an epipelagic fish, though the language is unclear, and if it is epipelagic, that says something about its distribution as well as its potential survival through cyclothems.

In general, I think the first few Figures are a little bit under-designed – they would benefit from better mapping of the extent of IB & NAMS. The camera lucida work is beautiful but needs to be labeled in more detail for easy digestion by a reader. Lastly, the phylogeny figure’s OK, but the authors should explain where support values came from, and which support values are hidden in the figure caption.

I have marked numerous comments in the attached PDF using ‘sticky notes,’ so please let me know if those aren’t showing up! Due to time constraints, I didn’t check through the references or re-run the phylogenetic analysis, but the Nexus file of the character matrix looked OK to me when I skimmed it over. I didn’t have time to confirm character codings or check deeply for miscodings or similar issues.

Comments to the editor

As a mostly-chondricthyan paleoichthyologist, I recommend the paper be sent out for additional review perhaps by Sam Giles or Alan Pradel, who both have more experience with paleoniscoids.
