# Supplementary material for: A new early actinopterygian from the Mid-Pennsylvanian Logan Quarry Shale member of Indiana
Source: PLoS One. 2025 May 7;20(5):e0320932. doi: 10.1371/journal.pone.0320932 (PMC12057928; doi:10.1371/journal.pone.0320932)
Supplement: Attachment — Submitted filename: Response to Reviewers.docx [file pone.0320932.s007.docx]

Dear Editor,

I would like to thank the reviewers for the time and efforts on reviewing my manuscript. I include them in the Acknowledgement section in the manuscript.

All my answers to the journal requirements, and to the comments from the reviewers are highlighted in red.

I hope that you will be satisfied with the revised manuscript and it is to be considered for acceptance in the PLOS One journal.

Best regards,

Chenchen Shen

**Response to the journal requirements:**

1. When submitting your revision, we need you to address these additional requirements.

Answer: The format of this manuscript is edited and it is following the instructions of PLOS One.

2. In your manuscript, please provide additional information regarding the specimens used in your study. Ensure that you have reported human remain specimen numbers and complete repository information, including museum name and geographic location.

 Answer: The sentence below is added at the end of conclusions section:

'No permits were required for the described study, which complied with all relevant regulations.'

3. Thank you for stating the following financial disclosure:

 [2020, 2021, and 2022 Research Grants of the Association of Earth Science Clubs of Greater Kansas City (for students…..);

2021 BI Panorama Grant of the Department of Ecology & Evolutionary Biology, University of Kansas (student competition);

 2022 and 2023 EEB Summer Funding for graduate students, University of Kansas. ].

Please state what role the funders took in the study.  If the funders had no role, please state: ""The funders had no role in study design, data collection and analysis, decision to publish, or preparation of the manuscript. The funding was for support of my student PhD dissertation""

If this statement is not correct you must amend it as needed.

Please include this amended Role of Funder statement in your cover letter; we will change the online submission form on your behalf.

Answer: the funding information is now removed from the acknowledgement section. I added them in the cover letter file.

4. Thank you for stating the following in the Acknowledgments Section of your manuscript:

[Greatly thanks to H.-P. Schultze ang G. Arratia for the comments and suggestions on this manuscript, and J. Chorn for the suggestions on photographing. I also appreciate A. Stroup and W. Simpson for accessibility to the Field Museum specimens. This work is supported by 2020, 2021, and 2022 Research Grants of the Association of Earth Science Clubs of Greater Kansas City; 2021 BI Panorama Grant of the Department of Ecology & Evolutionary Biology, University of Kansas; and 2022 and 2023 EEB Summer Funding, University of Kansas for research associated to my PhD dissertation. Their support is greatly appreciated.]

We note that you have provided funding information that is not currently declared in your Funding Statement. However, funding information should not appear in the Acknowledgments section or other areas of your manuscript. We will only publish funding information present in the Funding Statement section of the online submission form.

Please remove any funding-related text from the manuscript and let us know how you would like to update your Funding Statement. Currently, your Funding Statement reads as follows:

  [2020, 2021, and 2022 Research Grants of the Association of Earth Science Clubs of Greater Kansas City;

2021 BI Panorama Grant of the department of Ecology & Evolutionary Biology, University of Kansas;

 2022 and 2023 EEB Summer Funding, University of Kansas. ].

Answer: same answer as above.

5. We note that Figure 1 in your submission contain [map/satellite] images which may be copyrighted. All PLOS content is published under the Creative Commons Attribution License (CC BY 4.0), which means that the manuscript, images, and Supporting Information files will be freely available online, and any third party is permitted to access, download, copy, distribute, and use these materials in any way, even commercially, with proper attribution. For these reasons, we cannot publish previously copyrighted maps or satellite images created using proprietary data, such as Google software (Google Maps, Street View, and Earth). For more information, see our copyright guidelines: http://journals.plos.org/plosone/s/licenses-and-copyright.

We require you to either (1) present written permission from the copyright holder to publish these figures specifically under the CC BY 4.0 license, or (2) remove the figures from your submission:

Answer: I bought this map with permission to publish from Colorado Plateau Geosystems Inc. I submitted the license of this map with other files. The file name is: Lic70423AcademShen.pdf.

6. We note that Figure(s) 5, 6, 7, 9, 11, 12, 14 and 15 in your submission contain copyrighted images. All PLOS content is published under the Creative Commons Attribution License (CC BY 4.0), which means that the manuscript, images, and Supporting Information files will be freely available online, and any third party is permitted to access, download, copy, distribute, and use these materials in any way, even commercially, with proper attribution. For more information, see our copyright guidelines: http://journals.plos.org/plosone/s/licenses-and-copyright.

We require you to either (1) present written permission from the copyright holder to publish these figures specifically under the CC BY 4.0 license, or (2) remove the figures from your submission:

Answer: For the figures mentioned above, there is no need for permissions because I took all the photographs on my own. I also added a sentence in the materials and methods section to explain this issue.

7. We are unable to open your Supporting Information file [Suppl Data.nex]. Please kindly revise as necessary and re-upload.

Answer: Now the file name is: S2_File.nex. It could be opened in Mesquite.

8. Please take this opportunity to be sure you have met all of our guidelines for new species. For proper registration of a new zoological taxon, we require two specific statements to be included in your manuscript.

1. In the Results section, the globally unique identifier (GUID), currently in the form of a Life Science Identifier (LSID), should be listed under the new species name, for example:

Anochetus boltoni Fisher sp. nov. urn:lsid:zoobank.org:act:B6C072CF-1CA6-40C7-8396-534E91EF7FBB

Another LSID for the manuscript itself should also appear within the Nomenclature statement. You will need to contact Zoobank (zoobank.org/About) to obtain a GUID (LSID). You should receive one LSID for your manuscript and a separate, unique LSID for the new species.

2. Please also insert the following text into the Methods section, in a sub-section to be called ""Nomenclatural Acts"":

The electronic edition of this article conforms to the requirements of the amended International Code of Zoological Nomenclature, and hence the new names contained herein are available under that Code from the electronic edition of this article. This published work and the nomenclatural acts it contains have been registered in ZooBank, the online registration system for the ICZN. The ZooBank LSIDs (Life Science Identifiers) can be resolved and the associated information viewed through any standard web browser by appending the LSID to the prefix ""http://zoobank.org/"". The LSID for this publication is: urn:lsid:zoobank.org:pub: XXXXXXX. The electronic edition of this work was published in a journal with an ISSN, and has been archived and is available from the following digital repositories: PubMed Central, LOCKSS [author to insert any additional repositories].

All PLOS ONE articles are deposited in PubMed Central and LOCKSS. If your institute, or those of your co-authors, has its own repository, we recommend that you also deposit the published online article there and include the name in your article.

Following a recent ruling by the International Commission on Zoological Nomenclature, electronic journals are now a valid format for publication of new zoological taxa. In order to ensure the valid publication of your new species, please be sure to include the updated version of Nomenclatural Acts (above). A complete explanation of our guidelines for publishing new species can be found on our website: <http://www.plosone.org/static/guidelines#zoological>.

Answer: the “Nomenclatural Acts” section is added to the Materials and methods section.

**Response to the comments from Reviewer 1:**

Line 18: … distinguished from rather than characterized among …

Answer: I replaced the words following this comment.

Line 31: You may want to consider using secondary headings in the Introduction section to organize the different information presented here.

Answer: I added two secondary headings (“Taxonomic history of *Kalops*” and “Geology and paleoenvironment of Black Shales”) to the introduction section.

Line 39: et al? Greenfest?

Answer: I corrected this citation as “Lund et al. 2015”, now all the citations are following the instruction of the journal.

Line 39: vertebrates

Answer: I corrected the typo.

Line 41: unmatched

Answer: The word was replaced.

Line 42: Check to see if it is better to describe this as estimated to be 141 species. I am not sure if all of the taxa included in the 2012 study have been officially described

Answer: I rephrased this sentence based on the comment, and confirmed that there are 141 studied species.

Line 42: I would reword this to make it easier to read. To date, there are 141 vertebrate species (55 Osteichthyes and 84 Chondrichthyes) recovered...

Answer: I rephrased this sentence based on the comment.

Line 44: Wider than who? The Osteichthyes?

Add the comparison in.

Answer: I rephrased this sentence based on the comment. Now it reads: Among the vertebrates, the chondrichthyan species identified from Bear Gulch have also been identified from additional coeval localities.

Line 49: I suggest a new paragraph here and then dive deeper into *Kalops* as a genus in the new paragraph

Answer: I added the new paragraph based on this comment.

Line 60: the largest

Answer: I’m now using “the largest” instead of “a largest”.

Line 61: Strikethrough text

Answer: I deleted the strikethrough text, and all the others through the manuscript file.

Line 63: I would suggest rearranging this sentence for ease of reading. Two of the units, the Mecca.....and the Logan... are from Parke County, Indiana.

Answer: I rephrased this sentence as follow: Two of the units, the Mecca Quarry Shale member of the Linton Formation overlayed on coal IIIA (Colchester Coal) and the Logan Quarry Shale member of the Staunton Formation below coal III (Seelyville Coal), are from the Parke County, Indiana (Fig. 2).

Line 70: than the Mecca Quarry shale?

Answer: Yes, the fossils from Logan Quarry Shale are better preserved than the Mecca Quarry Shale. I inserted “than the Mecca Quarry Shale” in the sentence.

Line 73: the

Answer: I added “the” in the sentence.

Line 73: indicates

Answer: I replaced the word following this comment.

Line 75: from

Answer: I added “from” in this sentence.

Line 92: as

Answer: I now use “as” instead of “with”.

Line 92: show

Answer: I replaced “showing” with “show” here.

Line 100: I suggest rearranging so that it reads

Among them, cartilaginous fish fossils such as.... have already been recognized and studied.

Answer: I rephrased this sentence: Among them, cartilaginous fish fossils such as *Deanea meccaensis* (Williams, 1985), *Rhombacanthus zangerli* (Williams, 1985), *Ornithoprion hertwigi* (Zangerl, 1966), and *Cobelodus aculeatus*, have already been recognized and studied (Zangerl & Case, 1976). With the exception of Poplin (1978), which described a specimen with a long rostrum based on X-ray radiograph, actinopterygians from the Logan Quarry have not been closely studied.

Line 103: I suggest rearranging to have something like:

With the exception of Poplin (1978) which described a specimen with a long rostrum based on a X-ray radiograph, actinopterygians from the Logan Quarry have not been closely studied.

Answer: Please check my answer for the last comment.

Line 106: The study of actinopterygian...

Answer: I did the rephrase following this comment.

Line 107: I suggest adding a sentence here to introduce that actinopterygians are the focus of this paper.

Accordingly, fossil actinopterygian fish are studied and presented here.

Answer: I added this sentence at the end of this paragraph.

Line 111: I suggest limiting the use of first person words like "I", "my" in a manuscript unless removing those terms drastically changes what is being said.

Answer: I corrected here and all other places through the text.

Line 113: extends the known time range

Answer: I rewrote this sentence following this comment.

Line 113: is described

Answer: I replaced the words in the text following this comment.

Line 114: expands

Answer: I use “expands” instead of “expand” here.

Line 118: on

Answer: The word “on” is used now.

Line 124: Materials and Methods

Answer: This heading is corrected now.

Line 125: studied

Answer: I added “studied” in the sentence.

Line 129: I suggest writing out ammonium chloride

Answer: I wrote ammonium chloride and deleted the chemical formula.

Line 130: observation of structures

Answer: I rephrased this sentence following this comment and deleted the strikethrough text.

Line 138: using the windows-based version of PAUP

Answer: I deleted the strikethrough text and replaced with “windows-based version” in front of “PAUP”.

Line 148: I suggest adding in that it is based on fig. 2 from Esin

Answer: I added (Fig. 2) in the text.

Line 156: U.S.A.

Answer: I corrected this typo.

Line 158: can shorten this by trying to give more of a telegraph style that would be in a diagnosis. Some examples given in comment bubbles

Answer: Now I use the telegraph style in the diagnosis paragraph.

Line 160: median dorsal rostral

Answer: I corrected the terms “median dorsal rostral” and “median ventral rostral” here and other places in the text.

Line 160: median ventral rostral

Answer: Same answer as above.

Line 161: elongated parietal;

Answer: I changed the words and following this comment.

Line 167: above you have ventral rostral as a separate bone from premax. Cannot have ventral rostro-premaxilla and median ventral rostral and paired premaxillae. Figure shows ventral rostral and separate premax. This needs to be checked and updated

Answer: I corrected and updated the diagnosis.

Line 168: highlighted text

Answer: Same answer as above.

Line 184: fusiform

Answer: I use fusiform fish instead of “medium sized fish” now.

Line 185: lengths of each specimen based on what is preserved were taken and used to estimate standard lengths. FMNH PF-3386....

Answer: I rewrote this part: Both the FMNH PF-2286 and the FMNH PF-5919 specimens are incomplete; lengths of each specimen based on what is preserved were taken and used to estimate standard lengths. FMNH PF-2286 is 160 mm long from the snout to the posterior, before the epaxial base of caudal fin, whereas FMNH PF-5919 is 150 mm long, including the posterior half of the skull and the body trunk until the squamation hinge line. Though FMNH PF-2286 seems longer, FMNH PF-5919 is more incomplete and missing portions anteriorly and posteriorly. Based on this, it is estimated that the standard length of FMNH PF-5919 would have been slightly longer than FMNH PF-2286.

Line 188: Though FMNJ PF-2286 seems longer, FMNH PF-5919 is more incomplete and missing portions anteriorly and posteriorly. Based on this, it is estimated that the standard legnth of FMNJ PF-5919 would have been slightly longer than FMNH PF-2286.

Answer: Same answer as above.

Line 192: estimated

Answer: It is written “estimated length” now.

Line 195: need to add pterygial formula of Westoll 1944 to methods and a description of what this is.

Answer: I added a brief description of the scale formula in the Methods section and Westoll 1944 is cited.

Line 202: here and throughout I suggest calling it a median dorsal rostral and a median ventral rostral bone to follow terminology of papers you cited for terminology

Answer: I corrected these terminologies.

Line 203: update diagnosis with ventral-rostro premaxillo mentioned

Answer: I fixed the diagnosis.

Line 213: I am not sure if crescent shaped matches what is shown in the illustration. Is there a better term to describe this shape?

Answer: Now I use “triangular” to describe the shape of nasal bone.

Line 217: Can you give a more detailed description of the ganoine ornamentation on these bones? Directions of ganine ridges? Fig. 4 shows a distinct pattern with long vertical ridges running the length of the nasal bone.

Answer: I added the description of the ornamentation here so that now the sentence reads: In the nasal bone the ornamentation ridges in dorsal portion are longer and more vertically placed, whereas in ventral portion, the ridges are shorter and horizontally placed.

Line 220: diagnosis says ventro-rostro premaxilla is toothed. Update diagnosis or this.

Answer: I corrected the diagnosis.

Line 229: sup to match label on figure

Answer: This typo is fixed in the figure caption.

Line 229: Add in description of what dashed lines represent and double dashed lines

Answer: I added the description in the caption.

Line 231: Can you explain why you are using this term over dermopterotic or supratemporal?

Answer: I use supratemporotabular instead of dermopterotic because I’m following the terminology of the skull bones from Schultze (2008).

Line 231: highlighted text

Answer: Same answer as above.

Line 265: in the studied specimens.

Answer: I added “in the studied specimens”.

Line 284: Best to remove ventral here since ventral dermosphenotic was a term used by Poplin (2004). You do not have a ventral dermosphenotic and a dorsal dermosphenotic here so best to remove use of ventral here.

Answer: I corrected the use of dermosphenotic through the text.

Line 294: Can you add in a description of the ganoine ornamentation on these bones?

Answer: None of the studied specimens has ganoine ornamentation preserved in the circumorbital bones. I added this sentence to the manuscript to clarify this situation.

Line 302: remove, do not have ventral dermosphenotic and a dorsal dermosphenotic

Answer: Corrected.

Line 323: Can you add a description of the features on the medial side of the maxilla? It is figured but then the horizontal longitudinal lamina is not mentioned in text

Answer: I added the description in the text. Now it reads: The medial surface of the maxilla is almost flat and smooth. The horizontal longitudinal lamina is preserved in the medial surface as a deep groove, which is horizontally across the suborbital arm and the ventral part of the posterior plate. The horizontal longitudinal lamina is ended slightly anterior than the origin of the posteroventral process.

Line 331: unknown

Answer: I fixed the typo. Now it’s “unknown”.

Line 351: or

Answer: I deleted “neither” and “nor” and added “or”.

Line 355: level

Answer: I use “level” to replace “horizon”.

Line 362: There are some big differences in the margins of the opercle and the subopercle between figures 6, 8, and 10. The ventral margin does not look very concave in fig. 10, especially if the margin of the suboperculum is as concave as shown in fig. 10 and 8. This is opposite what is shown in figure 6. Can you add a description addressing this? Is it preservational and if so, which is most complete?

Answer: The holotype has the most complete opercle. Consequently, the description is more based on Figure 6. A sentence to explain this issue is added in the text.

Line 366: can you add descriptions of the ganoine ornamentation of these bones?

Answer: I added the description of ornamentation. Now the text reads as follow: All the bones described above are ornamented with tubercles and short ridges of ganoine. The preopercle only has ornamentation preserved in a small area on the anterodorsal branch in FMNH PF-5919 (Figs. 9, 10). The ornamentation consists of short ridges and some small tubercles. The dermohyal is ornamented by several ridges of ganoine that are placed parallel to the longitudinal axis of this bone (Figs. 9, 10). The opercle and subopercle are heavily ornamented by short ridges and tubercles of ganoine (Figs. 7, 8). None of the branchiostegal rays have preserved the complete ornamentations. Most of the preserved ornamentations are on the anterior or posterior ends of the rays and consist of some dense short ridges of ganoine. These ridges are parallel to the longitudinal axis of the branchiostegal rays.

Line 373: Can you give more of a description-ridges, tubercles, direction, which specimen(s) show this?

Answer: Same answer as above.

Line 385: *Kalops* to avoid starting a sentence with an abbreviation

Answer: I corrected this style here and other places in the text.

Line 415: studied

Answer: I removed “available” and used the word “studied”.

Line 433: You explain this in the figure legend but it may be helpful to add to the text here that the regions are being described using the criteria of Esin described in figure 5.

Answer: I wrote the following sentence at the beginning of this paragraph: The regions of the scales described here are following the criteria of Esin (1990) (see Fig. 4).

Line 491: this

Answer: I replaced the word following this comment.

Line 493: species

Answer: I rewrote as “two Bear Gulch *Kalops* species” here.

Line 501: This wasn't seen on the Logan quarry specimens?

Answer: The pectoral fin base is very poorly preserved in *Kalops loganensis*. This character was coded with a question mark in the matrix.

Line 509: all other nodes came out with the same synapomorphies as Giles et al. 2017?

Answer: The complete node support is now in the new Supplementary information1 file (S1_File.pdf).

Line 522: emphasized

Answer: I rewrote as “emphasized”.

Line 523: an

Answer: I added “an” in the text.

Line 532: bones.

Answer: Added in the text.

Line 533: the

Answer: Added in the text.

Line 534: *Kalops*

Answer: Corrected here.

Line 538: *Kalops*

Answer: Corrected here.

Line 546: is absent

Answer: Replaced the words following this comment.

Line 546: both species from the Bear Gulch

Answer: I replaced the words with: “Both species from the Bear Gulch”

Line 560: which tree in Cloutier and Arratia 2004?

Answer: It’s based on fig. 10A of Cloutier and Arratia (2004), and this information was added in the text.

Line 570: the hypotheses of relationships recovered in the analysis presented here are more in agreement...

Answer: I rewrote this sentence to match the comment.

Line 571: How similar are the taxa investigated in these phylogenetic analyses? Are there different recovered hypotheses of relationships because different taxa are included in the different analyses?

Answer: I added more explanations so that now the sentence read: In summary, although using different sets of characters, the hypotheses of relationships recovered in the analysis presented here are more in agreement with those of Cloutier & Arratia (2004) than other studies, despite the fact that the parameters, such as the selection of taxa and characters, and the outgroup taxon used in both works are different.

Line 573: Serpukhovian

Answer: I corrected the typo.

Line 587: Fielding 2021 in references. check which is right.

Answer: It’s Fielding 2021, and I fixed it here.

Line 593: lifestyles

Answer: I rewrote and used “lifestyles”.

Line 595: survived

Answer: I corrected this type.

Line 596: rise

Answer: I fixed this type.

Line 603: a

Answer: It is added in the text.

Line 611: and

Answer: I fixed this typo.

Line 718: Listed as Mastalerz & Harper 1988 in the in-text citations

Answer: The corrected citation is Mastalerz & Harper 1998. It’s fixed. Now all the citations are following the instructions of the journal.

Line 771: 2015 cited in text?

Answer: I changed the year to 2016 in the text.

Line 775: 2022 cited in text

Answer: I changed the year of this citation to 2022.

Figure 16: The text is a little hard to read. Can you submit for the final version a better quality, higher resolution version? You may need to retype the taxa names in illustrator or another program so that they are clearer.

Answer: I retyped the taxa names in Figure 16 via Illustrator. They are more readable now. The resolution of this figure is 600 dpi. So the letters will be clear if enlarge this figure.

**Response the comments from Reviewer 2:**

Line 35: May be better to say the fauna had turned over, or some similar phrasing - the armored fishes were decimated by the End-Devonian extinction, essentially replaced by the rapidly diversifying actinopts and chondrichthyans. Make it clear that's the case. As written, sounds more like these groups weathered and then re-diversified.

Answer: I rephrased this sentence as: Recent studies suggest that the marine vertebrate ecosystem had turned over and established a high diversity of both bony and cartilaginous fishes by the end of the Mississippian (Early Carboniferous).

Line 39: Watch out for typos

Answer: I corrected the typo, now it is vertebrates.

Line 44: This needs rephrasing, maybe "Chondrichthyan species identified from Bear Gulch have also been identified from additional coeval localities (e.g., Harpagofututor)... "

Answer: I rephrased this part as: Among the vertebrates, the chondrichthyan species identified from Bear Gulch have also been identified from additional coeval localities (Grogan *et al*. 2012), such as the Euro-North American *Harpagofututor volsellorhinus* (Lund, 1982), and *Thrinacoselache gracia* (Grogan & Lund, 2008).

Line 54: Do you mean we don't understand other aspects of Kalops' biology? Because otherwise our understanding of its geological and time distribution is the same as any fish spp. only known from a single locality...

Answer: I introduced a new paragraph to describe the biological history of *Kalops*: So far, there are two species of *Kalops* described, *K. monophrys* and *K. diophrys*, and both are excavated from the Bear Gulch Limestone (Poplin & Lund, 2002). This genus is characterized by an elongated body form, subterminal mouth with the oral margin of the premaxilla facing posteroventrad, dorsal and ventral median rostral bones, short dermosphenotic, multiple small supraorbitals, infraorbitals, and suborbitals. Poplin & Lund (2002) summarized three most distinguishable features of *Kalops* than other “paleoniscoid” fish: numerous supraorbitals, numerous suborbitals, and an accessory row of extrascapulae anterior to the main row. However, compare to the anatomical information on *Kalops*, we know very little about the geographical and time distribution of this genus.

Line 61: For folks outside North America, it would be better to just include an estimate of maybe sq kilometers?

Answer: The square kilometers of the NAMS is added. Now the sentence reads: The 1-2-meter-thick shale is the evidence of the North American Mid-continent Sea (NAMS), which existed from Mid-Pennsylvanian to Early Permian, with the largest extension about 2,100,000 km^2^, nearly twice as large as today’s Hudson Bay (Algeo & Heckel, 2008).

Line 61: grammar

Answer: I changed the word to “western”.

Line 73: grammar

Answer: I fixed this sentence as: The light-yellow highlighted area indicates the Illinois Basin.

Line 77: grammar

Answer: I rewrote as “localities of the Black Shales”.

Line 79: if this is a formally recognized shale member, then it should be capitalized as it is elsewhere in the manuscript

Answer: It should be capitalized. I fixed this one and others through the text.

Line 81: Do you mean "sheet shale"? Describing the way the shale's layers are deposited?

Answer: I removed the “sheety” in the text.

Line 83: This doesn't make sense as written... do you mean the fossil assemblage contains fossils of both nearshore marine and freshwater origin?

Answer: Yes. There are both marine and freshwater fossils in the Logan Quarry.

Line 89: Strikethrough text

Answer: I deleted the strikethrough text here and all other strikethrough text in the file.

Line 92: I'm confused by this sentence. Are you saying the flora/fauna indicate brackish water? or are you saying that the composition of the sediment is indicative of brackish water?

Answer: I mean this type of sediment indicates brackish water. I also clarified this in the sentence.

Line 98: and "a pleuracanthid?" who?

Answer: It is just called “pleuracanthid” from Zangerl & Richardson (1963) book. No further studies have been mapped to this taxon.

Line 113: range

Answer: I use “known time range” instead of “temporal” now.

Line 126: spelling

Answer: It is fixed.

Line 136: Don't send your reader over to Sam's paper to review her parameters. Restate them.

Answer: I added the information to the phylogenetic method section. I also created a new Supplementary information 1 file. It includes all the characters descriptions, and the nodes supports for my phylogenetic tree.

Line 139: This paragraph needs to be re-ordered. Lines 136-137 should come first, and lines 134-135 should come last. Introduce that Giles et al. performed that study, then say you added new species to their matrix and estimated a phylogeny in PAUP, then say it was unweighted parsimony.

Answer: I rewrote this paragraph. Now it reads: Giles et al. (2017) performed a comprehensive phylogenetic analysis of early Osteichthyes by using 265 morphological characters, and both *K. monophrys* and *K. diophrys* were included in the analysis. The phylogenetic analysis was conducted by adding the new species into the matrix of Giles *et al*. (2017). The settings of the characters from Giles et al. (2017) are kept the same in this analysis, so that all characters are equally weighted, 258 characters are unordered, whereas seven characters are ordered (characters 89, 91, 151, 171, 241, 246, and 251). For the list of characters, see Supplementary Material 1. *Acanthodes bronni* is selected as the outgroup. The parsimony analysis was conducted using the windows-based version of PAUP (4.0a, build 169).

Line 140: Did you test whether equal weights parsimony was the best model for your dataset? Or can you at least mention whether Giles et al. did so? (For morphology, yeah it's usually best, but I do think it's good to document that you compared other methods)

Answer: I added the explanation in this paragraph that the equally weighted characters are just following the setting from Giles et al. (2017).

Line 159: Total length?

Answer: I rewrote here as 160 mm long.

Line 160: I'm not as well-versed in paleoniscoid terminology, but is this the bone Lund would have called the postrostral?

Answer: This bone was called the postrostral by Lund. But I’m following the terminology of Mickle (2012), where this bone is defined as median dorsal rostral.

Line 184: Living fishes range from 12 mm to 20 m as adults! This sentence should be discarded.

Answer: I rewrote this sentence. Now it is “this taxon is a fusiform fish”.

Line 189: Confusing as written

Answer: I rephrased this sentence: Though FMNH PF-2286 seems longer, FMNH PF-5919 is more incomplete and missing portions anteriorly and posteriorly. Based on this, it is estimated that the standard length of FMNH PF-5919 would have been slightly longer than FMNH PF-2286.

Line 194: I think you need to reorganize these sentences

Answer: I reorganized these sentences as follow: The skull dermal bones are covered with ganoine. The body is fully covered by ganoid-type of scales. The caudal fin is heterocercal.

Line 212: horizontally across the midline of the ventral rostral?

Answer: I rephrased this sentence: The ethmoid commissure is placed horizontally across the median ventral rostral.

Line 216: Probably not needed - aren't they almost always? Answer:

Answer: Sometimes they are at the same level. Thus, I will keep this part.

Line 219: "running posteriorly to contact the lacrimal"

Answer: I rephrased this sentence: The premaxilla is small, shallow and elongated antero-posteriorly. The posterior margin of this bone contacts the lacrimal.

Line 237: These statements conflict - what do you mean by the sutures are unclear? Sutures in general? Or sutures just surrounding the pineal opening??

Answer: I mean the type of the sutures are unclear, and the position of the pineal opening is unclear as well. I rewrote this sentence.

Line 236: rewrite as a ratio

Answer: I rewrote as a ratio: 2.8:1.

Line 248: You need to be continually referring to the figures throughout the descriptive part of the paper.

Answer: I fixed it here and added references to the figures through the text.

Line 266: Not preserved?? or obscured by dermal bone? Should be more clear about that

Answer: I added “obscured by dermal bones” to explain this situation.

Line 266: two infraorbitals

Answer: The bone is called infraorbital two. It is the infraorbital bone between the lacrimal (infraorbital one) and the jugal (infraorbital three).

Line 292: Again, please refer to figures throughout the description

Answer: I added (Fig. 6) at the end of this paragraph.

Line 301: dorsally or ventrally?

Answer: I added a short description: In the jugal the infraorbital canal bends dorsad.

Line 314: Do you mean 2:3?

Answer: I rewrote as a ratio: 2.3:1.

Line 331: typo

Answer: I corrected it.

Line 365: rephrase

Answer: I rephrased this description: The posterior margin of the subopercle is longer than the anterior margin, being its dorsal margin an oblique line, which is also concave.

Line 405: Attached to its base?? Do you just mean it's an actinopt? What kind of lobe structure would be attached to the base of a fish's fin?

Answer: According to other authors, the scale lobe structure at the base of the pectoral fin evolved multiple time in Actinopterygii, such as *Aesopichthys* (Poplin & Lund, 2000), *Australosomus* (Nielsen, 1949), *Birgeria* (Nielsen, 1949), *Boreosomus* (Nielsen, 1942), *Cheirolepis* (Pearson & Westoll, 1979; Arratia & Cloutier, 1996; Giles et al. 2015), *Discoserra* (Lund, 2000), *Dorsetichthys* (Patterson, 1968; 1973; 1975; Grande & Bemis, 1998), *Evenkia* (Berg, 1942; Selenzneva, 1985; Sytchevskaya, 1999), *Fouldenia* (Sallan & Coates, 2013), *Howqualepis* (Long, 1988), *Kalops* (Poplin & Lund, 2002), *Lepisosteus* (Grande, 2010), *Osorioichthys* (Taverne, 1997), *Platysomus* (Moy-Thomas & Bradley-Dyne, 1938), *Polypterus* (Allis, 1922; Grande, 2010), *Saurichthys* (Kogan & Romano, 2016), and *Tegeolepis* (Dunkle & Schaeffer, 1973).

References only for this answer:

Allis Jr, E. P. (1922). The cranial anatomy of Polypterus, with special reference to Polypterus bichir. *Journal of Anatomy*, *56*(Pt 3-4), 189.

Arratia, G., & Cloutier, R. (1996). Reassessment of the morphology of Cheirolepis canadensis (Actinopterygii). *Devonian fishes and plants of Miguasha, Quebec, Canada*, 165-197.

Berg, L. S. (1942). Lower Triassic fishes of the Tunguska coal basin, Yenisei, Siberia. *Bulletin of the Academy of Sciences USSR, Biological Sciences*, *3*, 414-419.

Dunkle, D. H., & B. Schaeffer. 1973. *Tegeolepis clarki* (Newberry), a palaeonisciform from the Upper Devonian Ohio Shale. Palaeontographica Abteilung A, 143:151–158.

Giles, S., Coates, M. I., Garwood, R. J., Brazeau, M. D., Atwood, R., Johanson, Z., & Friedman, M. (2015). Endoskeletal structure in C heirolepis (O steichthyes, A ctinopterygii), An early ray‐finned fish. *Palaeontology*, *58*(5), 849-870.

Grande, L. (2010). An empirical synthetic pattern study of gars (Lepisosteiformes) and closely related species, based mostly on skeletal anatomy. The resurrection of Holostei. *Ichthyology & Herpetology*, *10*(2A), 1-871.

Grande, L., & Bemis, W. E. (1998). A comprehensive phylogenetic study of amiid fishes (Amiidae) based on comparative skeletal anatomy. An empirical search for interconnected patterns of natural history. *Journal of Vertebrate Paleontology*, *18*(sup1), 1-696.

Kogan, I., & Romano, C. (2016). Redescription of Saurichthys madagascariensis Piveteau, 1945 (Actinopterygii, Early Triassic), with implications for the early saurichthyid morphotype. *Journal of Vertebrate Paleontology*, *36*(4), e1151886.

Long, J. A. 1988. New palaeoniscoid fishes from the Late Devonian and early Carboniferous of Victoria. *Memoir of the Australasian Association of Palaeontologists* **7**: 1–64.

Lund, R. 2000. The new actinopterygian order Guildayichthyiformes from the Lower Carboniferous of Montana (USA). *Geodiversitas* **22**: 171–206.

Moy-Thomas, J. A., and Bradley-Dyne, M. 1938. XVII.—The Actinopterygian Fishes from the Lower Carboniferous of Glencartholm, Eskdale, Dumfriesshire. *Transactions of the Royal* *Society of Edinburgh* **59**: 437–480.

Nielsen, E. (1942). Studies on Triassic Fishes from East Greenland I: *Glaucolepis* and *Boreosomus*. *Meddelser øm Grønland 146*: 1–309.

Nielsen, E. (1949). Studies on Triassic Fishes from East Greenland II: *Australosomus* and *Birgeria*. *Meddelser øm Grønland 146*: 1–309.

Patterson, C. 1968. The caudal skeleton in Lower Liassic pholidophorid fishes. Bulletin of the British Museum Natural History (Geology) **16**: 203–239.

Patterson, C. 1973. Interrelationships of holosteans. 233–306. *In* P. H. GREENWOOD, P. H., MILES, R. S. and PATTERSON, C (eds). *Interrelationships of Fishes* Academic Press, New York, 536 pp.

Patterson, C. 1975. The braincase of pholidophorid and leptolepid fishes, with a review of the actinopterygian braincase. *Philosophical Transactions of the Royal Society of London, Series B* **269**: 275–579.

Pearson, D. M., & Westoll, T. S. (1979). The devonian actinopterygian cheirolepis agassiz. *Earth and Environmental Science Transactions of the Royal Society of Edinburgh*, *70*(13-14), 337-399.

Poplin, C., & Lund, R. (2000). Two new deep-bodied palaeoniscoid actinopterygians from Bear Gulch (Montana, USA, Lower Carboniferous). *Journal of Vertebrate Paleontology*, *20*(3), 428-449.

Poplin, C., & Lund, R. (2002). Two Carboniferous fine-eyed palaeoniscoids (Pisces, Actinopterygii) from Bear Gulch (USA). *Journal of Paleontology*, *76*(6), 1014-1028.

Sallan, L. C., & Coates, M. I. (2013). Styracopterid (Actinopterygii) ontogeny and the multiple origins of post-Hangenberg deep-bodied fishes. *Zoological Journal of the Linnean Society*, *169*(1), 156-199.

Selezneva, A. A. 1985. *Evenkia* is *Polypterus* ancestor. *Paleotologisheskii Zhurnal* **19**: 71–76. [in Russian]

Sytchevskaya, E. K. (1999). Freshwater fish fauna from the Triassic of Northern Asia. *Mesozoic fishes*, *2*, 445-468.

Taverne, L. (1997). Osorioichthys marginis,“Paléonisciforme” du Famennien de Belgique, et la phylogénie des Actinoptérygiens dévoniens (Pisces). *Bulletin de l'institut Royal des Sciences Naturelles de Belgique*, *67*, 57-78.

Line 416: There must be a pelvic girdle there, and this sentence makes it sound like they're absent! Just say "the pelvic girdle is obscured by scales."

Answer: I rewrote the beginning of this paragraph: There is no pelvic girdle observable in the studied specimens because that area is obscured by the scales.

Line 419: Lost to taphonomy? Or absent? Be clear

Answer: I rewrote here as “the anal fin is not preserved on all specimens”.

Line 484: What does this mean?

Answer: I rewrote as: Note that the size of the scales is not under the same enlargement.

Line 492: typo

Answer: It is fixed.

Line 494: Wait, so then it's not a synapomorphy. It can be supported by those characters, but they are not synapomorphies if they're homoplastic

Answer: Synapomorphy means shared derived features. Both uniquely derived features and homoplastic features are synapomorphies.

Line 511: Not needed in a figure caption

Answer: This part is removed.

Line 563: This sentence is very confusing - do you mean the Bear Gulch Kalops species??

Answer: I mean all the actinopterygian species from the Bear Gulch. Rephrased this sentence: Some other studies suggested that the Bear Gulch actinopterygian species are not closely related to each other.

Line 577: rephrase

Answer: I removed this part since it’s not very important.

Line 583: Do you mean repeated presence of stratigraphically similar layers?

Answer: Yes. I rephrased this sentence.

Line 593: What? Do you mean it's epipelagic??

Answer: Yes. I use epipelagic to rephrase this sentence.

Line 594: again, this is not a commonly used phrase and is confusing

Answer: I rephrased this sentence.

Figure 1: Where was this gleaned from? THe labels should be larger, maybe even highlighted with some color to show the extent of the NAMS and IB

Answer: I bought the base map with permission to publish from Colorado Plateau Geosystems Inc. In the revised version, I highlighted the Illinois Basin and enlarged the font size.
